# Supplementary figures and images for: Adiposity measures and arterial stiffness in primary care: the MARK prospective observational study
Source: BMJ Open. 2017 Sep 27;7(9):e016422. doi: 10.1136/bmjopen-2017-016422 (PMC5623460; doi:10.1136/bmjopen-2017-016422)

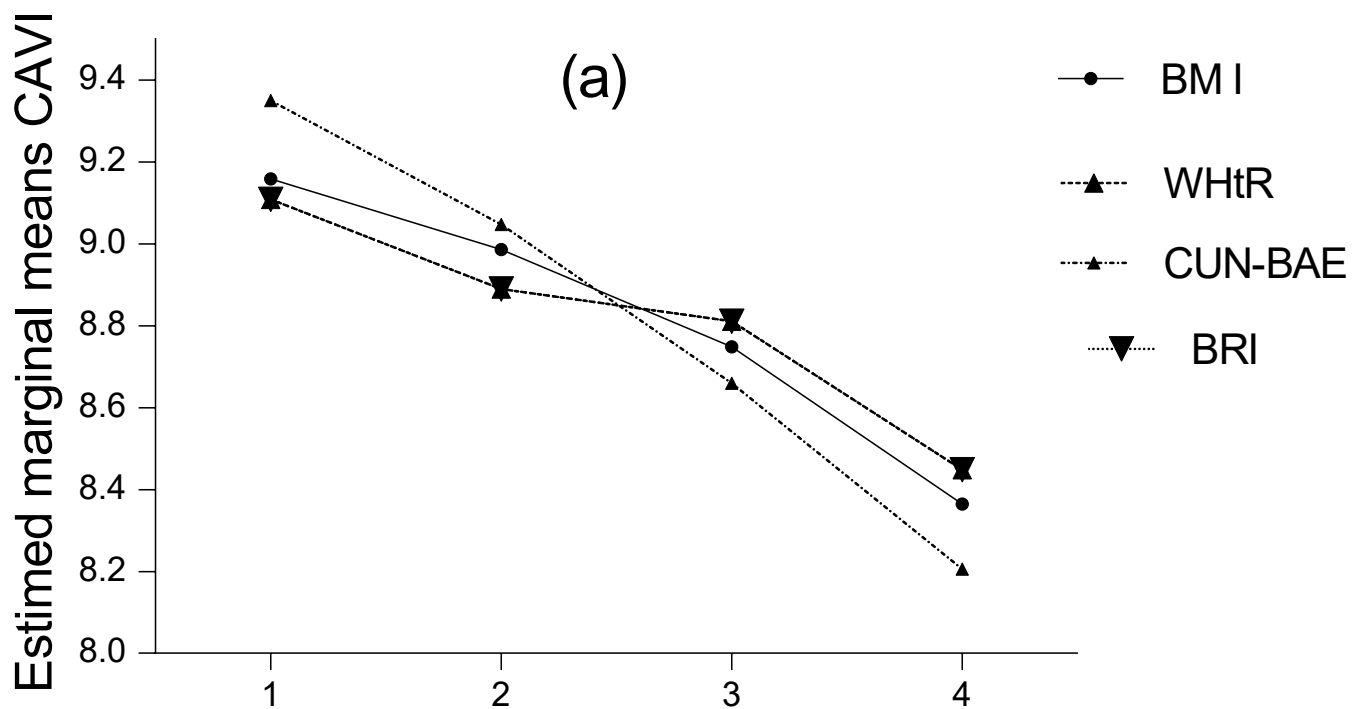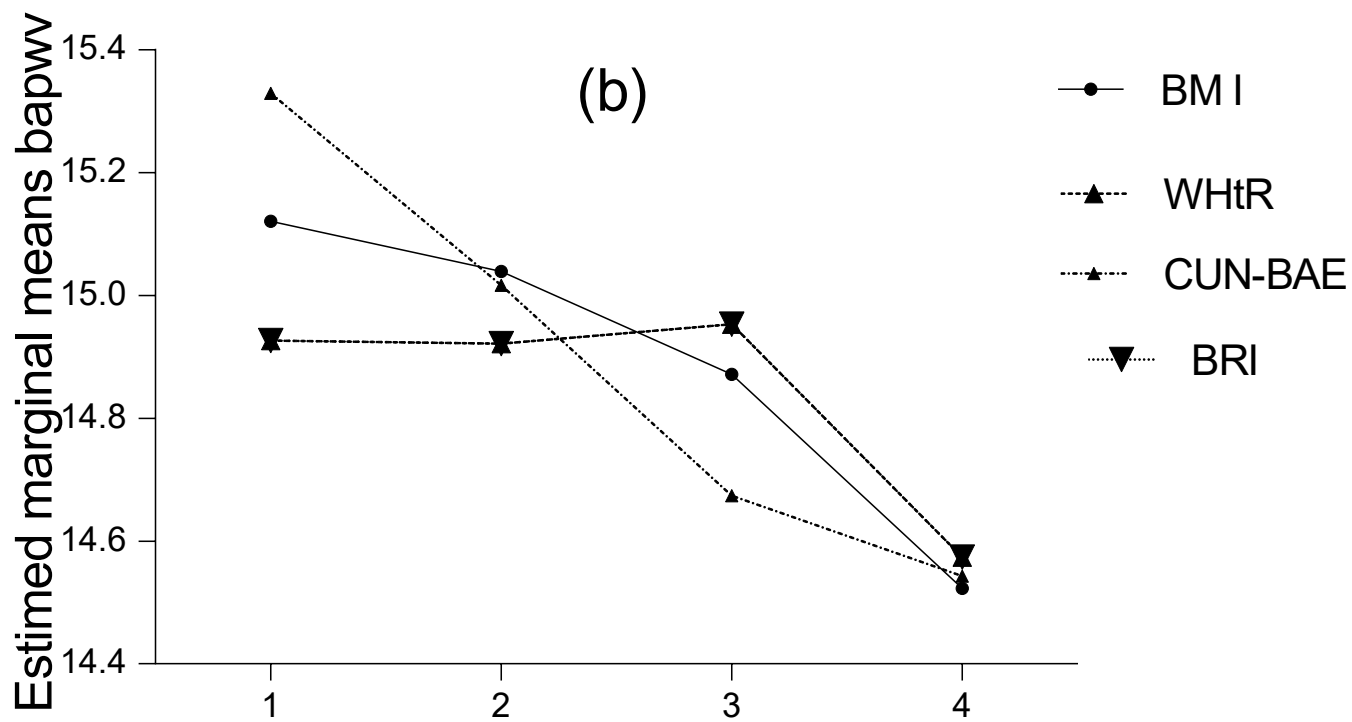

Supplement: Supplementary file 1 [file bmjopen-2017-016422supp001.pdf]
