# Supplementary material for: Adiposity measures and arterial stiffness in primary care: the MARK prospective observational study
Source: BMJ Open. 2017 Sep 27;7(9):e016422. doi: 10.1136/bmjopen-2017-016422 (PMC5623460; doi:10.1136/bmjopen-2017-016422)
Supplement: Author's manuscript [file bmjopen-2017-016422.draft_revisions.pdf]

BMJ Open is committed to open peer review. As part of this commitment we make the peer review history of every article we publish publicly available.

When an article is published we post the peer reviewers' comments and the authors' responses online. We also post the versions of the paper that were used during peer review. These are the versions that the peer review comments apply to.

The versions of the paper that follow are the versions that were submitted during the peer review process. They are not the versions of record or the final published versions. They should not be cited or distributed as the published version of this manuscript.

BMJ Open is an open access journal and the full, final, typeset and author-corrected version of record of the manuscript is available on our site with no access controls, subscription charges or pay-per-view fees (http://bmjopen.bmj.com).

If you have any questions on BMJ Open's open peer review process please email editorial.bmjopen@bmj.com

Title: Adiposity measures and arterial stiffness: the MARK prospective observational study.

Journal:	*BMJ Open*
Manuscript ID	bmjopen-2017-016422
Article Type:	Research
Date Submitted by the Author:	13-Feb-2017
Complete List of Authors:	Gomez-Sanchez, Leticia<br>Garcia-Ortiz, Luis; La Alamedilla health centre. University of Salamanca, Primary care research unit La Alamedilla<br>Patino-Alonso, Maria<br>Recio-Rodriguez, Jose; La Alamedilla Health Center, Primary Care Research Unit, The Alamedilla Health Center, Castilla and León Health Service (SACYL), Biomedical Research Institute of Salamanca (IBSAL)<br>Frigo, Fernando<br>Marti, Rhut<br>Agudo-Conde, Cristina; La Alamedilla Health Center,, Research Unit<br>Rodriguez-Sanchez, Emiliano; Primary Care Research Unit, The Alamedilla Health Center, Salamanca, Spain, ; Biomedical Research Institute of Salamanca (IBSAL),<br>Maderuelo-Fernandez, Jose; Salamanca Institute for Biomedical Research. (IBSAL)., Primary care research unit La Alamedilla, Castilla and León Health Service (SACYL). Network of Preventive Activities and Health Promotion in Primary Care (REDIAPP)<br>Ramos, Rafel; Universidad de Girona. Departament Medicina<br>Gomez-Marcos, Manuel
**Primary Subject Heading**:	Cardiovascular medicine
Secondary Subject Heading:	Diabetes and endocrinology, Epidemiology, Nutrition and metabolism
Keywords:	Body mass index, Waist circumference, Waist-to-height ratio, Fat mass percent., Body roundness index., Arterial stiffness

Title: Adiposity measures and arterial stiffness: the MARK prospective observational study.

Authors: Leticia Gomez-Sanchez MD [1]*, Luis Garcia-Ortiz MD, PhD [1,2,3], Maria C Patino-Alonso PhD [1,2,4], Jose I Recio-Rodriguez PhD [1,2,5], Rigo Fernando MD, PhD [6], Ruth Marti PhD [7,8], Cristina Agudo-Conde [1,2], Emiliano Rodriguez-Sanchez MD, PhD [1,2,9], Jose A Maderuelo-Fernandez MD, PhD [1,2], Rafel Ramos MD, PhD [7,8,10] and Manuel A Gomez-Marcos MD, PhD [1,2,9] for the MARK Group [11]

1. Primary Care Research Unit, the Alamedilla Health Center, Castilla and León Health Service (SACyL), Salamanca, Spain.
2. Biomedical Research Institute of Salamanca (IBSAL), Salamanca, Spain.
3. Biomedical and diagnostic sciences department, University of Salamanca, Salamanca, Spain.
4. Statistics Department, University of Salamanca, Salamanca, Spain.
5. Department of Nursing and Physiotherapy, University of Salamanca, Salamanca, Spain.
6. San Agustín Health Center, Illes Balears Health Service (IBSALUT), Palma of Mallorca, Spain.
7. Unitat de Suport a la Recerca de Girona. Institut Universitari d'Investigació en Atenció Primària Jordi Gol (IDIAP Jordi Gol), Girona, Spain.
8. Institut d'Investigació Biomèdica of Girona Dr. Josep Trueta (IDBGI). Girona, Spain.
9. Department of Medicine, University of Salamanca, Salamanca, Spain.
10. Departament of Ciències Mèdiques, Facultat de Medicina. Universitat de Girona, Spain.
11. MARK Group. redIAPP: Research Network in Preventive Activities and Health Promotion, Girona, Spain.

***Corresponding Author:**

Leticia Gomez-Sanchez, Primary Care Research Unit, Alamedilla Health Care Center.

37003 Salamanca, Spain.

Full list of author information is available at the end of the article.

For peer review only - http://bmjopen.bmj.com/site/about/guidelines.xhtml

ABSTRACT

Background: Increased arterial stiffness is one possible mechanism of increased cardiovascular risk from obesity.

Objective: This study investigates the relationship of adiposity measures with arterial stiffness in Caucasian adults with intermediate cardiovascular and determines measures with greater power to explain arterial stiffness.

Setting: 6 primary care centres in in three Spanish Autonomous Communities, Spain.

Participants: This study analyzed 2354 subjects (age 35–74 years (mean 61.4±7.7 years), 61.9% male).

Methods: The study was based on cross-sectional data from the MARK study. The main outcome variables were: body mass index (BMI), waist-to-height ratio (WHtR), University clinic of Navarra body adiposity estimation (CUN-BAE) body fat percentage, and body roundness index (BRI). Vascular function was assessed by the cardio-ankle vascular index (CAVI) with the VaSera device, and brachial ankle pulse wave velocity (baPWV) was determined using a validated equation.

Results: The mean adiposity measures were a BMI of 29.2±4.4, WHtR of 0.61±0.07, CUN-BAE of 35.7±1.7, and BRI of 5.8±1.7. The mean stiffness measures were a CAVI of 8.8±1.2 and baPWV of 14.9±2.5. In multiple linear regression analysis, all adiposity measures were negatively associated with CAVI and baPWV ($p<0.01$ in all) after adjustment for cardiovascular risk factors. The proportions of CAVI variability explained in the respective by the adiposity measures were 7.5% for BMI, 7.2% for CUN-BAE, 4.3% for WHtR, and 4.1% for BRI, which were higher among diabetic, obese, younger (≤62 years), and non-hypertensive subjects.

For peer review only

Conclusions: Adiposity measures are negatively associated with arterial stiffness measures. The explanatory capacity of the variability of arterial stiffness is higher for BMI and CUN-BAE and when using CAVI as a measure of stiffness rather than baPWV.

Trial Registration: Clinical Trials.gov Identifier: https://clinicaltrials.gov/ct2/show/NCT01428934. Registered 2 September 2011. Last updated September 8, 2016.

Keywords: Body mass index. Waist circumference. Waist-to-height ratio. Fat mass percent. Body roundness index. Arterial stiffness.

Strengths and limitations of this study

- This is the first study to investigate the association between adiposity measures with the cardio-ankle vascular index with and brachial ankle pulse wave velocity in Caucasian adults with intermediate cardiovascular risk.
- All adiposity measures were negatively associated with CAVI and baPWV.
- The explanatory capacity of the variability of arterial stiffness is higher for BMI and CUN-BAE and when using CAVI as a measure of stiffness rather than baPWV.
- The main limitation of our study is its cross-sectional design, which does not allow us to establish causal relations or the direction of influence of adiposity measures in vascular function.

For peer review only

INTRODUCTION

Obesity has been linked to increased all-cause and cardiovascular mortality [(1)]. However, the mechanisms through which obesity can increase the frequency of cardiovascular disease beyond traditional risk factors are not clearly identified [(2)]. Arterial stiffness as evaluated by the brachial ankle pulse wave velocity (baPWV) is an independent predictor of coronary heart disease and mortality in both the general population [(3)] and in patients with diabetes mellitus [(4; 5)]. Similarly, the cardio-ankle vascular index (CAVI) is associated with carotid and coronary atherosclerosis [(6; 7; 8)] and is a predictor of cardiovascular events in obese patients [(9)]. Increased arterial stiffness may be one of the mechanisms by which obesity increases cardiovascular risk independently of traditional risk factors. However, the relation between adiposity and arterial stiffness remains controversial. Thus the body mass index (BMI) has been associated with arterial stiffness in the general population [(10; 11)], and in diabetic patients [(12; 13)]. However, other works have not found this association [(14)], the association disappeared after adjusting for potential confounders [(13)], or showed a negative association [(15; 16)]. On the other hand, there are studies that suggest a greater correlation of measures of central or visceral adiposity than measures of general adiposity with arterial stiffness in the general populatio [(10; 17; 18; 19; 20)], in diabetic patients [(13)], and in diabetics and hypertensive patients [(21)]. The Whitehall II Cohort study [(22)], found that all measures of general adiposity, central adiposity, and body fat percentage were predictors of accelerated arterial stiffness in adults.

Cardiovascular events are more likely to occur in patients with intermediate cardiovascular risk [(23)], and there is a lack of studies analyzing the relationship of different adiposity measures with arterial stiffness in such subjects. Thus, the primary aim of this study is to investigate the relationship between adiposity measures and arterial stiffness in Caucasian adults with intermediate cardiovascular risk. The secondary aim is to determine which adiposity measures have greater power to explain arterial stiffness.

For peer review only - http://bmjopen.bmj.com/site/about/guidelines.xhtml

METHODS

Study design

This trial is a cross-sectional study of subjects recruited to the *improving interMediAte RisK management (MARK)* study (NCT01428934) [24], which is a longitudinal study designed to assess whether the ankle-brachial index, arterial stiffness (measured by CAVI), postprandial glucose, glycosylated hemoglobin, self-measured blood pressure, and the presence of comorbidities are independently associated with the occurrence of vascular events. It also investigates whether the predictive capacity of current risk equations can be improved in the intermediate risk population. The current study focuses on the baseline visit. The second step will be a 5- and 10-year follow-up trial to assess cardiovascular morbidity and mortality.

Study population

In this multicenter project, study population was performed by random sampling from subjects who met the inclusion criteria and were seeing general practitioners from July 2011 to June 2013 at six primary care centers in three Spanish Autonomous Communities. Subjects were recruited from those aged 35 to 74 years with intermediate cardiovascular risk, defined as 10-year coronary risk ranging from 5%–15% according to the adapted Framingham risk equation [25]; 10-year vascular mortality risk ranging from 1%–5% according to the scoring risk in Europeans equation [26]; or moderate risk according to the European Society of Hypertension guidelines for the management of arterial hypertension [27]. Exclusion criteria included end-stage disease or institutionalization at the time of the visit, or history of atherosclerotic disease. This study analyzed 2354 of the 2495 subjects recruited in the MARK study, and the grounds for exclusions are shown in figure 1.

Variables and measurement instruments

A detailed description of procedures for clinical data collection and laboratory tests has been published elsewhere [24].

For peer review only - http://bmjopen.bmj.com/site/about/guidelines.xhtml

Anthropometric measurements

Body weight was measured twice with a certified electronic scale (Seca 770, Medical scale and measurement systems, Birmingham, United Kingdom) after adequate calibration (precision ± 0.1 kg). Readings were rounded to 100 g. Height was measured with a stadiometer (Seca 222), and the average of two measurements was recorded. Body mass index (BMI) was calculated as weight (kg) divided by height squared (m^2). Waist circumference was measured according to the 2007 recommendations of the Spanish Society for the Study of Obesity [29]. All measurements were performed with the subjects standing, wearing no shoes, and in light clothing. Waist to height ratio (WHtR) was calculated as waist circumference (cm) divided by height (cm) [30; 31].

The body fat percentage (BF%) was calculated according University clinic of Navarra - body adiposity estimator (CUN-BAE) to the recommendations of Gomez-Ambrosi J et al [32]. BF%= -44.988+ (0.503 × Age) + (10.689 × gender) + (3.172 × BMI) - (0,026 × BMI^2) + (0.181 × BMI × gender) - (0.02 BMI × Age) - (0,005 × BMI^2 × gender) + (0.00021×BMI^2 × Age) wher win male=0 and female=1 for gender.

Body Roundness Index (BRI), was calculated using formula as BRI= 364.2 - 365.5 × $\sqrt{1-\frac{(WC/(2\pi))^2}{(0.5\text{height})^2}}$ [33]. BRI could predict the percentage of body fat and visceral adipose tissue.

Cardio-ankle vascular index (CAVI) and brachial-ankle pulse wave velocity (baPWV)

CAVI was measured using a *VaSera VS-1500®* device *(Fukuda Denshi)* [34; 35]. CAVI values are calculated automatically by estimating the stiffness parameter β with the following equation: β = 2ρ x 1 / (Ps - Pd) x ln (Ps / Pd) x PWV^2, where ρ is blood density, Ps and Pd are SBP and DBP in mmHg, and PWV is measured between the aortic valve and the ankle [36]. The mean coefficient of variation of CAVI measurement is less than 5%, which is small enough to allow for clinical use of the index and confirms that CAVI has a favorable

For peer review only - http://bmjopen.bmj.com/site/about/guidelines.xhtml

reproducibility [35]. baPWV was estimated using the equation, baPWV= (0.5934 × height (cm) + 14.4724)/tba (tba is the time interval between the arm and ankle waves) [37].

Measurements were performed with the patient in supine position after resting for 10 minutes in a quiet room at a stable temperature. Subjects were instructed not to smoke or practice exercise in the hour prior to the test.

Diagnosis of cardiovascular risk factors

Subjects were considered hypertensive if previously diagnosed with hypertension, if they were taking antihypertensive drugs, or if they had blood pressure levels ≥ 140/90 mmHg. Diabetic subjects were those who had a previous diagnosed of the disease, were taking hypoglycemic drugs, or had fasting blood glucose levels ≥ 126 mg/dL or HbA1c ≥ 6.5%. Dyslipidemia was defined as a prior diagnosis of the condition, use of lipid-lowering drugs, or fasting total cholesterol levels ≥ 250 mg/dL.

Office or clinical blood pressure: Office blood pressure measurement involved three measurements of SBP and DBP with a validated OMRON model M10-IT sphygmomanometer (Omron Health Care, Kyoto, Japan). The measurements followed the recommendations of the European Society of Hypertension [38], and the averages of the last two measurements were used.

Lifestyles

Tobacco: Smoking history was assessed by asking questions about the participant's smoking status (smoker/non-smoker). We considered smokers to include those who currently smoke or who have stopped smoking within the past year.

Laboratory determinations

Venous blood sampling was performed between 08:00 and 09:00 hours after the individuals fasted and abstained from smoking and the consumption of alcohol and caffeinated beverages for the previous 12 hours. Fasting plasma glucose, serum, high-density lipoprotein (HDL)

For peer review only - http://bmjopen.bmj.com/site/about/guidelines.xhtml

cholesterol concentrations and triglyceride concentrations were measured using standard enzymatic automated methods. Low-density lipoprotein cholesterol was estimated by the Friedewald equation when the direct parameter will be not available. Atherogenic index was estimated (total cholesterol / HDL cholesterol). Blood samples were collected in the Health Center and analyzed at the hospital of reference.

The researchers who performed the different tests were blinded to the clinical data of the subjects. All assessments were made within a period of 10 days.

Data analysis

Continuous variables were expressed as the mean ± standard deviation. Frequency distributions were used for categorical data. Comparisons between quantitative variables were performed using the student t-test, while the chi-squared test and Fisher's exact test were used for qualitative variables. Pearson's correlation coefficient was used to estimate the relationship of the adiposity measures with CAVI and baPWV.

Four different multiple linear regression models were used to analyze the associations of each adiposity measure with CAVI, and another four models were used with baPWV. CAVI and baPWV were the dependent variables, and the adiposity measures as independent variables in each model. All models were adjusted for age (years), gender (0 = male and 1 = female), systolic blood pressure (SBP), smoking status (0 = No and 1 = Yes), atherogenic index, and HbA1c. The explanatory capacity of the model was measured by R^2, and the proportion attributed to each variable was estimated by the change in R^2. The analysis was also performed by age groups, diagnosis of diabetes, hypertension, and obesity.

ANCOVA models were used to test the differences in the mean values of CAVI and baPWV with the quartiles of the four measures of adiposity after adjusting for the confounding variables used in the regression analysis. Pairwise post hoc comparisons were examined using the Bonferroni test. Data were analyzed using SPSS Statistics for Windows version 23.0

For peer review only - http://bmjopen.bmj.com/site/about/guidelines.xhtml

(IBM Corp, Armonk, NY). Values of $p<0.05$ were considered statistically significant. Continuous variables were expressed as the mean ± standard deviation. Frequency distributions were used for categorical data. Comparisons between quantitative variables were performed using the student t-test, while the chi-squared test and Fisher's exact test were used for qualitative variables. Pearson's correlation coefficient was used to estimate the relationship of the adiposity measures with CAVI and baPWV.

Four different multiple linear regression models were used to analyze the associations of each adiposity measure with CAVI, and another four models were used with baPWV. CAVI and baPWV were the dependent variables, and the adiposity measures as independent variables in each model. All models were adjusted for age (years), gender (0 = male and 1 = female), systolic blood pressure (SBP), smoking status (0 = No and 1 = Yes), atherogenic index, and HbA1c. The explanatory capacity of the model was measured by R^2, and the proportion attributed to each variable was estimated by the change in R^2. The analysis was also performed by age groups, diagnosis of diabetes, hypertension, and obesity.

ANCOVA models were used to test the differences in the mean values of CAVI and baPWV with the quartiles of the four measures of adiposity after adjusting for the confounding variables used in the regression analysis. Pairwise post hoc comparisons were examined using the Bonferroni test. Data were analyzed using SPSS Statistics for Windows version 23.0 (IBM Corp, Armonk, NY). Values of $p<0.05$ were considered statistically significant.

Ethics statement

All participants were informed of the objectives and procedures of the study and signed the informed consent form to participate. The study was approved by the Clinical Research Ethics Committee of the Primary Care Research Institute Jordi Gol, the Health Care Area of Salamanca and Palma of Mallorca. The study was conducted following the recommendations of the Declaration of Helsinki [28]. The confidentiality of information provided by participants

For peer review only - http://bmjopen.bmj.com/site/about/guidelines.xhtml

was ensured, complying with the rules established by Spanish Organic Law 15/1999, of 13 December on the Protection of Personal Data.

RESULTS

The anthropometric measures, clinical characteristics, and vascular function measures of the subjects are presented in Table 1. The mean age of the patients was 61.4±7.7 years, and 61.9% were male. Male subjects had a higher percentage of smokers (31.5 *vs.* 22.7) and hypertension (80.1 *vs.* 75.4). In contrast, females had a higher prevalence of obesity (40.4 *vs.* 33.4), dyslipidemia (73.1 *vs.* 63.6), and diabetes (36.5 *vs.* 31.8). The mean value of CAVI was 8.8±1.2 (8.9 in males and 8.6 in females) and the mean baPWV was 14.9±2.5 (14.8 in males and 15.0 in females). All of the analyzed adiposity measures except for waist circumference presented higher values in women.

Pearson's correlation coefficients between the adiposity measures and the vascular function parameters are shown in table 2. All adiposity measures were negatively correlated with CAVI, and this correlation increases after adjusting for age, sex, and SBP.

Fig. 2 shows the estimated marginal means of CAVI (A) and baPWV (B) by quartiles of the different adiposity measures. After adjustment for the variables used in the multiple linear regression analysis, the mean CAVI values decrease as the quartiles of the four adiposity measurements increased.

In the multiple linear regression analysis, CAVI and baPWV show negative associations with all adiposity measures ($p<0.01$ in all) after adjustment for age, gender, systolic blood pressure, smoking, atherogenic index, and HbA1c (Table 3). The proportions of CAVI variability explained in the respective multiple linear regression analyses by the adiposity measures were 7.5% for BMI, 7.2% for CUN-BAE, 4.3% for WHtR, and 4.1% for BRI. In the case of baPWV, the variance explained by the measures of adiposity was 0.8% BMI, 0.7% for CUN-BAE, and 0.1% for WHtR and BRI. (Table 3).

For peer review only - http://bmjopen.bmj.com/site/about/guidelines.xhtml

In subgroup analysis, the proportion of CAVI variability explained by adiposity measures was higher among diabetics, the obese, non-hypertensive subjects, and subjects 62 years or younger (Table 1S) (supplementary material).

DISCUSSION

The results of this study show that adiposity measures have a negative association with arterial stiffness, especially CAVI. BMI and CUN-BAE have greater explanatory power of the variability of CAVI and baPWV than the WHtR and the BRI, and the explanatory capacity was higher in the case of CAVI. We found a negative association of different adiposity measures with CAVI and baPWV after adjustment for other cardiovascular risk factors.

This negative association with CAVI has already been described in previous studies. BMI has shown a negative association in work performed on children [39], as well as in hypertensive and type 2 diabetes patients in Ghana [40]. Similarly, waist circumference has shown a negative relation in subjects with metabolic syndrome [41; 42].

However, other authors have described a positive association of different adiposity measures with the β-stiffness parameter, but after adjustment for age and other possible confounding factors, the association remained for only men with type 2 diabetes mellitus [43]. On the other hand, other studies have also found no association between BMI and CAVI [44].

Studies analyzing the association of adiposity measures with baPWV have been performed in mainly Eastern populations and have focused on assessing the association of BMI and waist circumference as measures of adiposity. The results have also not been concordant, with some finding a negative association with BMI Studies analyzing the association of adiposity measures with baPWV have been performed in mainly Eastern populations and have focused on assessing the association of BMI and waist circumference as measures of adiposity. The results have also not been concordant, with some finding a negative association with BMI [45],

For peer review only - http://bmjopen.bmj.com/site/about/guidelines.xhtml

and with waist circumference in men only or in women only [46], [47]. However, other studies have found a positive correlation with BMI and waist circumference [48]. One study that analyzed the association of different adiposity parameters with baPWV in middle-aged adults found a positive association with waist circumference and visceral fat but not with body fat percentage [49].

The results are also not consistent in works that have used carotid-femoral pulse wave velocity (cfPWV) as a measure of stiffness. Some studies have described a greater association with measures of central or visceral adiposity in both the general population and in diabetics [10; 13; 17; 18; 19; 21]. However, Strasser et al. [49] found no association with body fat percentage, and other studies also found no association between BMI and cfPWV [13; 14]. Rodrigues et al [15], reported that BMI was negatively associated with cfPWV (β = -0.103) in a large population sample.

Arterial stiffness depends on the elasticity of the arterial wall and the diameter of the arterial wall, and a positive correlation has been found between BMI and aortic diameter measured by nuclear magnetic resonance [50]. This could be a factor that explains the negative association between measures of adiposity and arterial stiffness. These discrepancies between studies may be partially explained by different methods of arterial stiffness measurement and the adjustment variables used. The reason may be that CAVI reflects central and peripheral arterial stiffness and is less influenced by blood pressure values at the time of measurement [36; 51; 52]. In contrast, arterial stiffness assessed with baPWV is a measure of peripheral arterial stiffness [37]. Other possible reasons that may influence the observed differences are age, sex, race, prevalent cardiovascular risk, and drugs used for treatment of the different risk factor [12; 45; 47; 48].

The proportion of baPWV variability explained by adiposity measurements in our study was small (between 0.8% for BMI and 0.1% for WHtR and BRI). The results are also lower than

For peer review only - http://bmjopen.bmj.com/site/about/guidelines.xhtml

those published for the general population by Wohlfahrt et al. (5% for WHtR and 3% for BMI) [(10)] but similar to the results reported by Rodrigues et al. (BMI 0.7%) [(15)]. The proportion of CAVI variability explained by adiposity measures was higher than 7% with CUN-BAE and BMI and higher than 4% with WHtR and BRI. In the subgroup analysis, the proportion of CAVI variability explained by adiposity measures was higher in diabetic, obese, younger, and non-hypertensive subjects. Our results indicate the influence of adiposity measurements on CAVI is greater than on baPWV. We have not found previous work that has analyzed this aspect with CAVI. The novel results of this study may have important clinical relevance since they show the associations of both general and abdominal obesity measures with CAVI and baPWV in subjects with intermediate cardiovascular risk. In addition, the results provide information that could be used in new prospective studies and could potentially help to improve cardiovascular risk equations.

The main limitation of our study is its cross-sectional design, which does not allow us to establish causal relations or the direction of influence of adiposity measures in vascular function. Another limitation that must be mentioned is that the population was ethnically homogeneous (all subjects were Caucasians with intermediate cardiovascular risk). Therefore, the extrapolation of our findings may be limited.

Conclusion

In conclusion, the adiposity measures analyzed show a negative association with measures of arterial stiffness. The explanatory capacity of the variability of arterial stiffness is greater with BMI and CUN-BAE and when using CAVI as a measure of stiffness rather than baPWV. These results suggest that measures of general adiposity and body fat percentage better explain the variability of CAVI than measures of abdominal and visceral adiposity.

For peer review only - http://bmjopen.bmj.com/site/about/guidelines.xhtml

For peer review only

Acknowledgments We are grateful to all professionals participating in the MARK study. Lead author for this group: Rafel Ramos, Research Unit, Primary Health Care, Girona, Jordi Gol Institute for Primary Care Research (IDIAP Jordi Gol), Catalonia, Spain, E-mail: rramos.girona.ics@gencat.net. Coordinating Center: Rafel Ramos, Ruth Martí, Dídac Parramon, Anna Ponjoan, Miquel Quesada, Maria Garcia-Gil, Martina Sidera and Lourdes Camós, Research Unit, Primary Health Care, Jordi Gol Institute for Primary Care Research (IDIAP Jordi Gol), C/Maluquer Salvador, 11, 17002-Girona, Catalonia, Spain. Fernando Montesinos, Ignacio Montoya, Carlos López, Anna Agell, Núria Pagès of the Primary Care Services, Girona, Catalan Institute of Health (ICS), Catalonia, Spain. Irina Gil, Anna Maria-Castro of the Primary Care Services, Girona, Institut d'Assistència Sanitaria (IAS), Catalonia, Spain. Fernando Rigo, Guillermo Frontera, Antònia Rotger, Natalia Feuerbach, Susana Pons, Natividad Garcia, John Guillaumet, Micaela Llull and Mercedes Gutierrez of the Health Center Primary Care San Augustín, Ibsalut Balears, Spain. Cristina Agudo-Conde, Leticia Gómez-Sanchez, Carmen Castaño-Sanchez, Carmela Rodriguez-Martín, Benigna Sanchez-Salgado, Angela de Cabo-Laso, Emiliano Rodriguez-Sanchez, Jose Angel Maderuelo-Fernandez, Marta Gómez-Sánchez, Emilio Ramos-Delgado, Carmen Patino-Alonso, Jose I Recio-Rodriguez, Manuel A Gomez-Marcos and Luis Garcia-Ortiz, Primary Care Research Unit of The Alamedilla, Salamanca, Spain, Castilla and León Health Service–SACYL.

Contributors LG-S designed the study, wrote the protocol, participated in fundraising, interpreted the results, prepared the manuscript draft, performed all analytical testing, interpreted the results and reviewed the manuscript and corrected the final version of the manuscript. JIR-R, RM, FR and CA-C participated in the study design, data collection and manuscript review. ER-S and JAM-F participated in the study design, interpretation of results and manuscript review. MCP-A participated in the analysis of results and final review of the manuscript. LG-O, RR AND MAG-M participated in the protocol design, fundraising,

For peer review only - http://bmjopen.bmj.com/site/about/guidelines.xhtml

analysis of results and final review of the manuscript. All authors reviewed and approved the final version of the manuscript.

Funding This work was supported by grants from the Spanish Ministry of Science and Innovation (MICINN), the Carlos III Health Institute/European Regional Development Fund (ERDF) (MICINN, ISCIII/FEDER) (Red RedIAPP RD06/0018, Research Groups: RD16/0007/0003, RD16/0007/0004, RD16/0007/0008), the Health Research Fund (PI10/01088, PI10/02077, PI10/02043, PI13/01930), and the Regional Health Management of Castile and León (GRS 635/A/11; GRS 906/B/14).

Competing interests None declared.

Ethics approval The Clinical Research Ethics Committee of the Primary Care Research Institute Jordi Gol, the Health Care Area of Salamanca and Palma of Mallorca.

Provenance and peer review Not commissioned; externally peer reviewed.

Data sharing statement No additional data are available.

Informed consent Obtained.

Open Access This is an Open Access article distributed in accordance with the Creative Commons Attribution Non Commercial (CC BY-NC 4.0) license, which permits others to distribute, remix, adapt, build upon this work noncommercially, and license their derivative works on different terms, provided the original work is properly cited and the use is non-commercial. See: http:// creativecommons.org/licenses/by-nc/4.0/

For peer review only

Figure legends

Figure 1: Flow chart of this MARK substudy.

Abbreviations: N, number; CAVI, cardio-ankle vascular index; baPWV, brachial-ankle pulse wave velocity; ABI, ankle-brachial index; WC, waist circumference.

For peer review only

For peer review only - http://bmjopen.bmj.com/site/about/guidelines.xhtml

Figure 2: Data are given as medias estimadas de CAVI y baPWV por cuartiles de BMI, WHtR, CUN-BAE and BRI.

Adjusted for age (years), gender (0=male and 1=female), systolic blood pressure smoking (0=No and 1=Yes), atherogenic index, and HbA1c.

baPWV and CAVI levels were compared using an ANOVA test, followed by post hoc analysis using a Bonferroni test.

CAVI: $p < 0.05$ entre cuartiles, excepto entre el cuartil 2 y el cuartil 3 con el BRI y la WHtR (p= 0.999).

baPWV: $p < 0.05$ entre cuartiles con BMI entre los cuartiles 1 - 4, 2 - 4 y 3-4 and con CUN-BAE entre los cuartiles 1 – 3, 1 – 4 and 2 -4. $P > 0.05$ entre cuartiles de BRI y de WHtR.

Abbreviations: CAVI, cardio-ankle vascular index. ba-PWV, brachial-ankle pulse wave velocity. BMI, body mass index. WHtR, waist-to-height ratio. CUN-BAE, University clinic of Navarra - body adiposity estimator. BRI, body roundness index.

For peer review only

For peer review only - http://bmjopen.bmj.com/site/about/guidelines.xhtml

References

1. Adams KF, Schatzkin A, Harris TB *et al.* (2006) Overweight, obesity, and mortality in a large prospective cohort of persons 50 to 71 years old. *N Engl J Med* **355**, 763-778.

2. Poirier P, Giles TD, Bray GA *et al.* (2006) Obesity and cardiovascular disease: pathophysiology, evaluation, and effect of weight loss: an update of the 1997 American Heart Association Scientific Statement on Obesity and Heart Disease from the Obesity Committee of the Council on Nutrition, Physical Activity, and Metabolism. *Circulation* **113**, 898-918.

3. Turin TC, Kita Y, Rumana N *et al.* (2010) Brachial-ankle pulse wave velocity predicts all-cause mortality in the general population: findings from the Takashima study, Japan. *Hypertens Res* **33**, 922-925.

4. Maeda Y, Inoguchi T, Etoh E *et al.* (2014) Brachial-ankle pulse wave velocity predicts all-cause mortality and cardiovascular events in patients with diabetes: the Kyushu Prevention Study of Atherosclerosis. *Diabetes Care* **37**, 2383-2390.

5. Ikura K, Hanai K, Oka S *et al.* (2016) Brachial-ankle pulse wave velocity, but not ankle-brachial index, predicts all-cause mortality in patients with diabetes after lower extremity amputation. *J Diabetes Investig*.

6. Izuhara M, Shioji K, Kadota S *et al.* (2008) Relationship of cardio-ankle vascular index (CAVI) to carotid and coronary arteriosclerosis. *Circ J* **72**, 1762-1767.

7. Okura T, Watanabe S, Kurata M *et al.* (2007) Relationship between cardio-ankle vascular index (CAVI) and carotid atherosclerosis in patients with essential hypertension. *Hypertens Res* **30**, 335-340.

8. Nakamura K, Tomaru T, Yamamura S *et al.* (2008) Cardio-ankle vascular index is a candidate predictor of coronary atherosclerosis. *Circ J* **72**, 598-604.

9. Satoh-Asahara N, Kotani K, Yamakage H *et al.* (2015) Cardio-ankle vascular index predicts for the incidence of cardiovascular events in obese patients: a multicenter prospective cohort study (Japan Obesity and Metabolic Syndrome Study: JOMS). *Atherosclerosis* **242**, 461-468.

10. Wohlfahrt P, Somers VK, Cifkova R *et al.* (2014) Relationship between measures of central and general adiposity with aortic stiffness in the general population. *Atherosclerosis* **235**, 625-631.

11. Wohlfahrt P, Krajcoviechova A, Seidlerova J *et al.* (2013) Lower-extremity arterial stiffness vs. aortic stiffness in the general population. *Hypertens Res* **36**, 718-724.

12. Moh MC, Sum CF, Lam BC *et al.* (2015) Evaluation of body adiposity index as a predictor of aortic stiffness in multi-ethnic Asian population with type 2 diabetes. *Diab Vasc Dis Res* **12**, 111-118.

13. Teoh WL, Price JF, Williamson RM *et al.* (2013) Metabolic parameters associated with arterial stiffness in older adults with Type 2 diabetes: the Edinburgh Type 2 diabetes study. *J Hypertens* **31**, 1010-1017.

14. Hansen TW, Jeppesen J, Rasmussen S *et al.* (2004) Relation between insulin and aortic stiffness: a population-based study. *J Hum Hypertens* **18**, 1-7.

15. Rodrigues SL, Baldo MP, Lani L *et al.* (2012) Body mass index is not independently associated with increased aortic stiffness in a Brazilian population. *Am J Hypertens* **25**, 1064-1069.

16. Huisman HW, Schutte R, Venter HL *et al.* (2015) Low BMI is inversely associated with arterial stiffness in Africans. *Br J Nutr* **113**, 1621-1627.

17. Canepa M, AlGhatrif M, Pestelli G *et al.* (2014) Impact of central obesity on the estimation of carotid-femoral pulse wave velocity. *Am J Hypertens* **27**, 1209-1217.

For peer review only - http://bmjopen.bmj.com/site/about/guidelines.xhtml

18. Scuteri A, Orru M, Morrell CH *et al.* (2012) Associations of large artery structure and function with adiposity: effects of age, gender, and hypertension. The SardiNIA Study. *Atherosclerosis* **221**, 189-197.
19. Johansen NB, Vistisen D, Brunner EJ *et al.* (2012) Determinants of aortic stiffness: 16-year follow-up of the Whitehall II study. *PLoS One* **7**, e37165.
20. Wildman RP, Mackey RH, Bostom A *et al.* (2003) Measures of obesity are associated with vascular stiffness in young and older adults. *Hypertension* **42**, 468-473.
21. Recio-Rodriguez JI, Gomez-Marcos MA, Patino-Alonso MC *et al.* (2012) Abdominal obesity vs general obesity for identifying arterial stiffness, subclinical atherosclerosis and wave reflection in healthy, diabetics and hypertensive. *BMC Cardiovasc Disord* **12**, 3.
22. Brunner EJ, Shipley MJ, Ahmadi-Abhari S *et al.* (2015) Adiposity, obesity, and arterial aging: longitudinal study of aortic stiffness in the Whitehall II cohort. *Hypertension* **66**, 294-300.
23. Marrugat J, Vila J, Baena-Diez JM *et al.* (2011) [Relative validity of the 10-year cardiovascular risk estimate in a population cohort of the REGICOR study]. *Rev Esp Cardiol* **64**, 385-394.
24. Marti R, Parramon D, Garcia-Ortiz L *et al.* (2011) Improving interMediAte risk management. MARK study. *BMC Cardiovasc Disord* **11**, 61.
25. Marrugat J, D'Agostino R, Sullivan L *et al.* (2003) An adaptation of the Framingham coronary heart disease risk function to European Mediterranean areas. *J Epidemiol Community Health* **57**, 634-638.
26. Conroy RM, Pyorala K, Fitzgerald AP *et al.* (2003) Estimation of ten-year risk of fatal cardiovascular disease in Europe: the SCORE project. *Eur Heart J* **24**, 987-1003.
27. Mancia G, Fagard R, Narkiewicz K *et al.* (2013) 2013 ESH/ESC Guidelines for the management of arterial hypertension: the Task Force for the management of arterial hypertension of the European Society of Hypertension (ESH) and of the European Society of Cardiology (ESC). *J Hypertens* **31**, 1281-1357.
28. (2013) World Medical Association Declaration of Helsinki: ethical principles for medical research involving human subjects. *JAMA* **310**, 2191-2194.
29. Salas-Salvado J, Rubio MA, Barbany M *et al.* (2007) [SEEDO 2007 Consensus for the evaluation of overweight and obesity and the establishment of therapeutic intervention criteria]. *Med Clin (Barc)* **128**, 184-196. .
30. Browning LM, Hsieh SD, Ashwell M (2010) A systematic review of waist-to-height ratio as a screening tool for the prediction of cardiovascular disease and diabetes: 0.5 could be a suitable global boundary value. *Nutr Res Rev* **23**, 247-269.
31. Oliveros E, Somers VK, Sochor O *et al.* (2014) The concept of normal weight obesity. *Prog Cardiovasc Dis* **56**, 426-433.
32. Gomez-Ambrosi J, Silva C, Catalan V *et al.* (2012) Clinical usefulness of a new equation for estimating body fat. *Diabetes Care* **35**, 383-388.
33. Thomas DM, Bredlau C, Bosy-Westphal A *et al.* (2013) Relationships between body roundness with body fat and visceral adipose tissue emerging from a new geometrical model. *Obesity (Silver Spring)* **21**, 2264-2271.
34. Satoh N, Shimatsu A, Kato Y *et al.* (2008) Evaluation of the cardio-ankle vascular index, a new indicator of arterial stiffness independent of blood pressure, in obesity and metabolic syndrome. *Hypertens Res* **31**, 1921-1930.
35. Shirai K, Utino J, Otsuka K *et al.* (2006) A novel blood pressure-independent arterial wall stiffness parameter; cardio-ankle vascular index (CAVI). *J Atheroscler Thromb* **13**, 101-107.
36. Shirai K, Hiruta N, Song M *et al.* (2011) Cardio-ankle vascular index (CAVI) as a novel indicator of arterial stiffness: theory, evidence and perspectives. *J Atheroscler Thromb* **18**, 924-938.

For peer review only - http://bmjopen.bmj.com/site/about/guidelines.xhtml

37. Yamashina A, Tomiyama H, Takeda K *et al.* (2002) Validity, reproducibility, and clinical significance of noninvasive brachial-ankle pulse wave velocity measurement. *Hypertens Res* **25**, 359-364.
38. O'Brien E, Asmar R, Beilin L *et al.* (2005) Practice guidelines of the European Society of Hypertension for clinic, ambulatory and self blood pressure measurement. *J Hypertens* **23**, 697-701.
39. Philip R, Alpert BS, Schwingshackl A *et al.* (2015) Inverse Relationship between Cardio-Ankle Vascular Index and Body Mass Index in Healthy Children. *J Pediatr* **167**, 361-365 e361.
40. Yeboah K, Antwi DA, Gyan B *et al.* (2016) Arterial stiffness in hypertensive and type 2 diabetes patients in Ghana: comparison of the cardio-ankle vascular index and central aortic techniques. *BMC Endocr Disord* **16**, 53.
41. Al-Daghri NM, Al-Attas OS, Wani K *et al.* (2015) Sensitivity of various adiposity indices in identifying cardiometabolic diseases in Arab adults. *Cardiovasc Diabetol* **14**, 101.
42. Gomez-Sanchez L, Garcia-Ortiz L, Patino-Alonso MC *et al.* (2016) Association of metabolic syndrome and its components with arterial stiffness in Caucasian subjects of the MARK study: a cross-sectional trial. *Cardiovasc Diabetol* **15**, 148.
43. Morigami H, Morioka T, Yamazaki Y *et al.* (2016) Visceral Adiposity is Preferentially Associated with Vascular Stiffness Rather than Thickness in Men with Type 2 Diabetes. *J Atheroscler Thromb* **23**, 1067-1079.
44. Kawada T, Andou T, Fukumitsu M (2014) Relationship between cardio-ankle vascular index and components of metabolic syndrome in combination with sex and age. *Diabetes Metab Syndr* **8**, 242-244.
45. Tomiyama H, Yamashina A, Arai T *et al.* (2003) Influences of age and gender on results of noninvasive brachial-ankle pulse wave velocity measurement--a survey of 12517 subjects. *Atherosclerosis* **166**, 303-309.
46. Zhou F, Zhang H, Yao W *et al.* (2014) Relationship between brachial-ankle pulse wave velocity and metabolic syndrome components in a Chinese population. *J Biomed Res* **28**, 262-268.
47. Kim HL, Lee JM, Seo JB *et al.* (2015) The effects of metabolic syndrome and its components on arterial stiffness in relation to gender. *J Cardiol* **65**, 243-249.
48. Weng C, Yuan H, Yang K *et al.* (2013) Gender-specific association between the metabolic syndrome and arterial stiffness in 8,300 subjects. *Am J Med Sci* **346**, 289-294.
49. Strasser B, Arvandi M, Pasha EP *et al.* (2015) Abdominal obesity is associated with arterial stiffness in middle-aged adults. *Nutr Metab Cardiovasc Dis* **25**, 495-502.
50. Danias PG, Tritos NA, Stuber M *et al.* (2003) Comparison of aortic elasticity determined by cardiovascular magnetic resonance imaging in obese versus lean adults. *Am J Cardiol* **91**, 195-199.
51. Takaki A, Ogawa H, Wakeyama T *et al.* (2008) Cardio-ankle vascular index is superior to brachial-ankle pulse wave velocity as an index of arterial stiffness. *Hypertens Res* **31**, 1347-1355.
52. Shirai K (2011) Analysis of vascular function using the cardio-ankle vascular index (CAVI). *Hypertens Res* **34**, 684-685.

For peer review only

Table 1 General characteristics of all the sample and by gender.

Variables	Global (n=2354)	Males (n=1456)	Females (n=898)	p Value
Age (years)	61.4±7.7	61.1±8.1	61.8±7.0	0.030
Smoking n (%)	658 (28.0)	456 (31.5)	202 (22.7)	<0.001
Alcohol (gr/week)	72.2±117.5	102.2±133.3	23.6±59.9	<0.001
Height (cm)	165±9	170±7	156±6	<0.001
Weight (kg)	79.4±14.6	83.9±13.4	72.2±13.3	<0.001
BMI (kg/m^2)	29.2±4.4	29.1±3.9	29.5±5.1	0.035
BMI ≥ 30 n (%)	847 (36.0)	485 (33.4)	362 (40.4)	0.001
Waist circumference (cm)	100.9±11.6	102.9±10.5	97.6±12.5	<0.001
WHtR	0.61±0.07	0.61±0.06	0.62±0.08	<0.001
CUN-BAE	35.7±1.7	31.1±4.5	43.1±5.1	<0.001
BRI	5.8±1.7	5.7±1.5	6.1±2.1	<0.001
SBP (mmHg)	137.1±17.4	138.9±17.1	134.2±17.5	<0.001
DBP (mmHg)	84.4±10.2	85.5±10.4	82.7±9.7	<0.001
Heart rate (beats minutes)	74.2±10.2	73.3±12.7	75.8±11.6	<0.001
Hypertension n (%)	1712 (72.7)	1122 (80.1)	590 (75.4)	<0.001
Antihypertensive drugs n (%)	1199 (50.9)	729 (50.2)	470 (52.6)	0.289
Total Cholesterol (mg/dl)	225.8±40.9	220.8±39.1	233.9±42.5	<0.001
LDL Cholesterol (mg/dl)	140.4±34.9	138.9±34.2	142.8±35.8	0.011
HDL Cholesterol (mg/dl)	49.8±12.9	47.9±11.9	52.9±13.8	<0.001
Triglycerides (mg/dl)	145.5±96.6	150.3±106.3	137.7±77.9	0.001
Atherogenic index	4.8±1.3	4.8±1.3	4.7±1.3	0.002
Dyslipidemia n (%)	1585 (67.3)	927 (63.6)	658 (73.1)	<0.001
Lipid lowering drugs n (%)	671 (28.5)	392 (26.8)	279 (31.0)	0.034
FPG (mg/dl)	107.2±34.8	106.9±33.9	107.6±36.1	0.659
HbA1c	4.8±1.3	5.9±1.4	6.1±1.4	0.001
Diabetes n (%)	791 (33.6)	463 (31.8)	328 (36.5)	0.020
Antidiabetic drugs n (%)	474 (20.1)	269 (18.5)	205 (22.9)	0.011
CAVI	8.8±1.2	8.9±1.2	8.6±1.1	<0.001
baPWV (m/s)	14.9±2.5	14.8±2.5	15.0±2.6	0.107

Values are means and (standard deviations) for continuous data and number and (proportions) for categorical data.

Abbreviations: BMI, body mass index. WHtR, waist-to-height ratio. CUN-BAE, University clinic of Navarra - body adiposity estimator. BRI, body roundness index. SBP, systolic blood pressure. DBP, diastolic blood pressure. LDL, low density lipoprotein. HDL, high density lipoprotein. FPG, fasting plasma glucose. HbA1c, glycosylated hemoglobin. CAVI, cardio ankle vascular index. baPWV, braquial-ankle pulse wave velocity.

p value differences between male and females

For peer review only

Table 2: Bivariate correlations of adiposity measures with CAVI and baPWV.

	CAVI		baPWV	
	Unadjusted	Adjusted [a]	Unadjusted	Adjusted [a]
**BMI**	-0.264**	-0.303**	-0.035	-0.068**
**WHtR**	-0.119**	-0.222**	0.090**	0.001
**CUN-BAE**	-0.187**	-0.297**	0.054*	-0.063**
**BRI**	-0.125**	-0.218**	0.078**	0.005

Abbreviations: CAVI, cardio-ankle vascular index. baPWV, brachial-ankle pulse wave velocity. BMI, body mass index. WHtR, waist-to-height ratio. CUN-BAE, University clinic of Navarra - body adiposity estimator. BRI, body roundness index.

[a] Adjusted for age, sex and systolic blood pressure.

p-values by Pearson correlation.* p<0.05, ** p< 0.01.

For peer review only

Table 3: Multiple regression analysis: association between adiposity measures with CAVI and baPWV.

	R^2	β(95%CI)	Partial R^2	p value
		**CAVI**		
**BMI**	0.410	-0.075 (-0.084 to -0.066)	0.075	<0.001
**WHtR**	0.378	-3.580 (-4.137 to -3.022)	0.043	<0.001
**CUN-BAE**	0.407	-0.067 (-0.075 to -0.060)	0.072	<0.001
**BRI**	0.376	-0.142 (-0.165 to -0.119)	0.041	<0.001
		**baPWV**		
**BMI**	0.398	-0.055 (-0.073 to -0.036)	0.008	<0.001
**WHtR**	0.391	-1.402 (-2.594 to -0.211)	0.001	0.021
**CUN-BAE**	0.397	-0.056 (-0.073 to -0.039)	0.007	<0.001
**BRI**	0.391	-0.060 (-0.109 to -0.012)	0.001	0.014

Four different multiple linear regression models were used to analyze the associations of adiposity measures with CAVI and baPWV.

Adjusted for age (years), gender (0=male and 1=female), systolic blood pressure smoking (0=No and 1=Yes), atherogenic index, and HbA1c.

Abbreviations: CAVI, cardio-ankle vascular index. baPWV, brachial-ankle pulse wave velocity. BMI, body mass index. WHtR, waist-to-height ratio. CUN-BAE, University clinic of Navarra - body adiposity estimator. BRI, body roundness index.

For peer review only

Table 4: Association between adiposity measures with CAVI in different groups.

	R^2	β(95%CI)	Partial R^2	p value
		**CAVI**		
Hypertensive				
**BMI**	0.408	-0.075 (-0.086 to -0.064)	0.068	<0.001
**WHtR**	0.383	-3.816 (-4.512 to -3.120)	0.043	<0.001
**CUN-BAE**	0.406	-0.068 (-0.078 to -0.058)	0.066	<0.001
**BRI**	0.383	-0.157 (-0.186 to -0.129)	0.043	<0.001
Non hypertensive				
**BMI**	0.376	-0.073 (-0.088 to -0.058)	0.095	<0.001
**WHtR**	0.326	-2.999 (-3.915 to -2.083)	0.056	<0.001
**CUN-BAE**	0.407	-0.068 (-0.082 to -0.054)	0.091	<0.001
**BRI**	0.321	-0.110 (-0.147 to -0.074)	0.050	<0.001
Diabetics				
**BMI**	0.434	-0.088 (-0.102 to -0.076)	0.102	<0.001
**WHtR**	0.395	-4.478 (-5.411 to -3.545)	0.039	<0.001
**CUN-BAE**	0.436	-0.084 (-0.097 to -0.070)	0.104	<0.001
**BRI**	0.387	-0.154 (-0.200 to -0.127)	0.047	<0.001
Non diabetics				
**BMI**	0.392	-0.067 (-0.078 to -0.056)	0.054	<0.001
**WHtR**	0.365	-3.057 (-3.764 to -2.350)	0.027	<0.001
**CUN-BAE**	0.393	-0.060 (-0.070 to -0.050)	0.055	<0.001
**BRI**	0.365	-0.128 (-0.158 to -0.099)	0.027	<0.001
Obese				
**BMI**	0.414	-0.090 (-0.109 to -0.071)	0.057	<0.001
**WHtR**	0.376	-2.985 (-4.512 to -3.120)	0.036	<0.001
**CUN-BAE**	0.413	-0.098 (-0.120 to -0.077)	0.058	<0.001
**BRI**	0.376	-0.112 (-4.125 to -1.846)	0.036	<0.001
Non obese				
**BMI**	0.371	-0.063 (-0.083 to - 0.043)	0.016	<0.001
**WHtR**	0.358	-1.397 (-2.371 to - 0.424)	0.002	0.005
**CUN-BAE**	0.370	-0.050 (-0.067 to -0.034)	0.015	<0.001
**BRI**	0.357	0.048 (-0.089 to -0.007)	0.001	0.021
≤ 62 years				
**BMI**	0.358	-0.077 (-0.087 to -0.066)	0.107	<0.001
**WHtR**	0.321	-4.017 (-4.731 to -3.302)	0.070	<0.001
**CUN-BAE**	0.352	-0.067 (-0.077 to -0.058)	0.101	<0.001
**BRI**	0.318	-0.158 (-0.059 to -0.092)	0.067	<0.001
> 62 years				
**BMI**	0.231	-0.074 (-0.088 to -0.060)	0.074	<0.001
**WHtR**	0.191	-3.108 (-3.9980 to -2.235)	0.034	<0.001
**CUN-BAE**	0.230	-0.071 (-0.085 to -0.058)	0.073	<0.001
**BRI**	0.190	0.125 (-0.161 to -0.089)	0.033	<0.001

Multiple linear regression models were used to analyze the associations of adiposity measures with CAVI by groups.

Adjusted for age (years), gender (0=male and 1=female), Systolic blood pressure, smoking (0=No and 1=Yes), atherogenic index, and HbA1c.

Abbreviations: CAVI, cardio-ankle vascular index. BMI, body mass index. WHtR, waist-to-height ratio. CUN-BAE, University clinic of Navarra - body adiposity estimator. BRI, body roundness index.

For peer review only

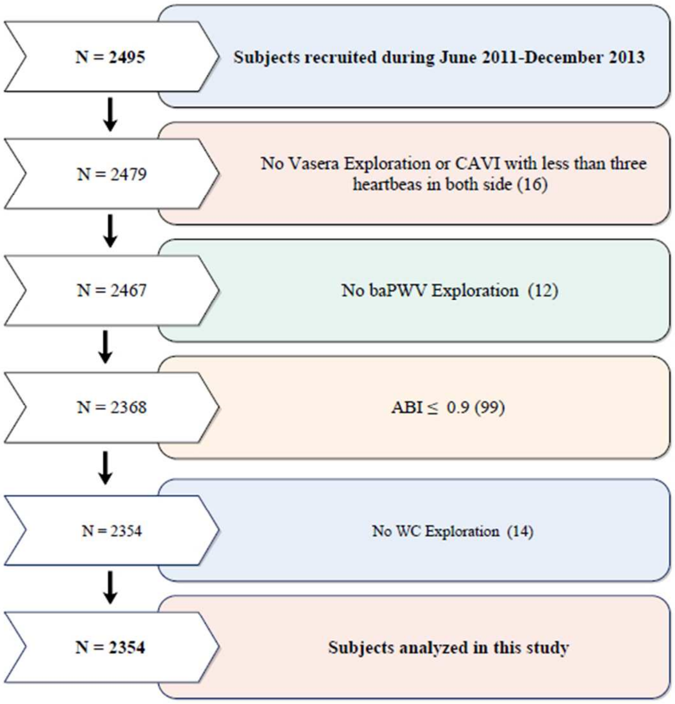

216x159mm (96 x 96 DPI)

For peer review only - http://bmjopen.bmj.com/site/about/guidelines.xhtml

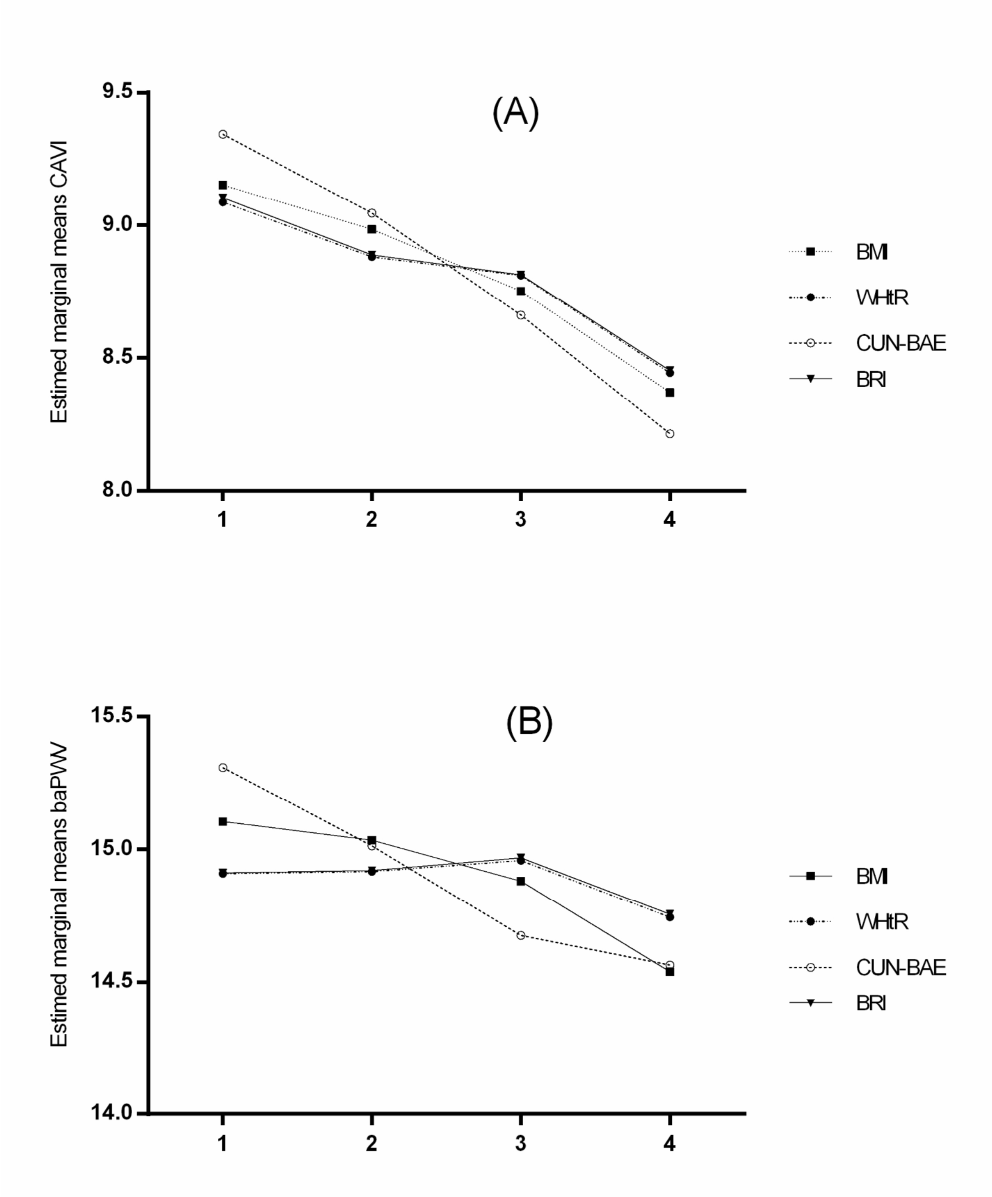

195x236mm (300 x 300 DPI)

STROBE Statement—checklist of items that should be included in reports of observational studies

	Item No	Recommendation
**Title and abstract**	1	(*a*) Indicate the study's design with a commonly used term in the title or the abstract **Yes, Page 1**
		(*b*) Provide in the abstract an informative and balanced summary of what was done and what was found **Yes, Page 2**
**Introduction**		
Background/rationale	2	Explain the scientific background and rationale for the investigation being reported **Yes, Page 4**
Objectives	3	State specific objectives, including any prespecified hypotheses **Yes, Page 4**
**Methods**		
Study design	4	Present key elements of study design early in the paper **Yes, Page 5**
Setting	5	Describe the setting, locations, and relevant dates, including periods of recruitment, exposure, follow-up, and data collection **Yes, Page 5**
Participants	6	(*a*) *Cohort study*—Give the eligibility criteria, and the sources and methods of selection of participants. Describe methods of follow-up *Case-control study*—Give the eligibility criteria, and the sources and methods of case ascertainment and control selection. Give the rationale for the choice of cases and controls *Cross-sectional study*—Give the eligibility criteria, and the sources and methods of selection of participants **Yes, Page 5**
		(*b*) *Cohort study*—For matched studies, give matching criteria and number of exposed and unexposed *Case-control study*—For matched studies, give matching criteria and the number of controls per case **Yes, Page 5**
Variables	7	Clearly define all outcomes, exposures, predictors, potential confounders, and effect modifiers. Give diagnostic criteria, if applicable **Yes, Page 6**
Data sources/ measurement	8*	For each variable of interest, give sources of data and details of methods of assessment (measurement). Describe comparability of assessment methods if there is more than one group **Yes, Page 6-9**
Bias	9	Describe any efforts to address potential sources of bias **Yes, Page 8-9**
Study size	10	Explain how the study size was arrived at **Yes, Page 5**
Quantitative variables	11	Explain how quantitative variables were handled in the analyses. If applicable, describe which groupings were chosen and why **Yes, Page 8-9**

For peer review only - http://bmjopen.bmj.com/site/about/guidelines.xhtml

Statistical methods	12	(*a*) Describe all statistical methods, including those used to control for confounding **Yes, Page 8-9**
		(*b*) Describe any methods used to examine subgroups and interactions **Yes, Page 8-9**
		(*c*) Explain how missing data were addressed **Yes, Page 8-9**
		(*d*) *Cohort study*—If applicable, explain how loss to follow-up was addressed<br>*Case-control study*—If applicable, explain how matching of cases and controls was addressed<br>*Cross-sectional study*—If applicable, describe analytical methods taking account of sampling strategy
		(*e*) Describe any sensitivity analyses **Yes, Page 8-9**

Continued on next page

For peer review only

For peer review only - http://bmjopen.bmj.com/site/about/guidelines.xhtml

**Results**		
Participants	13*	(a) Report numbers of individuals at each stage of study—eg numbers potentially eligible, examined for eligibility, confirmed eligible, included in the study, completing follow-up, and analysed **Yes, Page 10-11**
		(b) Give reasons for non-participation at each stage **Yes, Page 10-11**
		(c) Consider use of a flow diagram **Yes, Figure 1**
Descriptive data	14*	(a) Give characteristics of study participants (eg demographic, clinical, social) and information on exposures and potential confounders **Yes, Page 10**
		(b) Indicate number of participants with missing data for each variable of interest **Yes, Page 10**
		(c) *Cohort study*—Summarise follow-up time (eg, average and total amount)
Outcome data	15*	*Cohort study*—Report numbers of outcome events or summary measures over time
		Case-control study—Report numbers in each exposure category, or summary measures of exposure
		Cross-sectional study—Report numbers of outcome events or summary measures
Main results	16	(*a*) Give unadjusted estimates and, if applicable, confounder-adjusted estimates and their precision (eg, 95% confidence interval). Make clear which confounders were adjusted for and why they were included **Yes, Tables**
		(*b*) Report category boundaries when continuous variables were categorized **Yes, tables**
		(*c*) If relevant, consider translating estimates of relative risk into absolute risk for a meaningful time period
Other analyses	17	Report other analyses done—eg analyses of subgroups and interactions, and sensitivity analyses **Yes, Page 10**
**Discussion**		
Key results	18	Summarise key results with reference to study objectives **Yes, Page 11**
Limitations	19	Discuss limitations of the study, taking into account sources of potential bias or imprecision. Discuss both direction and magnitude of any potential bias **Yes, Page 11**
Interpretation	20	Give a cautious overall interpretation of results considering objectives, limitations, multiplicity of analyses, results from similar studies, and other relevant evidence **Yes, Page 11-13**
Generalisability	21	Discuss the generalisability (external validity) of the study results **Yes, Page 11**
**Other information**		
Funding	22	Give the source of funding and the role of the funders for the present study and, if applicable, for the original study on which the present article is based **Yes, Page 15**

*Give information separately for cases and controls in case-control studies and, if applicable, for exposed and unexposed groups in cohort and cross-sectional studies.

Note: An Explanation and Elaboration article discusses each checklist item and gives methodological background and published examples of transparent reporting. The STROBE checklist is best used in conjunction with this article (freely available on the Web sites of PLoS Medicine at http://www.plosmedicine.org/, Annals of Internal Medicine at http://www.annals.org/, and Epidemiology at http://www.epidem.com/). Information on the STROBE Initiative is available at www.strobe-statement.org.

For peer review only

For peer review only - http://bmjopen.bmj.com/site/about/guidelines.xhtml

BMJ Open

Title: Title: Adiposity measures and arterial stiffness in primary care: the MARK prospective observational study

Journal:	*BMJ Open*
Manuscript ID	bmjopen-2017-016422.R1
Article Type:	Research
Date Submitted by the Author:	27-Apr-2017
Complete List of Authors:	Gomez-Sanchez, Leticia; La Alamedilla health centre., Primary Care Research Unit; 2. Biomedical Research Institute of Salamanca (IBSAL)<br>Garcia-Ortiz, Luis; La Alamedilla health centre. University of Salamanca, Primary care research unit La Alamedilla<br>Patino-Alonso, Maria; University of Salamanca, Salamanca, Spain , Statistics Department,<br>Recio-Rodriguez, Jose; La Alamedilla Health Center, Primary Care Research Unit, The Alamedilla Health Center, Castilla and León Health Service (SACYL), Biomedical Research Institute of Salamanca (IBSAL)<br>Frigo, Fernando; Illes Balears Health Service (IBSALUT)<br>Marti, Rhut; Institut Universitari d'Investigació en Atenció Primària Jordi Gol (IDIAP Jordi Gol), Unitat de Suport a la Recerca de Girona.<br>Agudo-Conde, Cristina; La Alamedilla Health Center,, Research Unit<br>Rodriguez-Sanchez, Emiliano; Primary Care Research Unit, The Alamedilla Health Center, Salamanca, Spain, ; Biomedical Research Institute of Salamanca (IBSAL),<br>Maderuelo-Fernandez, Jose; Salamanca Institute for Biomedical Research. (IBSAL)., Primary care research unit La Alamedilla, Castilla and León Health Service (SACYL). Network of Preventive Activities and Health Promotion in Primary Care (REDIAPP)<br>Ramos, Rafel; Universidad de Girona. Departament Medicina<br>Gomez-Marcos, Manuel; the Alamedilla Health Center, Castilla and León Health Service (SACyL), Primary Care Research Unit; University of Salamanca,, Department of Medicine
**Primary Subject Heading**:	Cardiovascular medicine
Secondary Subject Heading:	Diabetes and endocrinology, Epidemiology, Nutrition and metabolism
Keywords:	Body mass index, Waist circumference, Waist-to-height ratio, Fat mass percent., Body roundness index., Arterial stiffness

SCHOLARONE™ Manuscripts

Title: Adiposity measures and arterial stiffness in primary care: the MARK prospective observational study

Authors: Leticia Gomez-Sanchez MD [1,2]*, Luis Garcia-Ortiz MD, PhD [1,2,3], Maria C Patino-Alonso PhD [1,2,4], Jose I Recio-Rodriguez PhD [1,2,5], Rigo Fernando MD, PhD [6], Ruth Marti PhD [7,8], Cristina Agudo-Conde [1,2], Emiliano Rodriguez-Sanchez MD, PhD [1,2,9], Jose A Maderuelo-Fernandez MD, PhD [1,2], Rafel Ramos MD, PhD [7,8,10] and Manuel A Gomez-Marcos MD, PhD [1,2,9] for the MARK Group [11]

1. Primary Care Research Unit, the Alamedilla Health Center, Castilla and León Health Service (SACyL), Salamanca, Spain
2. Biomedical Research Institute of Salamanca (IBSAL), Salamanca, Spain
3. Biomedical and diagnostic sciences department, University of Salamanca, Salamanca, Spain
4. Statistics Department, University of Salamanca, Salamanca, Spain
5. Department of Nursing and Physiotherapy, University of Salamanca, Salamanca, Spain
6. San Agustín Health Center, Illes Balears Health Service (IBSALUT), Palma of Mallorca, Spain
7. Unitat de Suport a la Recerca de Girona. Institut Universitari d'Investigació en Atenció Primària Jordi Gol (IDIAP Jordi Gol), Girona, Spain
8. Institut d'Investigació Biomèdica of Girona Dr. Josep Trueta (IDBGI). Girona, Spain
9. Department of Medicine, University of Salamanca, Salamanca, Spain
10. Departament of Ciències Mèdiques, Facultat de Medicina. Universitat de Girona, Spain.
11. MARK Group. redIAPP: Research Network in Preventive Activities and Health Promotion, Girona, Spain

***Corresponding Author:**

Leticia Gomez-Sanchez, Primary Care Research Unit, Alamedilla Health Care Center

37003 Salamanca, Spain

E-mail addresses: leticiagmzsnchz@gmail.com

Full list of author information is available at the end of the article.

For peer review only - http://bmjopen.bmj.com/site/about/guidelines.xhtml

ABSTRACT

Background: The cardiovascular risk in obesity is potentially increased by arterial stiffness.

Objective: This study investigates the relationship of adiposity measures with arterial stiffness in Caucasian adults with intermediate cardiovascular risk and determines the best measures to explain arterial stiffness.

Setting: Six primary care centers in three Spanish Autonomous Communities, Spain

Participants: This study analyzed 2354 subjects (age range, 35–74 years; mean age, 61.4±7.7 years, 61.9% male).

Methods: The study was based on cross-sectional data from the MARK study. The main outcome variables were: body mass index (BMI), waist-to-height ratio (WHtR), University clinic of Navarra body adiposity estimation (CUN-BAE) body fat percentage, and body roundness index (BRI). Vascular function was assessed by the cardio-ankle vascular index (CAVI) with the VaSera device, and brachial ankle pulse wave velocity (baPWV) was determined using a validated equation.

Results: The mean adiposity measures were a BMI of 29.2±4.4, WHtR of 0.61±0.07, CUN-BAE of 35.7±1.7, and BRI of 5.8±1.7. The mean stiffness measures were a CAVI of 8.8±1.2 and baPWV of 14.9±2.5. In multiple linear regression analysis, all adiposity measures were negatively associated with CAVI and baPWV ($p<0.01$ for all) after adjustment for cardiovascular risk factors. The proportion of CAVI variability explained by the adiposity measures were 5.5% for BMI, 5.8% for CUN-BAE, 3.8% for WHtR, and 3.7% for BRI, which were higher among diabetic, obese, younger (≤62 years), and non-hypertensive subjects who had similar activity and sedentary profiles.

Conclusions: Adiposity measures are negatively associated with arterial stiffness measures. The arterial stiffness variability is better explained for BMI and CUN-BAE and when CAVI is used as a measure of stiffness rather than baPWV.

For peer review only - http://bmjopen.bmj.com/site/about/guidelines.xhtml

Trial Registration: Clinical Trials.gov Identifier: https://clinicaltrials.gov/ct2/show/NCT01428934. Registered 2 September 2011. Last updated September 8, 2016.

Keywords: body mass index, waist circumference, waist-to-height ratio, fat mass percent, body roundness index, arterial stiffness

Strengths and limitations of this study

- This is the first study to investigate the association between adiposity measures using a cardio-ankle vascular index with and brachial ankle pulse wave velocity in Caucasian adults with intermediate cardiovascular risk.
- All adiposity measures were negatively associated with CAVI and baPWV.
- The arterial stiffness variability was better explained for BMI and CUN-BAE and when CAVI was used as a measure of stiffness rather than baPWV.
- The main limitation of our study is its cross-sectional design, which does not allow establishment of causal relationships or the direction in which adiposity measures influence vascular function.

For peer review only - http://bmjopen.bmj.com/site/about/guidelines.xhtml

INTRODUCTION

Obesity has been linked to increased all-cause and cardiovascular mortality [1]. However, the mechanisms through which obesity can increase the frequency of cardiovascular disease beyond traditional risk factors are not clearly identified [2]. Arterial stiffness as evaluated by the brachial ankle pulse wave velocity (baPWV) is an independent predictor of coronary heart disease and mortality in both the general population [3] and in patients with diabetes mellitus [4; 5]. Similarly, the cardio-ankle vascular index (CAVI) is associated with carotid and coronary atherosclerosis [6; 7; 8] and is a predictor of cardiovascular events in obese patients [9].

Increased arterial stiffness may be a mechanism by which obesity increases cardiovascular risk independently of traditional risk factors. However, the relationship between adiposity and arterial stiffness remains controversial. The body mass index (BMI) has been associated with arterial stiffness in the general population [10; 11] and in diabetic patients [12; 13]. However, other research has not found this association [14], the association disappeared after adjusting for potential confounders [13], or it showed a negative association [15; 16]. Additionally, there are studies that suggest a stronger correlation of measures of central or visceral adiposity than measures of general adiposity with arterial stiffness in the general population [10; 17; 18; 19; 20], in diabetic patients [13], and in diabetics and hypertensive patients [21]. The Whitehall II Cohort study [22] showed that all measures of general adiposity, central adiposity, and body fat percentage were predictors of accelerated arterial stiffness in adults.

Cardiovascular events are more likely to occur in patients with intermediate cardiovascular risk [23], and there is a lack of studies analyzing the relationship of different adiposity measures with arterial stiffness in these subjects. Thus, the primary aim of this study was to investigate the relationship between adiposity measures and arterial stiffness in Caucasian adults with intermediate cardiovascular risk. The secondary aim was to determine which adiposity measures best explain arterial stiffness.

For peer review only - http://bmjopen.bmj.com/site/about/guidelines.xhtml

METHODS

Study design

This trial is a cross-sectional study of subjects recruited to the *improving interMediAte RisK management (MARK)* study (NCT01428934) [24], which is a longitudinal study designed to assess whether the ankle-brachial index, arterial stiffness (measured by CAVI), postprandial glucose, glycosylated hemoglobin, self-measured blood pressure, and the presence of comorbidities are independently associated with the occurrence of vascular events. It also investigates whether the predictive capacity of current risk equations can be improved in the intermediate risk population. The current study focuses on the baseline visit. The second step will be a 5- and 10-year follow-up trial to assess cardiovascular morbidity and mortality.

Study population

In this multicenter project, the study population was obtained by random sampling from subjects who met the inclusion criteria and who were seeing general practitioners from July 2011 to June 2013 at six primary care centers in three Spanish Autonomous Communities. Subjects were recruited from those aged 35 to 74 years with an intermediate cardiovascular risk, defined as a 10-year coronary risk ranging from 5%–15% according to the adapted Framingham risk equation [25]; a 10-year vascular mortality risk ranging from 1%–5% according to the scoring risk in Europeans equation [26]; or a moderate risk according to the European Society of Hypertension guidelines for the management of arterial hypertension [27]. Exclusion criteria included end-stage disease or institutionalization at the time of the visit, or history of atherosclerotic disease. This study analyzed 2354 of the 2495 subjects recruited in the MARK study, and the exclusion criteria are shown in Figure 1.

For peer review only - http://bmjopen.bmj.com/site/about/guidelines.xhtml

Variables and measurement instruments

A detailed description of procedures for clinical data collection and laboratory tests has been published elsewhere [24].

Anthropometric measurements

Body weight was measured twice using a certified electronic scale (Seca 770, Medical scale and measurement systems, Birmingham, United Kingdom) after adequate calibration (precision ± 0.1 kg). Readings were rounded-off to the nearest 100 g. Height was measured using a stadiometer (Seca 222), and the average of two measurements was recorded. Body mass index (BMI) was calculated as weight (kg) divided by height squared (m^2). Waist circumference was measured according to the 2007 recommendations of the Spanish Society for the Study of Obesity [28]. All measurements were performed with the subjects standing, wearing no shoes, and in light clothing. Waist-to-height ratio (WHtR) was calculated as the waist circumference (cm) divided by height (cm) [29; 30].

The body fat percentage (BF%) was calculated according to the University clinic of Navarra - body adiposity estimator (CUN-BAE) using the recommendations of Gomez-Ambrosi et al. [31]: $BF\% = -44.988 + (0.503 \times Age) + (10.689 \times sex) + (3.172 \times BMI) - (0.026 \times BMI^2) + (0.181 \times BMI \times sex) - (0.02\ BMI \times Age) - (0.005 \times BMI^2 \times sex) + (0.00021 \times BMI^2 \times Age)$, where male=0 and female=1 for sex.

Body roundness index (BRI) was calculated using the following formula: $BRI = 364.2 - 365.5 \times \sqrt{1-\frac{(WC/(2\pi))^2}{(0.5height)^2}}$ [32]. BRI can predict the percentage of body fat and visceral adipose tissue.

Cardio-ankle vascular index (CAVI) and brachial-ankle pulse wave velocity (baPWV)

For peer review only

CAVI was measured using a *VaSera VS-1500®* device *(Fukuda Denshi)* [33; 34]. CAVI values are calculated automatically by estimating the stiffness parameter β using the following equation: $\beta = 2\rho \times 1 / (Ps - Pd) \times \ln (Ps / Pd) \times PWV^2$, where ρ is blood density, Ps and Pd are SBP and DBP in mmHg, and PWV is measured between the aortic valve and the ankle [35]. The mean coefficient of variation of CAVI measurement is less than 5%, which is small enough to allow for clinical use of the index and confirms that CAVI has a favorable reproducibility [34].

CAVI was calculated using the VaSera VS-1500® device (Fukuda Denshi), and with the values obtained, the baPWV was estimated using the equation baPWV= (0.5934 × height (cm) + 14.4724)/tba (where tba is the time interval between the arm and ankle waves) [36].

Measurements were performed with the patient in the supine position after resting for 10 minutes in a quiet room at a stable temperature. Subjects were instructed not to smoke or exercise in the hour before the test.

Diagnosis of cardiovascular risk factors

Subjects were considered to be hypertensive if they were previously diagnosed with hypertension, if they were taking antihypertensive drugs, or if they had blood pressure levels ≥ 140/90 mmHg. Diabetic subjects were those who had been previously diagnosed with the disease, were taking hypoglycemic drugs, or had a fasting blood glucose levels ≥ 126 mg/dL or HbA1c ≥ 6.5%. Dyslipidemia was defined if they were treated with lipid-lowering drugs or had altered LDL ≥ 130 mg/dl, HDL ≤ 45 mg/dl in men and ≤ 55 in women, and TG ≥ 150 mg/dl, as established by the European Society of Cardiology (ESC) and the European Atherosclerosis Society (EAS) 2011 [37].

Office or clinical blood pressure

For peer review only - http://bmjopen.bmj.com/site/about/guidelines.xhtml

Office blood pressure measurement involved three measurements of SBP and DBP using a validated OMRON model M10-IT sphygmomanometer (Omron Health Care, Kyoto, Japan). The measurements followed the recommendations of the European Society of Hypertension [38], and the average of the last two measurements was used.

Lifestyles

Tobacco

Smoking history was assessed by asking questions about the participant's smoking status (smoker/non-smoker). We considered smokers to include those who currently smoke or who had stopped smoking within the past year.

Leisure time physical activity

Leisure time physical activity (LTPA) was collected using the Minnesota LTPA Questionnaire [39] that was validated for Spanish men and women[40; 41]. The questionnaire was administered by trained interviewers who spent about 10 to 20 min per participant collecting detailed information about physical activity (PA) during the preceding year, the number of times this activity was performed, and the average duration of each activity on each occasion. Each PA has an intensity code, based on the ratio between the metabolic rate during PA practice and the basal metabolic rate (MET) [42]. We assumed that 1 MET approximately corresponds to 1 kcal/min of energy expenditure. Therefore, we can calculate the total energy expenditure in leisure time of PA ($EEPA_{total}$) in kilocalories per week. Moreover, based on the PA intensity code, we could quantify the energy expenditure in physical activity (EEPA) according to the activity's classification as intense, moderate, or light intensity as follows: light PA intensity is below 4 METs, such as walking ($EEPA_{light}$). Moderate PA intensity is 4–5.5 METs, such as brisk walking ($EEPA_{moderate}$). Intense PA intensity is greater than or equal to 6 METs, such as jogging ($EEPA_{intense}$). Thus, for each particular subject: $EEPA_{total} = EEPA_{light} + EEPA_{moderate} + EEPA_{intense}$.

Based on recommendations from the American Heart Association [43], we considered those participants who do not meet the recommendations of moderate-intensity aerobic PA practice

For peer review only - http://bmjopen.bmj.com/site/about/guidelines.xhtml

for a minimum of 30 min on 5 days each week (EEPA $_{moderate}$ < 675 kcal/week) or high-intensity aerobic PA practice for a minimum of 20 min on 3 days each week (EEPA $_{intense}$ < 420 kcal/week) to be sedentary.

Laboratory determinations

Venous blood sampling was performed between 08:00 and 09:00 hours after the individuals fasted and abstained from smoking and the consumption of alcohol and caffeinated beverages for the previous 12 hours. Fasting plasma glucose, serum, high-density lipoprotein (HDL) cholesterol concentrations and triglyceride concentrations were measured using standard enzymatic automated methods. Low-density lipoprotein cholesterol was estimated using the Friedewald equation when the direct parameter was not available. The atherogenic index was estimated (total cholesterol / HDL cholesterol). Blood samples were collected at the Health Center and analyzed at the hospital of reference.

The researchers who performed the different tests were blinded to the clinical data of the subjects. All assessments were made within a period of 10 days.

Data analysis

Continuous variables were expressed as the mean ± standard deviation. Frequency distributions were used for categorical data. Comparisons between quantitative variables were performed using the Student's *t*-test, while the chi-squared test and Fisher's exact test were used for qualitative variables. Pearson's correlation coefficient was used to estimate the relationship of the adiposity measures to CAVI and baPWV.

Four different multiple linear regression models were used to analyze the associations of each adiposity measure with CAVI, and four other models were used with baPWV. CAVI and baPWV were the dependent variables, and the adiposity measures were the independent variables in each model. All models were adjusted for age (years), sex (0 = male and 1 =

For peer review only - http://bmjopen.bmj.com/site/about/guidelines.xhtml

female), systolic blood pressure (SBP), smoking status (0 = No and 1 = Yes), atherogenic index, HbA1c, and METs/min/week. The explanatory capacity of the model was measured by R^2, and the proportion attributed to each variable was estimated by the change in R^2. The analysis was also performed by age groups, diagnosis of diabetes, hypertension, and obesity.

ANCOVA models were used to test the differences in the mean values of CAVI and baPWV with the quartiles of the four adiposity measures after adjusting for the confounding variables that were used in the regression analysis. Pairwise post hoc comparisons were examined using the Bonferroni test. Data were analyzed using SPSS Statistics for Windows version 23.0 (IBM Corp, Armonk, NY). Values of $p<0.05$ were considered statistically significant.

Ethics statement

All participants were informed of the study objectives and procedures and they all signed an informed consent form to participate. The study was approved by the Clinical Research Ethics Committee of the Primary Care Research Institute Jordi Gol, the Health Care Area of Salamanca, and Palma of Mallorca. The study was conducted following the recommendations of the Declaration of Helsinki [(44)]. The confidentiality of information provided by participants was ensured, complying with the rules established by Spanish Organic Law 15/1999, of 13 December on the Protection of Personal Data.

RESULTS

Anthropometric measures, clinical characteristics, and vascular function measures of the subjects are presented in Table 1. The mean age of the patients was 61.4±7.7 years, and 61.9% were male. Male subjects had a higher percentage of smokers (31.5 *vs.* 22.7) and hypertension (80.1 *vs.* 75.4) compared with females. However, females had a higher

For peer review only - http://bmjopen.bmj.com/site/about/guidelines.xhtml

prevalence of obesity (40.4 *vs.* 33.4), sedentariness (53.7 vs. 37.0), dyslipidemia (73.1 *vs.* 63.6), and diabetes (36.5 *vs.* 31.8) compared with males. The mean value of CAVI was 8.8±1.2 (8.9 in males and 8.6 in females, p<0.001) and the mean baPWV was 14.9±2.5 (14.8 in males and 15.0 in females). All of the analyzed adiposity measures except for waist circumference presented higher values in women compared with men.

Pearson's correlation coefficient results between the adiposity measures and the vascular function parameters are shown in Table 2. All adiposity measures were negatively correlated with CAVI, and this correlation increases after adjusting for age, sex, and SBP. The correlation between CAVI and baPWV was r=0.745 (p<0.001).

Figure 1S (Supplementary material) shows the estimated marginal means of CAVI (a) and baPWV (b) by quartiles of the different adiposity measures. After adjustment for the variables used in the multiple linear regression analysis, the mean CAVI values decreased as the quartiles of the four adiposity measurements increased (p<0.05). However, the same is not true of baPWV with WHtR and BRI (p>0.05).

In the multiple linear regression analysis, CAVI and baPWV showed negative associations with all adiposity measures (p<0.01 for all) after adjustment for age, sex, SBP, smoking, atherogenic index, HbA1c, and METs/min/week (Table 3). The proportion of CAVI variability explained in the respective multiple linear regression analyses by the adiposity measures was 5.5% for BMI, 5.8% for CUN-BAE, 3.8% for WHtR, and 3.7% for BRI. For baPWV, the variance explained by the measures of adiposity were 0.7% for BMI, and CUN-BAE, and 0.1% for WHtR, and 0.2 for BRI.

In the multiple linear regression analysis by subgroup, the proportion of CAVI variability explained by adiposity measures was higher among diabetics, obese, non-hypertensive, and subjects 62 years of age or younger, and was similar in active and sedentary people (Table 4).

For peer review only - http://bmjopen.bmj.com/site/about/guidelines.xhtml

DISCUSSION

The results of this study show that adiposity measures have a negative association with arterial stiffness, especially CAVI. BMI and CUN-BAE better explain the variability of CAVI and baPWV than the WHtR and the BRI, and the explanatory capacity was higher in the case of CAVI. We found a negative association of different adiposity measures with CAVI and baPWV after adjustment for other cardiovascular risk factors.

In this study the mean value of CAVI was higher in males, which is in agreement with published data indicating that CAVI increases linearly with age, and is higher in males than in females (approximately 0.2, which is equivalent to 4−5 years old) [22; 45]. Similarly, there was no difference in the mean baPWV between the sexes, which is consistent with data published by Tomiyama et al. [46] who showed that the effect of age on baPWV is different according to sex. Females have a higher arterial stiffness than prepubertal males, and this increases after menopause. Men, however, experience a linear increase in arterial stiffness from puberty. This suggests that women have large arteries that are intrinsically more rigid compared with men, but these effects are mitigated by sex steroids during the reproductive years [47; 48].

This negative association with CAVI has already been described in previous studies. BMI has shown a negative association in children [49], and in hypertensive and type 2 diabetes patients in Ghana [50]. Similarly, waist circumference has shown a negative relationship in subjects with metabolic syndrome [51; 52].

However, other authors have described a positive association of different adiposity measures with the β-stiffness parameter, but after adjustment for age and other possible confounding factors, the association remained for only men with type 2 diabetes mellitus [53]. Other studies have also found no association between BMI and CAVI [54].

Studies analyzing the association of adiposity measures with baPWV have also been performed mainly in Eastern populations and have focused on assessing the association

For peer review only - http://bmjopen.bmj.com/site/about/guidelines.xhtml

between BMI and waist circumference as measures of adiposity. The results are controversial, with some finding a negative association with BMI [46], and with waist circumference in men only or in women only [55), (56]. However, other studies have found a positive correlation with BMI and waist circumference [57]. One study that analyzed the association of different adiposity parameters with baPWV in middle-aged adults found a positive association with waist circumference and visceral fat but not with body fat percentage [58].

The results are also not consistent in studies that used the carotid-femoral pulse wave velocity (cfPWV) as a measure of stiffness. Some studies have described a greater association with measures of central or visceral adiposity in both the general population and in diabetics [10; 13; 17; 18; 19; 21]. However, Strasser et al. [58] found no association with body fat percentage, and other studies also found no association between BMI and cfPWV [13; 14]. Rodrigues et al. [15], reported that BMI was negatively associated with cfPWV ($\beta = -0.103$) in a large population sample. The Whitehall II Cohort study [22], which was completed based on staff lists from offices located in central London, showed that all measures of adiposity were robust predictors of accelerated CFPWV, after adjusting them for potential confounding factors. The use of different measures to measure arterial stiffness such as CAVI and baPWV, as well as being a population with intermediate cardiovascular risk could explain some of the discrepancies with our study.

Arterial stiffness depends on arterial wall elasticity and diameter, and a positive correlation was found between BMI and aortic diameter measured by nuclear magnetic resonance [59]. This could partially explain the negative association between measures of adiposity and arterial stiffness. These discrepancies between studies may be partially explained by different methods of arterial stiffness measurement and the adjustment variables used. This may be because CAVI reflects central and peripheral arterial stiffness and is less influenced by blood pressure values at the time of measurement [35; 60; 61]. Conversely, arterial stiffness assessed

For peer review only - http://bmjopen.bmj.com/site/about/guidelines.xhtml

using baPWV is a measure of peripheral arterial stiffness [36]. Other potential influences on the observed differences are age, sex, race, prevalent cardiovascular risk, and drugs used for treatment of the different risk factor [12; 46; 56; 57].

The proportion of baPWV variability explained by adiposity measurements in our study was less than 1% (between 0.7% for BMI and 0.1% for CUN-BAE). The results are also lower than those published for the general population by Wohlfahrt et al. (5% for WHtR and 3% for BMI) [10], but similar to the results reported by Rodrigues et al. (BMI 0.7%) [15]. The proportion of CAVI variability that is explained by adiposity measures was higher than 5% with CUN-BAE and BMI and higher than 3.5% with WHtR and BRI. In the subgroup analysis, the proportion of CAVI variability explained by adiposity measures was higher in diabetic, obese, younger, and non-hypertensive subjects. Our results show that the influence of adiposity measurements on CAVI is greater than on baPWV. To our knowledge, no other study has analyzed this aspect using CAVI. The novel results of this study may have important clinical relevance because they show the associations of both general and abdominal obesity measures with CAVI and baPWV in subjects with intermediate cardiovascular risk. Additionally, the results provide information that could be used in new prospective studies and could potentially help to improve cardiovascular risk equations.

In summary, our results showed the correlation between measures of adiposity and measures of arterial stiffness is greater with CAVI than with baPWV. The different measures of adiposity better explain the variability of arterial stiffness evaluated using CAVI than using baPWW. All this suggests that the relationship with adiposity measures is greater if the arterial stiffness is measured using CAVI than using baPWV. This is likely because CAVI measures rigidity at the central and peripheral levels and it is not influenced by blood pressure at the time of measurement [35; 60; 62; 63].

For peer review only - http://bmjopen.bmj.com/site/about/guidelines.xhtml

The main limitation of our study is its cross-sectional design, which does not allow us to establish causal relationships or the direction of influence of adiposity measures in vascular function. Another limitation is that the population was ethnically homogeneous (all subjects were Caucasians with intermediate cardiovascular risk). Therefore, the extrapolation of our findings may be limited.

CONCLUSION

In conclusion, the adiposity measures analyzed show a negative association with arterial stiffness measures. Arterial stiffness is better explained with BMI and CUN-BAE and when using CAVI as a measure of stiffness rather than baPWV. These results suggest that measures of general adiposity and body fat percentage better explain the variability of CAVI compared with measures of abdominal and visceral adiposity.

Acknowledgments: We are grateful to all professionals participating in the MARK study. Lead author for this group: Rafel Ramos, Research Unit, Primary Health Care, Girona, Jordi Gol Institute for Primary Care Research (IDIAP Jordi Gol), Catalonia, Spain, E-mail: rramos.girona.ics@gencat.net. Coordinating Center: Rafel Ramos, Ruth Martí, Dídac Parramon, Anna Ponjoan, Miquel Quesada, Maria Garcia-Gil, Martina Sidera and Lourdes Camós, Research Unit, Primary Health Care, Jordi Gol Institute for Primary Care Research (IDIAP Jordi Gol), C/Maluquer Salvador, 11, 17002-Girona, Catalonia, Spain. Fernando Montesinos, Ignacio Montoya, Carlos López, Anna Agell, Núria Pagès of the Primary Care Services, Girona, Catalan Institute of Health (ICS), Catalonia, Spain. Irina Gil, Anna Maria-Castro of the Primary Care Services, Girona, Institut d'Assistència Sanitaria (IAS), Catalonia, Spain. Fernando Rigo, Guillermo Frontera, Antònia Rotger, Natalia Feuerbach, Susana Pons, Natividad Garcia, John Guillaumet, Micaela Llull and Mercedes Gutierrez of the Health

For peer review only

Center Primary Care San Augustín, Ibsalut Balears, Spain. Cristina Agudo-Conde, Leticia Gómez-Sanchez, Carmen Castaño-Sanchez, Carmela Rodriguez-Martín, Benigna Sanchez-Salgado, Angela de Cabo-Laso, Emiliano Rodriguez-Sanchez, Jose Angel Maderuelo-Fernandez, Marta Gómez-Sánchez, Emilio Ramos-Delgado, Carmen Patino-Alonso, Jose I Recio-Rodriguez, Manuel A Gomez-Marcos and Luis Garcia-Ortiz, Primary Care Research Unit of The Alamedilla, Salamanca, Spain, Castilla and León Health Service–SACYL.

Contributors: LG-S designed the study, wrote the protocol, participated in fundraising, interpreted the results, prepared the manuscript draft, performed all analytical testing, interpreted the results and reviewed the manuscript and corrected the final version of the manuscript. JIR-R, RM, FR, and CA-C participated in the study design, data collection and manuscript review. ER-S and JAM-F participated in the study design, interpretation of results and manuscript review. MCP-A participated in the analysis of results and final review of the manuscript. LG-O, RR, and MAG-M participated in the protocol design, fundraising, analysis of results, and final review of the manuscript. All authors reviewed and approved the final version of the manuscript.

Funding: This research was supported by grants from the Spanish Ministry of Science and Innovation (MICINN), the Carlos III Health Institute/European Regional Development Fund (ERDF) (MICINN, ISCIII/FEDER) (Red RedIAPP RD06/0018, Research Groups: RD16/0007/0003, RD16/0007/0004, RD16/0007/0008), the Health Research Fund (PI10/01088, PI10/02077, PI10/02043, PI13/01930), and the Regional Health Management of Castile and León (GRS 635/A/11; GRS 906/B/14).

Competing interests: None declared.

For peer review only

Ethics approval: The Clinical Research Ethics Committee of the Primary Care Research Institute Jordi Gol, the Health Care Area of Salamanca and Palma of Mallorca.

Provenance and peer review: Not commissioned; externally peer reviewed.

Data sharing statement: No additional data are available.

Informed consent: Obtained.

Open Access: This is an Open Access article distributed in accordance with the Creative Commons Attribution Non Commercial (CC BY-NC 4.0) license, which permits others to distribute, remix, adapt, build upon this work non commercially, and license their derivative works on different terms, provided the original work is properly cited and the use is non-commercial. See: http:// creativecommons.org/licenses/by-nc/4.0/

For peer review only

Figure legends

Figure 1. Flow chart of this MARK substudy

Abbreviations: N, number; CAVI, cardio-ankle vascular index; baPWV, brachial-ankle pulse wave velocity; ABI, ankle-brachial index; WC, waist circumference

For peer review only

For peer review only - http://bmjopen.bmj.com/site/about/guidelines.xhtml

Figure 1S (Supplementary material). Data are given as medias estimadas de CAVI y baPWV por cuartiles de BMI, WHtR, CUN-BAE and BRI.

Adjusted for age (years), sex (0=male and 1=female), systolic blood pressure smoking (0=No and 1=Yes), atherogenic index, HbA1c, and METs/min/week

baPWV and CAVI levels were compared using an ANOVA.

Abbreviations: CAVI, cardio-ankle vascular index; ba-PWV, brachial-ankle pulse wave velocity; BMI, body mass index; WHtR, waist-to-height ratio; CUN-BAE, University clinic of Navarra - body adiposity estimator; BRI, body roundness index

References

1. Adams KF, Schatzkin A, Harris TB *et al.* (2006) Overweight, obesity, and mortality in a large prospective cohort of persons 50 to 71 years old. *N Engl J Med* **355**, 763-778.
2. Poirier P, Giles TD, Bray GA *et al.* (2006) Obesity and cardiovascular disease: pathophysiology, evaluation, and effect of weight loss: an update of the 1997 American Heart Association Scientific Statement on Obesity and Heart Disease from the Obesity Committee of the Council on Nutrition, Physical Activity, and Metabolism. *Circulation* **113**, 898-918.
3. Turin TC, Kita Y, Rumana N *et al.* (2010) Brachial-ankle pulse wave velocity predicts all-cause mortality in the general population: findings from the Takashima study, Japan. *Hypertens Res* **33**, 922-925.
4. Maeda Y, Inoguchi T, Etoh E *et al.* (2014) Brachial-ankle pulse wave velocity predicts all-cause mortality and cardiovascular events in patients with diabetes: the Kyushu Prevention Study of Atherosclerosis. *Diabetes Care* **37**, 2383-2390.
5. Ikura K, Hanai K, Oka S *et al.* (2016) Brachial-ankle pulse wave velocity, but not ankle-brachial index, predicts all-cause mortality in patients with diabetes after lower extremity amputation. *J Diabetes Investig*.
6. Izuhara M, Shioji K, Kadota S *et al.* (2008) Relationship of cardio-ankle vascular index (CAVI) to carotid and coronary arteriosclerosis. *Circ J* **72**, 1762-1767.
7. Okura T, Watanabe S, Kurata M *et al.* (2007) Relationship between cardio-ankle vascular index (CAVI) and carotid atherosclerosis in patients with essential hypertension. *Hypertens Res* **30**, 335-340.
8. Nakamura K, Tomaru T, Yamamura S *et al.* (2008) Cardio-ankle vascular index is a candidate predictor of coronary atherosclerosis. *Circ J* **72**, 598-604.
9. Satoh-Asahara N, Kotani K, Yamakage H *et al.* (2015) Cardio-ankle vascular index predicts for the incidence of cardiovascular events in obese patients: a multicenter prospective cohort study (Japan Obesity and Metabolic Syndrome Study: JOMS). *Atherosclerosis* **242**, 461-468.
10. Wohlfahrt P, Somers VK, Cifkova R *et al.* (2014) Relationship between measures of central and general adiposity with aortic stiffness in the general population. *Atherosclerosis* **235**, 625-631.
11. Wohlfahrt P, Krajcoviechova A, Seidlerova J *et al.* (2013) Lower-extremity arterial stiffness vs. aortic stiffness in the general population. *Hypertens Res* **36**, 718-724.
12. Moh MC, Sum CF, Lam BC *et al.* (2015) Evaluation of body adiposity index as a predictor of aortic stiffness in multi-ethnic Asian population with type 2 diabetes. *Diab Vasc Dis Res* **12**, 111-118.

For peer review only - http://bmjopen.bmj.com/site/about/guidelines.xhtml

13. Teoh WL, Price JF, Williamson RM *et al.* (2013) Metabolic parameters associated with arterial stiffness in older adults with Type 2 diabetes: the Edinburgh Type 2 diabetes study. *J Hypertens* **31**, 1010-1017.
14. Hansen TW, Jeppesen J, Rasmussen S *et al.* (2004) Relation between insulin and aortic stiffness: a population-based study. *J Hum Hypertens* **18**, 1-7.
15. Rodrigues SL, Baldo MP, Lani L *et al.* (2012) Body mass index is not independently associated with increased aortic stiffness in a Brazilian population. *Am J Hypertens* **25**, 1064-1069.
16. Huisman HW, Schutte R, Venter HL *et al.* (2015) Low BMI is inversely associated with arterial stiffness in Africans. *Br J Nutr* **113**, 1621-1627.
17. Canepa M, AlGhatrif M, Pestelli G *et al.* (2014) Impact of central obesity on the estimation of carotid-femoral pulse wave velocity. *Am J Hypertens* **27**, 1209-1217.
18. Scuteri A, Orru M, Morrell CH *et al.* (2012) Associations of large artery structure and function with adiposity: effects of age, gender, and hypertension. The SardiNIA Study. *Atherosclerosis* **221**, 189-197.
19. Johansen NB, Vistisen D, Brunner EJ *et al.* (2012) Determinants of aortic stiffness: 16-year follow-up of the Whitehall II study. *PLoS One* **7**, e37165.
20. Wildman RP, Mackey RH, Bostom A *et al.* (2003) Measures of obesity are associated with vascular stiffness in young and older adults. *Hypertension* **42**, 468-473.
21. Recio-Rodriguez JI, Gomez-Marcos MA, Patino-Alonso MC *et al.* (2012) Abdominal obesity vs general obesity for identifying arterial stiffness, subclinical atherosclerosis and wave reflection in healthy, diabetics and hypertensive. *BMC Cardiovasc Disord* **12**, 3.
22. Brunner EJ, Shipley MJ, Ahmadi-Abhari S *et al.* (2015) Adiposity, obesity, and arterial aging: longitudinal study of aortic stiffness in the Whitehall II cohort. *Hypertension* **66**, 294-300.
23. Marrugat J, Vila J, Baena-Diez JM *et al.* (2011) [Relative validity of the 10-year cardiovascular risk estimate in a population cohort of the REGICOR study]. *Rev Esp Cardiol* **64**, 385-394.
24. Marti R, Parramon D, Garcia-Ortiz L *et al.* (2011) Improving interMediAte risk management. MARK study. *BMC Cardiovasc Disord* **11**, 61.
25. Marrugat J, D'Agostino R, Sullivan L *et al.* (2003) An adaptation of the Framingham coronary heart disease risk function to European Mediterranean areas. *J Epidemiol Community Health* **57**, 634-638.
26. Conroy RM, Pyorala K, Fitzgerald AP *et al.* (2003) Estimation of ten-year risk of fatal cardiovascular disease in Europe: the SCORE project. *Eur Heart J* **24**, 987-1003.
27. Mancia G, Fagard R, Narkiewicz K *et al.* (2013) 2013 ESH/ESC Guidelines for the management of arterial hypertension: the Task Force for the management of arterial hypertension of the European Society of Hypertension (ESH) and of the European Society of Cardiology (ESC). *J Hypertens* **31**, 1281-1357.
28. Salas-Salvado J, Rubio MA, Barbany M *et al.* (2007) [SEEDO 2007 Consensus for the evaluation of overweight and obesity and the establishment of therapeutic intervention criteria]. *Med Clin (Barc)* **128**, 184-196. .
29. Browning LM, Hsieh SD, Ashwell M (2010) A systematic review of waist-to-height ratio as a screening tool for the prediction of cardiovascular disease and diabetes: 0.5 could be a suitable global boundary value. *Nutr Res Rev* **23**, 247-269.
30. Oliveros E, Somers VK, Sochor O *et al.* (2014) The concept of normal weight obesity. *Prog Cardiovasc Dis* **56**, 426-433.
31. Gomez-Ambrosi J, Silva C, Catalan V *et al.* (2012) Clinical usefulness of a new equation for estimating body fat. *Diabetes Care* **35**, 383-388.
32. Thomas DM, Bredlau C, Bosy-Westphal A *et al.* (2013) Relationships between body roundness with body fat and visceral adipose tissue emerging from a new geometrical model. *Obesity (Silver Spring)* **21**, 2264-2271.
33. Satoh N, Shimatsu A, Kato Y *et al.* (2008) Evaluation of the cardio-ankle vascular index, a new indicator of arterial stiffness independent of blood pressure, in obesity and metabolic syndrome. *Hypertens Res* **31**, 1921-1930.

For peer review only - http://bmjopen.bmj.com/site/about/guidelines.xhtml

34. Shirai K, Utino J, Otsuka K *et al.* (2006) A novel blood pressure-independent arterial wall stiffness parameter; cardio-ankle vascular index (CAVI). *J Atheroscler Thromb* **13**, 101-107.
35. Shirai K, Hiruta N, Song M *et al.* (2011) Cardio-ankle vascular index (CAVI) as a novel indicator of arterial stiffness: theory, evidence and perspectives. *J Atheroscler Thromb* **18**, 924-938.
36. Yamashina A, Tomiyama H, Takeda K *et al.* (2002) Validity, reproducibility, and clinical significance of noninvasive brachial-ankle pulse wave velocity measurement. *Hypertens Res* **25**, 359-364.
37. Catapano AL, Reiner Z, De Backer G *et al.* (2011) ESC/EAS Guidelines for the management of dyslipidaemias The Task Force for the management of dyslipidaemias of the European Society of Cardiology (ESC) and the European Atherosclerosis Society (EAS). *Atherosclerosis* **217**, 3-46.
38. O'Brien E, Asmar R, Beilin L *et al.* (2005) Practice guidelines of the European Society of Hypertension for clinic, ambulatory and self blood pressure measurement. *J Hypertens* **23**, 697-701.
39. Taylor HL, Jacobs DR, Jr., Schucker B *et al.* (1978) A questionnaire for the assessment of leisure time physical activities. *J Chronic Dis* **31**, 741-755.
40. Elosua R, Marrugat J, Molina L *et al.* (1994) Validation of the Minnesota Leisure Time Physical Activity Questionnaire in Spanish men. The MARATHOM Investigators. *Am J Epidemiol* **139**, 1197-1209.
41. Elosua R, Garcia M, Aguilar A *et al.* (2000) Validation of the Minnesota Leisure Time Physical Activity Questionnaire In Spanish Women. Investigators of the MARATDON Group. *Med Sci Sports Exerc* **32**, 1431-1437.
42. Ainsworth BE, Haskell WL, Leon AS *et al.* (1993) Compendium of physical activities: classification of energy costs of human physical activities. *Med Sci Sports Exerc* **25**, 71-80.
43. Haskell WL, Lee IM, Pate RR *et al.* (2007) Physical activity and public health: updated recommendation for adults from the American College of Sports Medicine and the American Heart Association. *Circulation* **116**, 1081-1093.
44. (2013) World Medical Association Declaration of Helsinki: ethical principles for medical research involving human subjects. *JAMA* **310**, 2191-2194.
45. Choi SY, Oh BH, Bae Park J *et al.* (2013) Age-associated increase in arterial stiffness measured according to the cardio-ankle vascular index without blood pressure changes in healthy adults. *J Atheroscler Thromb* **20**, 911-923.
46. Tomiyama H, Yamashina A, Arai T *et al.* (2003) Influences of age and gender on results of noninvasive brachial-ankle pulse wave velocity measurement--a survey of 12517 subjects. *Atherosclerosis* **166**, 303-309.
47. Marlatt KL, Kelly AS, Steinberger J *et al.* (2013) The influence of gender on carotid artery compliance and distensibility in children and adults. *J Clin Ultrasound* **41**, 340-346.
48. Bartok CJ, Marini ME, Birch LL (2011) High body mass index percentile accurately reflects excess adiposity in white girls. *J Am Diet Assoc* **111**, 437-441.
49. Philip R, Alpert BS, Schwingshackl A *et al.* (2015) Inverse Relationship between Cardio-Ankle Vascular Index and Body Mass Index in Healthy Children. *J Pediatr* **167**, 361-365 e361.
50. Yeboah K, Antwi DA, Gyan B *et al.* (2016) Arterial stiffness in hypertensive and type 2 diabetes patients in Ghana: comparison of the cardio-ankle vascular index and central aortic techniques. *BMC Endocr Disord* **16**, 53.
51. Al-Daghri NM, Al-Attas OS, Wani K *et al.* (2015) Sensitivity of various adiposity indices in identifying cardiometabolic diseases in Arab adults. *Cardiovasc Diabetol* **14**, 101.
52. Gomez-Sanchez L, Garcia-Ortiz L, Patino-Alonso MC *et al.* (2016) Association of metabolic syndrome and its components with arterial stiffness in Caucasian subjects of the MARK study: a cross-sectional trial. *Cardiovasc Diabetol* **15**, 148.
53. Morigami H, Morioka T, Yamazaki Y *et al.* (2016) Visceral Adiposity is Preferentially Associated with Vascular Stiffness Rather than Thickness in Men with Type 2 Diabetes. *J Atheroscler Thromb* **23**, 1067-1079.
54. Kawada T, Andou T, Fukumitsu M (2014) Relationship between cardio-ankle vascular index and components of metabolic syndrome in combination with sex and age. *Diabetes Metab Syndr* **8**, 242-244.

For peer review only - http://bmjopen.bmj.com/site/about/guidelines.xhtml

55. Zhou F, Zhang H, Yao W *et al.* (2014) Relationship between brachial-ankle pulse wave velocity and metabolic syndrome components in a Chinese population. *J Biomed Res* **28**, 262-268.
56. Kim HL, Lee JM, Seo JB *et al.* (2015) The effects of metabolic syndrome and its components on arterial stiffness in relation to gender. *J Cardiol* **65**, 243-249.
57. Weng C, Yuan H, Yang K *et al.* (2013) Gender-specific association between the metabolic syndrome and arterial stiffness in 8,300 subjects. *Am J Med Sci* **346**, 289-294.
58. Strasser B, Arvandi M, Pasha EP *et al.* (2015) Abdominal obesity is associated with arterial stiffness in middle-aged adults. *Nutr Metab Cardiovasc Dis* **25**, 495-502.
59. Danias PG, Tritos NA, Stuber M *et al.* (2003) Comparison of aortic elasticity determined by cardiovascular magnetic resonance imaging in obese versus lean adults. *Am J Cardiol* **91**, 195-199.
60. Takaki A, Ogawa H, Wakeyama T *et al.* (2008) Cardio-ankle vascular index is superior to brachial-ankle pulse wave velocity as an index of arterial stiffness. *Hypertens Res* **31**, 1347-1355.
61. Shirai K (2011) Analysis of vascular function using the cardio-ankle vascular index (CAVI). *Hypertens Res* **34**, 684-685.
62. Kubozono T, Miyata M, Ueyama K *et al.* (2007) Clinical significance and reproducibility of new arterial distensibility index. *Circ J* **71**, 89-94.
63. Nagayama D, Endo K, Ohira M *et al.* (2013) Effects of body weight reduction on cardio-ankle vascular index (CAVI). *Obes Res Clin Pract* **7**, e139-e145.

For peer review only

Table 1 General characteristics of all the sample and by gender.

Variables	Global (n=2354)	Males (n=1456)	Females (n=898)	p Value
Age (years)	61.4±7.7	61.1±8.1	61.8±7.0	0.030
Smoking n (%)	658 (28.0)	456 (31.5)	202 (22.7)	<0.001
Alcohol (gr/week)	72.2±117.5	102.2±133.3	23.6±59.9	<0.001
Physical activity (METs/min/week)	2481±2512	2886±1831	1825±1691	<0.001
Sedentary n (%)	1020 (43.3)	538 (37.0)	482 (53.7)	<0.001
Height (cm)	165±9	170±7	156±6	<0.001
Weight (kg)	79.4±14.6	83.9±13.4	72.2±13.3	<0.001
BMI (kg/m^2)	29.2±4.4	29.1±3.9	29.5±5.1	0.035
BMI ≥ 30 n (%)	847 (36.0)	485 (33.4)	362 (40.4)	0.001
Waist circumference (cm)	100.9±11.6	102.9±10.5	97.6±12.5	<0.001
WHtR	0.61±0.07	0.61±0.06	0.62±0.08	<0.001
CUN-BAE	35.7±1.7	31.1±4.5	43.1±5.1	<0.001
BRI	5.8±1.7	5.7±1.5	6.1±2.1	<0.001
SBP (mmHg)	137.1±17.4	138.9±17.1	134.2±17.5	<0.001
DBP (mmHg)	84.4±10.2	85.5±10.4	82.7±9.7	<0.001
Heart rate (beats minutes)	74.2±10.2	73.3±12.7	75.8±11.6	<0.001
Hypertension n (%)	1712 (72.7)	1122 (80.1)	590 (75.4)	<0.001
Antihypertensive drugs n (%)	1199 (50.9)	729 (50.2)	470 (52.6)	0.289
Total Cholesterol (mg/dl)	225.8±40.9	220.8±39.1	233.9±42.5	<0.001
LDL Cholesterol (mg/dl)	140.4±34.9	138.9±34.2	142.8±35.8	0.011
HDL Cholesterol (mg/dl)	49.8±12.9	47.9±11.9	52.9±13.8	<0.001
Triglycerides (mg/dl)	145.5±96.6	150.3±106.3	137.7±77.9	0.001
Atherogenic index	4.8±1.3	4.8±1.3	4.7±1.3	0.002
Dyslipidemia n (%)	2151 (91.4)	1311 (90.0)	840 (93.5)	<0.001
Lipid lowering drugs n (%)	671 (28.5)	392 (26.8)	279 (31.0)	0.034
FPG (mg/dl)	107.2±34.8	106.9±33.9	107.6±36.1	0.659
HbA1c	4.8±1.3	5.9±1.4	6.1±1.4	0.001
Diabetes n (%)	791 (33.6)	463 (31.8)	328 (36.5)	0.020
Antidiabetic drugs n (%)	474 (20.1)	269 (18.5)	205 (22.9)	0.011
CAVI	8.8±1.2	8.9±1.2	8.6±1.1	<0.001
baPWV (m/s)	14.9±2.5	14.8±2.5	15.0±2.6	0.107

Values are means and (standard deviations) for continuous data and number and (proportions) for categorical data.

Abbreviations: METs-min/week, metabolic equivalent minutes per week. BMI, body mass index. WHtR, waist-to-height ratio. CUN-BAE, University clinic of Navarra - body adiposity estimator. BRI, body roundness index. SBP, systolic blood pressure. DBP, diastolic blood pressure. LDL, low density lipoprotein. HDL, high density lipoprotein. FPG, fasting plasma glucose. HbA1c, glycosylated hemoglobin. CAVI, cardio ankle vascular index. baPWV, braquial-ankle pulse wave velocity.

p value differences between male and females

For peer review only

For peer review only - http://bmjopen.bmj.com/site/about/guidelines.xhtml

Table 2: Bivariate correlations of adiposity measures with CAVI and baPWV.

	CAVI		baPWV	
	Unadjusted	Adjusted [a]	Unadjusted	Adjusted [a]
**BMI**	-0.264**	-0.303**	-0.035	-0.068**
**WHtR**	-0.119**	-0.222**	0.090**	0.001
**CUN-BAE**	-0.187**	-0.297**	0.054*	-0.063**
**BRI**	-0.125**	-0.218**	0.078**	0.005

Abbreviations: CAVI, cardio-ankle vascular index. baPWV, brachial-ankle pulse wave velocity. BMI, body mass index. WHtR, waist-to-height ratio. CUN-BAE, University clinic of Navarra - body adiposity estimator. BRI, body roundness index.

[a] Adjusted for age, sex and systolic blood pressure.

p-values by Pearson correlation.* p<0.05, ** p< 0.01.

For peer review only

Table 3: Multiple regression analysis: association between adiposity measures with CAVI and baPWV.

	R^2	β(95%CI)	Partial R^2	p value
		**CAVI**		
**BMI**	0.412	-0.075 (-0.083 to -0.066)	0.055	<0.001
**WHtR**	0.381	-3.650 (-4.207 to -3.093)	0.038	<0.001
**CUN-BAE**	0.410	-0.069 (-0.077 to -0.061)	0.058	<0.001
**BRI**	0.378	-0.142 (-0.165 to -0.120)	0.037	<0.001
		**baPWV**		
**BMI**	0.402	-0.057 (-0.076 to -0.038)	0.007	<0.001
**WHtR**	0.394	-1.557 (-2.748 to -0.366)	0.001	0.021
**CUN-BAE**	0.401	-0.050 (-0.068 to -0.033)	0.007	<0.001
**BRI**	0.394	-0.066 (-0.115 to -0.018)	0.002	0.014

Four different multiple linear regression models were used to analyze the associations of adiposity measures with CAVI and baPWV.

Adjusted for age (years), gender (0=male and 1=female), systolic blood pressure, smoking (0=No and 1=Yes), METs/min/week, atherogenic index, HbA1c and METs/min/week.

Abbreviations: METs-min/week, metabolic equivalent minutes per week. CAVI, cardio-ankle vascular index. baPWV, brachial-ankle pulse wave velocity. BMI, body mass index. WHtR, waist-to-height ratio. CUN-BAE, University clinic of Navarra - body adiposity estimator. BRI, body roundness index. METs-min/week, metabolic equivalent minutes per week.

For peer review only

For peer review only - http://bmjopen.bmj.com/site/about/guidelines.xhtml

Table 4: Association between adiposity measures with CAVI in different groups.

	R^2	β(95%CI)	Partial R^2	p value
		**CAVI**		
Hypertensive				
**BMI**	0.409	-0.076 (-0.087 to -0.065)	0.023	<0.001
**WHtR**	0.383	-3.838 (-4.527 to -3.148)	0.035	<0.001
**CUN-BAE**	0.368	-0.068 (-0.078 to -0.058)	0.051	<0.001
**BRI**	0.382	-0.165 (-0.193 to -0.137)	0.035	<0.001
Non hypertensive				
**BMI**	0.372	-0.071 (-0.086 to -0.057)	0.052	<0.001
**WHtR**	0.324	-2.939 (-3.850 to -2.028)	0.046	<0.001
**CUN-BAE**	0.405	-0.065 (-0.080 to -0.052)	0.080	<0.001
**BRI**	0.318	-0.108 (-0.144 to -0.072)	0.042	<0.001
Diabetics				
**BMI**	0.439	-0.087 (-0.101 to -0.073)	0.95	<0.001
**WHtR**	0.399	-4.613 (-5.523 to -3.702)	0.069	<0.001
**CUN-BAE**	0.437	-0.083 (-0.096 to -0.070)	0.70	<0.001
**BRI**	0.391	-0.169 (-0.205 to -0.134)	0.062	<0.001
Non diabetics				
**BMI**	0.396	-0.067 (-0.078 to -0.056)	0.049	<0.001
**WHtR**	0.369	-3.088 (-3.781 to -2.396)	0.034	<0.001
**CUN-BAE**	0.395	-0.059 (-0.069 to -0.050)	0.064	<0.001
**BRI**	0.369	-0.130 (-0.159 to -0.099)	0.034	0.016
Obese				
**BMI**	0.414	-0.093 (-0.112 to -0.074)	0.054	<0.001
**WHtR**	0.377	-2.945 (-4.085 to -1.804)	0.003	<0.001
**CUN-BAE**	0.418	-0.100 (-0.122 to -0.079)	0.026	<0.001
**BRI**	0.377	-0.110 (-0.153 to -0.069)	0.003	<0.001
Non obese				
**BMI**	0.373	-0.058 (-0.078 to - 0.038)	0.013	<0.001
**WHtR**	0.361	-1.394 (-2.366 to - 0.421)	0.003	0.005
**CUN-BAE**	0.373	-0.050 (-0.066 to -0.033)	0.020	<0.001
**BRI**	0.358	-0.044 (-0.085 to -0.003)	0.002	0.034
≤ 62 years				
**BMI**	0.368	-0.078 (-0.089 to -0.077)	0.77	0.003
**WHtR**	0.332	-4.142 (-4.854 to -3.431)	0.065	<0.001
**CUN-BAE**	0.361	-0.068 (-0.078 to -0.058)	0.070	<0.001
**BRI**	0.324	-0.163 (-0.192 to -0.134)	0.062	<0.001
> 62 years				
**BMI**	0.234	-0.074 (-0.088 to -0.060)	0.056	<0.001
**WHtR**	0.195	-3.086 (-3.948 to -2.224)	0.029	<0.001
**CUN-BAE**	0.234	-0.071 (-0.084 to -0.059)	0.065	<0.001
**BRI**	0.193	-0.124 (-0.159 to -0.089)	0.028	<0.001
Assets				
**BMI**	0.414	-0.072 (-0.084 to -0.060)	0.053	<0.001
**WHtR**	0.392	-3.570 (-4.335 to -2.805)	0.034	<0.001
**CUN-BAE**	0.413	-0.066 (-0.075 to -0.053)	0.060	<0.001
**BRI**	0.391	-0.148 (-0.180 to -0.116)	0.034	<0.001
Sedentary				
**BMI**	0.408	-0.080 (-0.092 to -0.068)	0.055	<0.001
**WHtR**	0.364	-3.776 (-4.598 to -2.954)	0.045	<0.001
**CUN-BAE**	0.404	-0.074 (-0.086 to -0.062)	0.059	<0.001
**BRI**	0.360	-0.144 (-0.176 to -0.111)	0.043	<0.001

Multiple linear regression models were used to analyze the associations of adiposity measures with CAVI by groups.

Adjusted for age (years), gender (0=male and 1=female), Systolic blood pressure, smoking (0=No and 1=Yes), METs/min/week, atherogenic index, HbA1c and METs/min/week.

For peer review only

For peer review only - http://bmjopen.bmj.com/site/about/guidelines.xhtml

1 Abbreviations: METs-min/week, metabolic equivalent minutes per week. CAVI, cardio-ankle vascular index. BMI, body mass index. WHtR, waist-to-height ratio. CUN-BAE, University clinic of Navarra - body adiposity estimator. BRI, body roundness index.

For peer review only

For peer review only - http://bmjopen.bmj.com/site/about/guidelines.xhtml

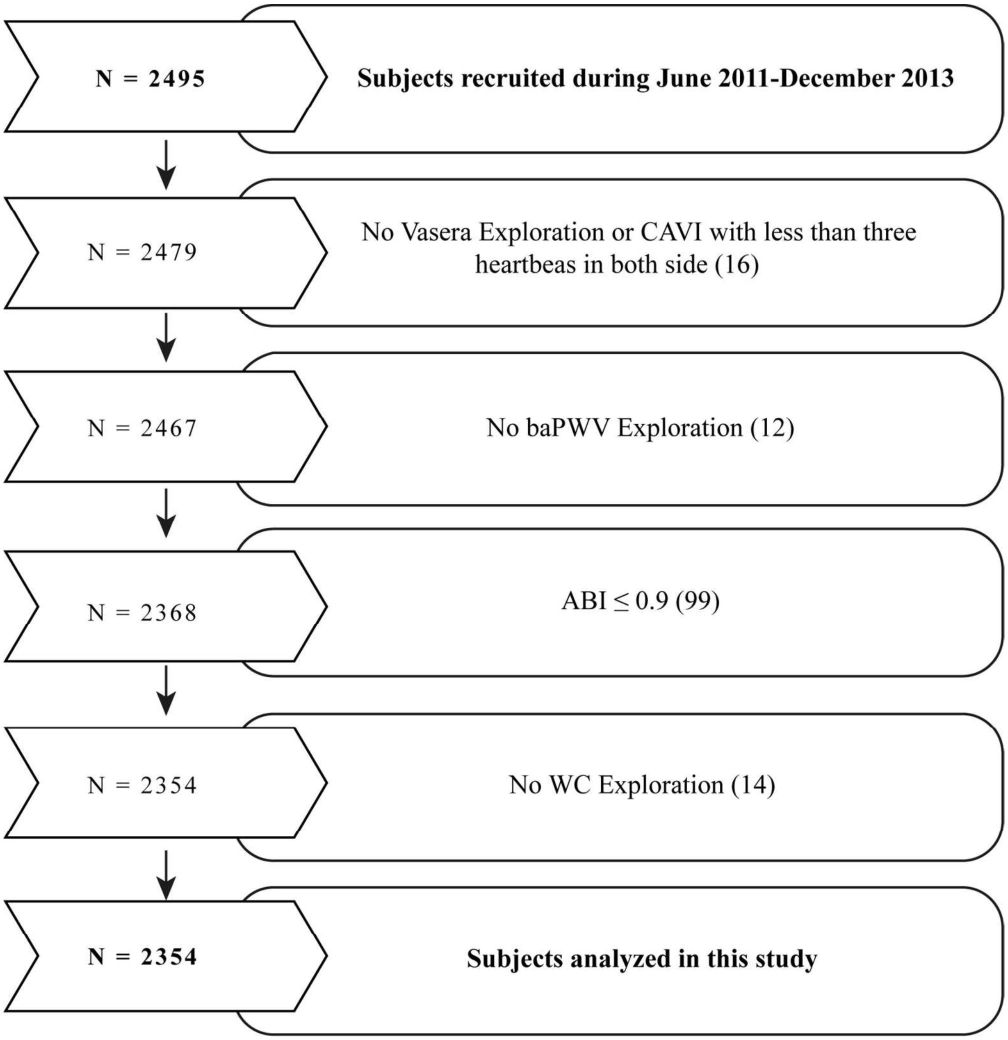

Figure 1. Flow chart of this MARK substudy
Abbreviations: N, number; CAVI, cardio-ankle vascular index; baPWV, brachial-ankle pulse wave velocity; ABI, ankle-brachial index; WC, waist circumference

145x149mm (300 x 300 DPI)

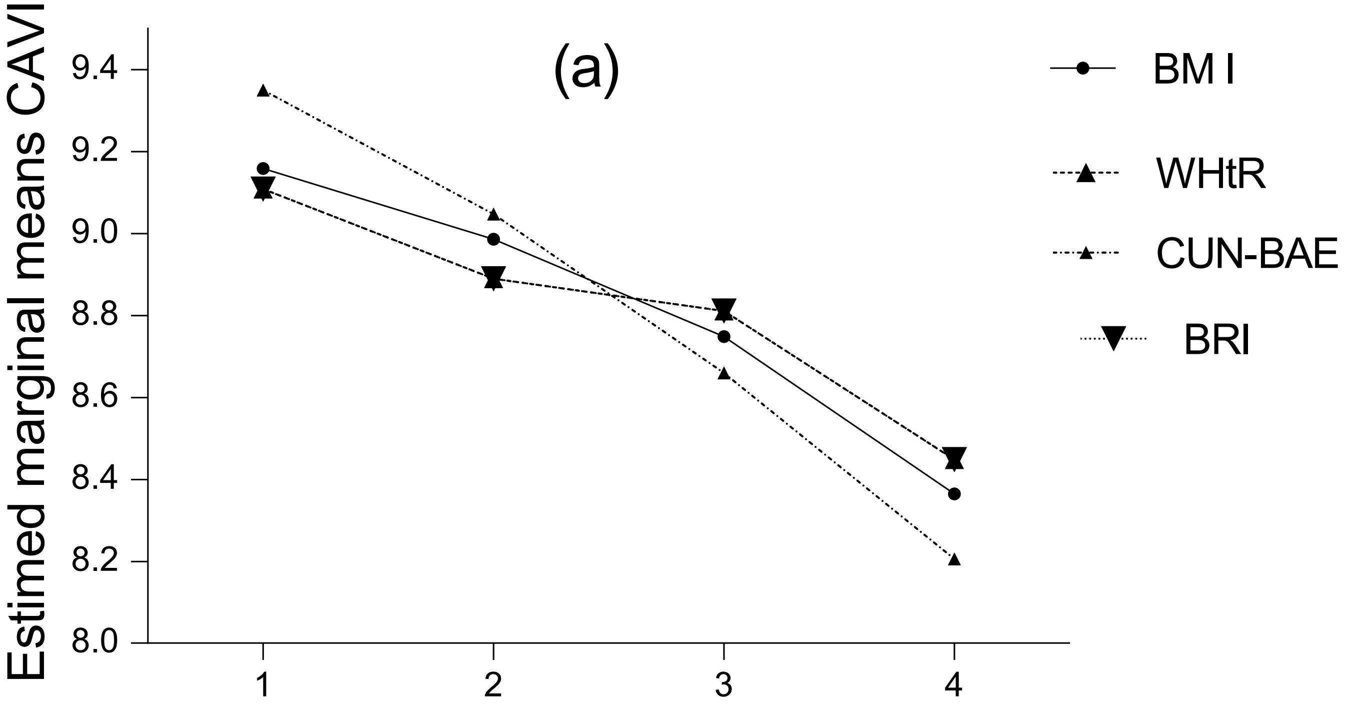
(a)
Estimed marginal means CAVI
9.4
9.2
9.0
8.8
8.6
8.4
8.2
8.0
1
2
3
4
BM I
WHtR
CUN-BAE
BRI

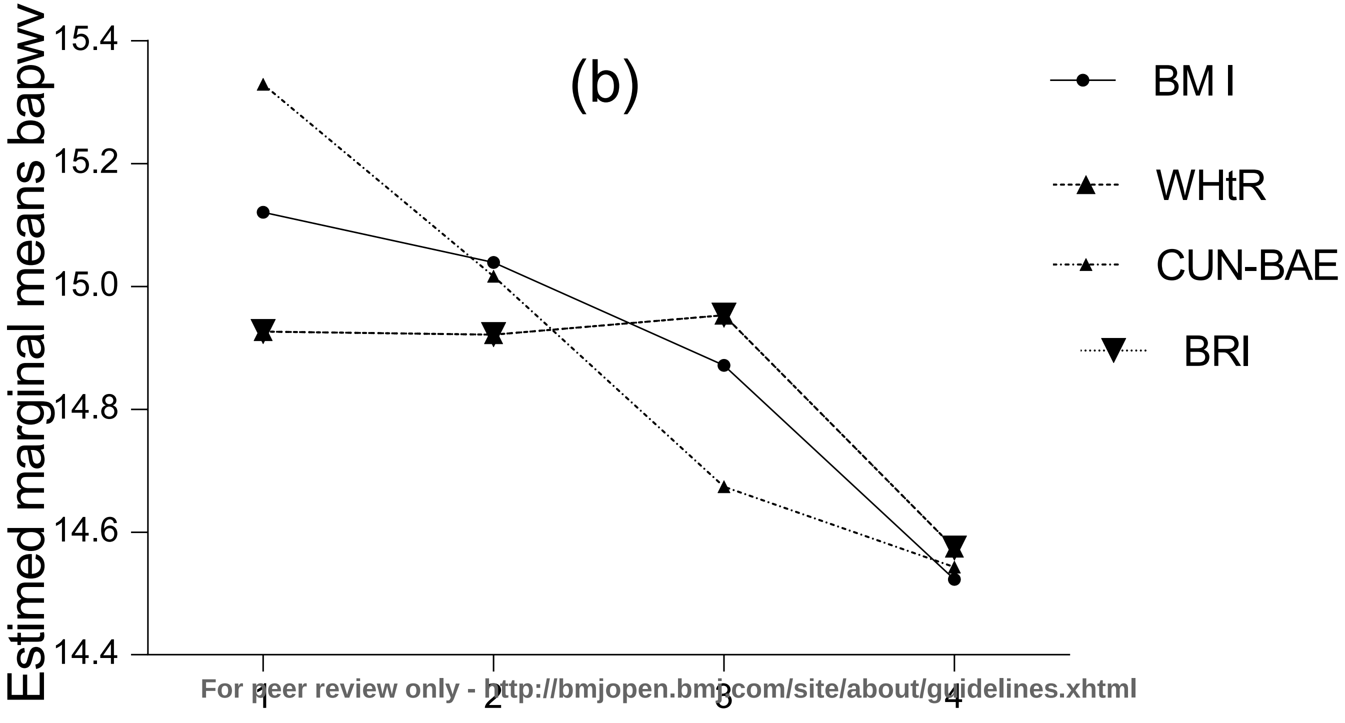
(b)
Estimed marginal means bapwv
15.4
15.2
15.0
14.8
14.6
14.4
1
2
3
4
BM I
WHtR
CUN-BAE
BRI

STROBE Statement—checklist of items that should be included in reports of observational studies

	Item No	Recommendation
**Title and abstract**	1	(*a*) Indicate the study's design with a commonly used term in the title or the abstract **Yes, Page 1**
		(*b*) Provide in the abstract an informative and balanced summary of what was done and what was found **Yes, Page 2**
**Introduction**		
Background/rationale	2	Explain the scientific background and rationale for the investigation being reported **Yes, Page 4**
Objectives	3	State specific objectives, including any prespecified hypotheses **Yes, Page 4**
**Methods**		
Study design	4	Present key elements of study design early in the paper **Yes, Page 5**
Setting	5	Describe the setting, locations, and relevant dates, including periods of recruitment, exposure, follow-up, and data collection **Yes, Page 5**
Participants	6	(*a*) *Cohort study*—Give the eligibility criteria, and the sources and methods of selection of participants. Describe methods of follow-up *Case-control study*—Give the eligibility criteria, and the sources and methods of case ascertainment and control selection. Give the rationale for the choice of cases and controls *Cross-sectional study*—Give the eligibility criteria, and the sources and methods of selection of participants **Yes, Page 5**
		(*b*) *Cohort study*—For matched studies, give matching criteria and number of exposed and unexposed *Case-control study*—For matched studies, give matching criteria and the number of controls per case **Yes, Page 5**
Variables	7	Clearly define all outcomes, exposures, predictors, potential confounders, and effect modifiers. Give diagnostic criteria, if applicable **Yes, Page 6**
Data sources/ measurement	8*	For each variable of interest, give sources of data and details of methods of assessment (measurement). Describe comparability of assessment methods if there is more than one group **Yes, Page 6-9**
Bias	9	Describe any efforts to address potential sources of bias **Yes, Page 8-9**
Study size	10	Explain how the study size was arrived at **Yes, Page 5**
Quantitative variables	11	Explain how quantitative variables were handled in the analyses. If applicable, describe which groupings were chosen and why **Yes, Page 8-9**

For peer review only - http://bmjopen.bmj.com/site/about/guidelines.xhtml

Statistical methods	12	(*a*) Describe all statistical methods, including those used to control for confounding **Yes, Page 8-9**
		(*b*) Describe any methods used to examine subgroups and interactions **Yes, Page 8-9**
		(*c*) Explain how missing data were addressed **Yes, Page 8-9**
		(*d*) *Cohort study*—If applicable, explain how loss to follow-up was addressed<br>*Case-control study*—If applicable, explain how matching of cases and controls was addressed<br>*Cross-sectional study*—If applicable, describe analytical methods taking account of sampling strategy
		(*e*) Describe any sensitivity analyses **Yes, Page 8-9**

Continued on next page

For peer review only

**Results**		
Participants	13*	(a) Report numbers of individuals at each stage of study—eg numbers potentially eligible, examined for eligibility, confirmed eligible, included in the study, completing follow-up, and analysed **Yes, Page 10-11**
		(b) Give reasons for non-participation at each stage **Yes, Page 10-11**
		(c) Consider use of a flow diagram **Yes, Figure 1**
Descriptive data	14*	(a) Give characteristics of study participants (eg demographic, clinical, social) and information on exposures and potential confounders **Yes, Page 10**
		(b) Indicate number of participants with missing data for each variable of interest **Yes, Page 10**
		(c) *Cohort study*—Summarise follow-up time (eg, average and total amount)
Outcome data	15*	*Cohort study*—Report numbers of outcome events or summary measures over time
		Case-control study—Report numbers in each exposure category, or summary measures of exposure
		Cross-sectional study—Report numbers of outcome events or summary measures
Main results	16	(*a*) Give unadjusted estimates and, if applicable, confounder-adjusted estimates and their precision (eg, 95% confidence interval). Make clear which confounders were adjusted for and why they were included **Yes, Tables**
		(*b*) Report category boundaries when continuous variables were categorized **Yes, tables**
		(*c*) If relevant, consider translating estimates of relative risk into absolute risk for a meaningful time period
Other analyses	17	Report other analyses done—eg analyses of subgroups and interactions, and sensitivity analyses **Yes, Page 10**
**Discussion**		
Key results	18	Summarise key results with reference to study objectives **Yes, Page 11**
Limitations	19	Discuss limitations of the study, taking into account sources of potential bias or imprecision. Discuss both direction and magnitude of any potential bias **Yes, Page 11**
Interpretation	20	Give a cautious overall interpretation of results considering objectives, limitations, multiplicity of analyses, results from similar studies, and other relevant evidence **Yes, Page 11-13**
Generalisability	21	Discuss the generalisability (external validity) of the study results **Yes, Page 11**
**Other information**		
Funding	22	Give the source of funding and the role of the funders for the present study and, if applicable, for the original study on which the present article is based **Yes, Page 15**

*Give information separately for cases and controls in case-control studies and, if applicable, for exposed and unexposed groups in cohort and cross-sectional studies.

Note: An Explanation and Elaboration article discusses each checklist item and gives methodological background and published examples of transparent reporting. The STROBE checklist is best used in conjunction with this article (freely available on the Web sites of PLoS Medicine at http://www.plosmedicine.org/, Annals of Internal Medicine at http://www.annals.org/, and Epidemiology at http://www.epidem.com/). Information on the STROBE Initiative is available at www.strobe-statement.org.

For peer review only

BMJ Open

Adiposity measures and arterial stiffness in primary care: the MARK prospective observational study

Journal:	*BMJ Open*
Manuscript ID	bmjopen-2017-016422.R2
Article Type:	Research
Date Submitted by the Author:	22-Jun-2017

Complete List of Authors:	Gomez-Sanchez, Leticia; La Alamedilla health centre., Primary Care Research Unit; 2. Biomedical Research Institute of Salamanca (IBSAL)<br>Garcia-Ortiz, Luis; La Alamedilla health centre. University of Salamanca, Primary care research unit La Alamedilla<br>Patino-Alonso, Maria; University of Salamanca, Salamanca, Spain , Statistics Department,<br>Recio-Rodriguez, Jose; La Alamedilla Health Center, Primary Care Research Unit, The Alamedilla Health Center, Castilla and León Health Service (SACYL), Biomedical Research Institute of Salamanca (IBSAL)<br>Frigo, Fernando<br>Marti, Rhut; Institut Universitari d'Investigació en Atenció Primària Jordi Gol (IDIAP Jordi Gol), Unitat de Suport a la Recerca de Girona.<br>Agudo-Conde, Cristina; La Alamedilla Health Center,, Research Unit<br>Rodriguez-Sanchez, Emiliano; Primary Care Research Unit, The Alamedilla Health Center, Salamanca, Spain, ; Biomedical Research Institute of Salamanca (IBSAL),<br>Maderuelo-Fernandez, Jose; Salamanca Institute for Biomedical Research. (IBSAL)., Primary care research unit La Alamedilla, Castilla and León Health Service (SACYL). Network of Preventive Activities and Health Promotion in Primary Care (REDIAPP)<br>Ramos, Rafel; Universidad de Girona. Departament Medicina<br>Gomez-Marcos, Manuel; the Alamedilla Health Center, Castilla and León Health Service (SACyL), Primary Care Research Unit; University of Salamanca,, Department of Medicine

**Primary Subject Heading**:	Cardiovascular medicine
Secondary Subject Heading:	Diabetes and endocrinology, Epidemiology, Nutrition and metabolism
Keywords:	Body mass index, Waist circumference, Waist-to-height ratio, Fat mass percent., Body roundness index., Arterial stiffness

SCHOLARONE™
Manuscripts

Title: Adiposity measures and arterial stiffness in primary care: the MARK prospective observational study

Authors: Leticia Gomez-Sanchez MD [1,2]*, Luis Garcia-Ortiz MD, PhD [1,2,3], Maria C Patino-Alonso PhD [1,2,4], Jose I Recio-Rodriguez PhD [1,2,5], Fernando Frigo MD, PhD [6], Rhut Martí PhD [7,8], Cristina Agudo-Conde [1,2], Emiliano Rodriguez-Sanchez MD, PhD [1,2,9], Jose A Maderuelo-Fernandez MD, PhD [1,2], Rafel Ramos MD, PhD [7,8,10] and Manuel A Gomez-Marcos MD, PhD [1,2,9] for the MARK Group [11]

1. Primary Care Research Unit, the Alamedilla Health Center, Castilla and León Health Service (SACyL), Salamanca, Spain
2. Biomedical Research Institute of Salamanca (IBSAL), Salamanca, Spain
3. Biomedical and diagnostic sciences department, University of Salamanca, Salamanca, Spain
4. Statistics Department, University of Salamanca, Salamanca, Spain
5. Department of Nursing and Physiotherapy, University of Salamanca, Salamanca, Spain
6. San Agustín Health Center, Illes Balears Health Service (IBSALUT), Palma of Mallorca, Spain
7. Unitat de Suport a la Recerca de Girona. Institut Universitari d'Investigació en Atenció Primària Jordi Gol (IDIAP Jordi Gol), Girona, Spain
8. Institut d'Investigació Biomèdica of Girona Dr. Josep Trueta (IDBGI). Girona, Spain
9. Department of Medicine, University of Salamanca, Salamanca, Spain
10. Departament of Ciències Mèdiques, Facultat de Medicina. Universitat de Girona, Spain.
11. MARK Group. redIAPP: Research Network in Preventive Activities and Health Promotion, Girona, Spain

***Corresponding Author:**

Leticia Gomez-Sanchez, Primary Care Research Unit, Alamedilla Health Care Center

37003 Salamanca, Spain

E-mail addresses: leticiagmzsnchz@gmail.com

Full list of author information is available at the end of the article.

For peer review only

ABSTRACT

Background: The cardiovascular risk in obesity is potentially increased by arterial stiffness.

Objective: This study investigates the relationship of adiposity measures with arterial stiffness in Caucasian adults with intermediate cardiovascular risk.

Setting: Six primary care centers in three Spanish Autonomous Communities, Spain

Participants: This study analyzed 2354 subjects (age range, 35–74 years; mean age, 61.4±7.7 years, 61.9% male).

Methods: The study was based on cross-sectional data from the MARK study. The main outcome variables were: body mass index (BMI), waist-to-height ratio (WHtR), Clínica Universidad de Navarra-body adiposity estimation (CUN-BAE) body fat percentage, and body roundness index (BRI). Vascular function was assessed by the cardio-ankle vascular index (CAVI) with the VaSera device, and brachial ankle pulse wave velocity (baPWV) was determined using a validated equation.

Results: The mean adiposity measures were a BMI of 29.2±4.4, WHtR of 0.61±0.07, CUN-BAE of 35.7±1.7, and BRI of 5.8±1.7. The mean stiffness measures were a CAVI of 8.8±1.2 and baPWV of 14.9±2.5. In multiple linear regression analysis, all adiposity measures were negatively associated with CAVI and baPWV ($p<0.01$ for all) after adjustment for cardiovascular risk factors. The proportion of CAVI variability by the adiposity measures were 5.5% for BMI, 5.8% for CUN-BAE, 3.8% for WHtR, and 3.7% for BRI, which were higher among diabetic, obese, younger (≤62 years), and non-hypertensive subjects who had similar activity and sedentary profiles.

Conclusions: Adiposity measures are negatively associated with arterial stiffness measures. The percentage of variation in CAVI explained by its relation to the different measures of adiposity ranges from 5.8% (CUN-BAE) to 3.7% (BRI). In the case of baPWV it oscillates between 0.7% (CUN-BAE and BMI) and 0.1% (WHtR).

For peer review only - http://bmjopen.bmj.com/site/about/guidelines.xhtml

Trial Registration: Clinical Trials.gov Identifier: https://clinicaltrials.gov/ct2/show/ NCT01428934. Registered 2 September 2011. Last updated September 8, 2016.

Keywords: body mass index, waist circumference, waist-to-height ratio, fat mass percent, body roundness index, arterial stiffness

Strengths and limitations of this study

- This is the first study to investigate the association between adiposity measures using a cardio-ankle vascular index with and brachial ankle pulse wave velocity in Caucasian adults with intermediate cardiovascular risk**.**
- All adiposity measures were negatively associated with CAVI and baPWV.
- The arterial stiffness variability was better explained for BMI and CUN-BAE and when CAVI was used as a measure of stiffness rather than baPWV.
- The main limitation of our study is its cross-sectional design, which does not allow establishment of causal relationships or the direction in which adiposity measures influence vascular function.

For peer review only - http://bmjopen.bmj.com/site/about/guidelines.xhtml

INTRODUCTION

Obesity has been linked to increased all-cause and cardiovascular mortality [1]. However, the mechanisms through which obesity can increase the frequency of cardiovascular disease beyond traditional risk factors are not clearly identified [2]. It has been suggested that, increased arterial stiffness may be a mechanism by which obesity increases cardiovascular risk independently of traditional risk factors[3]. It is known that arterial stiffness as evaluated by the brachial ankle pulse wave velocity (baPWV) is an independent predictor of coronary heart disease and mortality in both the general population [4] and in patients with diabetes mellitus [5; 6]. Similarly, the cardio-ankle vascular index (CAVI) is associated with carotid and coronary atherosclerosis [7; 8; 9] and is a predictor of cardiovascular events in obese patients [10]. I. However, the relationship between adiposity and arterial stiffness remains controversial. In this respect, there are studies that show that the body mass index (BMI) has been associated with arterial stiffness in the general population [3; 11] and in diabetic patients [12; 13]. However, other research has not found this association [14], or the association disappeared after adjusting for potential confounders [13], or it showed a negative association [15; 16]. Additionally, there are studies that suggest a stronger correlation of measures of central or visceral adiposity than measures of general adiposity with arterial stiffness in the general population [3; 17; 18; 19; 20], in diabetic patients [13], and in diabetics and hypertensive patients [21]. Finally the Whitehall II Cohort study [22] showed that all measures of general adiposity, central adiposity, and body fat percentage were predictors of accelerated arterial stiffness in adults. In this context the analysis of the relationship between arterial stiffness and different measures of adiposity can help to understand the role of obesity in cardiovascular disease.

Our study was designed bearing in mind that cardiovascular events are more likely to occur in patients with intermediate cardiovascular risk [23], and that there is a lack of studies analyzing the relationship of different adiposity measures with arterial stiffness in these subjects. We have established the following objectives: the primary aim of this study was to investigate the

For peer review only - http://bmjopen.bmj.com/site/about/guidelines.xhtml

relationship between adiposity measures and arterial stiffness in Caucasian adults with intermediate cardiovascular risk. The secondary aim was to analyze the differences between the associations of adiposity measures with distinct arterial stiffness markers. (Page 4 and 5).

METHODS

Study design

This trial was a cross-sectional study of subjects recruited to the *improving interMediAte RisK management (MARK)* study (NCT01428934) [(24)], which was a longitudinal study designed to assess whether the ankle-brachial index, arterial stiffness (measured by CAVI), postprandial glucose, glycosylated hemoglobin, self-measured blood pressure, and the presence of comorbidities were independently associated with the occurrence of vascular events. It also investigated whether the predictive capacity of current risk equations could be improved in the intermediate risk population. The current study focused on the baseline visit. The second step will be a 5- and 10-year follow-up trial to assess cardiovascular morbidity and mortality.

Study population

In this multicenter project, the study population was obtained by random sampling from subjects who met the inclusion criteria and who were seeing general practitioners from July 2011 to June 2013 at six primary care centers in three Spanish Autonomous Communities. Subjects were recruited from those aged 35 to 74 years with an intermediate cardiovascular risk, defined as a 10-year coronary risk ranging from 5%–15% according to the adapted Framingham risk equation [(25)]; 10-year vascular mortality risk greater than 1% and less than 5% according to the SCORE equation [(26)]; or a moderate risk according to the European Society of Hypertension guidelines for the management of arterial hypertension [(27)]. Exclusion criteria included end-stage disease or institutionalization at the time of the visit, or history of atherosclerotic disease. This study analyzed 2354 of the 2495 subjects recruited in the MARK

For peer review only - http://bmjopen.bmj.com/site/about/guidelines.xhtml

study. For the present analysis, we excluded 141 individuals, with ABI ≤ 0.9 (n=99), or which CAVI (n=16), baPWV (n=12) and WC (n = 14) measurements were incomplete (Figure 1).

Variables and measurement instruments

A detailed description of procedures for clinical data collection and laboratory tests has been published elsewhere [24].

Anthropometric measurements

Body weight was measured twice using a certified electronic scale (Seca 770, Medical scale and measurement systems, Birmingham, United Kingdom) after adequate calibration (precision ± 0.1 kg). Readings were rounded-off to the nearest 100 g. Height was measured using a stadiometer (Seca 222), and the average of two measurements was recorded. Body mass index (BMI) was calculated as weight (kg) divided by height squared (m^2). Waist circumference was measured according to the 2007 recommendations of the Spanish Society for the Study of Obesity [28]. All measurements were performed with the subjects standing, wearing no shoes, and in light clothing. Waist-to-height ratio (WHtR) was calculated as the waist circumference (cm) divided by height (cm) [29; 30].

The body fat percentage (BF%) was calculated according to the Clínica Universidad de Navarra-body adiposity estimator (CUN-BAE) using the recommendations of Gomez-Ambrosi et al. [31]: $BF\% = -44.988 + (0.503 \times Age) + (10.689 \times sex) + (3.172 \times BMI) - (0.026 \times BMI^2) + (0.181 \times BMI \times sex) - (0.02\ BMI \times Age) - (0.005 \times BMI^2 \times sex) + (0.00021 \times BMI^2 \times Age)$, where male=0 and female=1 for sex.

For peer review only - http://bmjopen.bmj.com/site/about/guidelines.xhtml

Body roundness index (BRI) was calculated using the following formula: BRI= 364.2 – 365.5 × $\sqrt{1-(\frac{(WC/(2\pi))^2}{(0.5height)^2})}$ [32]. BRI can predict the percentage of body fat and visceral adipose tissue.

Cardio-ankle vascular index (CAVI) and brachial-ankle pulse wave velocity (baPWV)

CAVI was measured using a *VaSera VS-1500®* device *(Fukuda Denshi)* [33; 34]. CAVI values were calculated automatically by estimating the stiffness parameter β using the following equation: $\beta = 2\rho \times 1 / (Ps - Pd) \times \ln (Ps / Pd) \times PWV^2$, where ρ was blood density, Ps and Pd were SBP and DBP in mmHg, and PWV was measured between the aortic valve and the ankle [35]. The mean coefficient of variation of CAVI measurement was less than 5%, which is small enough to allow for clinical use of the index and confirmed that CAVI was reproducible index [34].

CAVI was calculated using the VaSera VS-1500® device (Fukuda Denshi), and with the values obtained, the baPWV was estimated using the equation baPWV= (0.5934 × height (cm) + 14.4724)/tba (where tba was the time interval between the arm and ankle waves) [36].

Measurements were performed with the patient in the supine position after resting for 10 minutes in a quiet room at a stable temperature. Subjects were instructed not to smoke or exercise in the hour before the test.

Diagnosis of cardiovascular risk factors

Subjects were considered to be hypertensive if they were previously diagnosed hypertension, if they were taking antihypertensive drugs, or if they had blood pressure levels ≥ 140/90 mmHg. Diabetic subjects were those who had been previously diagnosed with the disease, were taking hypoglycemic drugs, or had a fasting blood glucose levels ≥ 126 mg/dL or HbA1c ≥ 6.5%. Dyslipidemia was defined if they were treated with lipid-lowering drugs or

For peer review only - http://bmjopen.bmj.com/site/about/guidelines.xhtml

For peer review only

had altered LDL ≥ 130 mg/dl, HDL ≤ 45 mg/dl in men and ≤ 55 in women, and TG ≥ 150 mg/dl, as established by the European Society of Cardiology (ESC) and the European Atherosclerosis Society (EAS) 2011 [37].

Office or clinical blood pressure

Office blood pressure measurement involved three measurements of SBP and DBP using a validated OMRON model M10-IT sphygmomanometer (Omron Health Care, Kyoto, Japan). The measurements followed the recommendations of the European Society of Hypertension [38], and the average of the last two measurements was used.

Lifestyles

Tobacco

Smoking history was assessed by asking questions about the participant's smoking status (smoker/non-smoker). We considered smokers to include those who currently smoke or who had stopped smoking within the past year.

Leisure time physical activity

Leisure time physical activity (LTPA) was collected using the Minnesota LTPA Questionnaire [39] that was validated for Spanish men and women[40; 41]. The questionnaire was administered by trained interviewers who spent about 10 to 20 min per participant collecting detailed information about physical activity (PA) during the preceding year, the number of times this activity was performed, and the average duration of each activity on each occasion. Each PA has an intensity code, based on the ratio between the metabolic rate during PA practice and the basal metabolic rate (MET) [42]. It is assumed that 1 MET corresponds to approximately 1 kcal/min of energy expenditure. Therefore, we can calculate the total energy expenditure in leisure time of PA ($EEPA_{total}$) in kilocalories per week. Moreover, based on the PA intensity code, we could quantify the energy expenditure in physical activity (EEPA) according to the activity's classification as intense, moderate, or light intensity as follows: light PA intensity

For peer review only - http://bmjopen.bmj.com/site/about/guidelines.xhtml

was below 4 METs, such as walking (EEPA $_{light}$). Moderate PA intensity was 4–5.5 METs, such as brisk walking (EEPA $_{moderate}$). Intense PA intensity was greater than or equal to 6 METs, such as jogging (EEPA $_{intense}$). Thus, for each particular subject: EEPA $_{total}$ = EEPA $_{light}$ + EEPA $_{moderate}$ + EEPA $_{intense}$.

Based on recommendations from the American Heart Association [43], we considered those participants who do not meet the recommendations of moderate-intensity aerobic PA practice for a minimum of 30 min on 5 days each week (EEPA $_{moderate}$ < 675 kcal/week) or high-intensity aerobic PA practice for a minimum of 20 min on 3 days each week (EEPA $_{intense}$ < 420 kcal/week) to be sedentary.

Laboratory determinations

Venous blood sampling was performed between 08:00 and 09:00 hours after the individuals fasted and abstained from smoking and the consumption of alcohol and caffeinated beverages for the previous 12 hours. Fasting plasma glucose, serum, high-density lipoprotein (HDL) cholesterol concentrations and triglyceride concentrations were measured using standard enzymatic automated methods. Low-density lipoprotein cholesterol was estimated using the Friedewald equation when the direct parameter was not available. The atherogenic index was estimated (total cholesterol / HDL cholesterol). Blood samples were collected at the Health Center and analyzed at the hospital of reference.

The researchers who performed the different tests were blinded to the clinical data of the subjects. All assessments were made within a period of 10 days.

Data analysis

Continuous variables were expressed as the mean ± standard deviation. Frequency distributions were used for categorical data. Comparisons between quantitative variables were performed using the Student's *t*-test, while the chi-squared test and Fisher's exact test were used for qualitative variables. Pearson's correlation coefficient was used to estimate the

For peer review only - http://bmjopen.bmj.com/site/about/guidelines.xhtml

relationship of the adiposity measures to CAVI and baPWV. We used Steiger's Z statistics for testing the significance of the difference between correlations coefficients [44].

Four different multiple linear regression models were used to analyze the associations of each adiposity measure with CAVI, and four other models were used with baPWV. CAVI and baPWV were the dependent variables, and the adiposity measures were the independent variables in each model. All models were adjusted for age (years), sex (0 = male and 1 = female), systolic blood pressure (SBP), smoking status (0 = No and 1 = Yes), atherogenic index, HbA1c, and METs/min/week. The explanatory capacity of the model was measured by R^2, and the proportion attributed to each variable was estimated by the change in R^2. The analysis was also performed by age groups, diagnosis of diabetes, hypertension, and obesity.

ANCOVA models were used to test the differences in the mean values of CAVI and baPWV with the quartiles of the four adiposity measures after adjusting for the confounding variables that were used in the regression analysis. Pairwise post hoc comparisons were examined using the Bonferroni test. Data were analyzed using SPSS Statistics for Windows version 23.0 (IBM Corp, Armonk, NY). Values of $p<0.05$ were considered statistically significant.

Ethics statement

All participants were informed of the study objectives and procedures and they all signed an informed consent form to participate. The study was approved by the Clinical Research Ethics Committee of the Primary Care Research Institute Jordi Gol, the Health Care Area of Salamanca, and Palma of Mallorca. The study was conducted following the recommendations of the Declaration of Helsinki [45]. The confidentiality of information provided by participants was ensured, complying with the rules established by Spanish Organic Law 15/1999, of 13 December on the Protection of Personal Data.

For peer review only - http://bmjopen.bmj.com/site/about/guidelines.xhtml

RESULTS

Anthropometric measures, clinical characteristics, and vascular function measures of the subjects are presented in Table 1. The mean age of the patients was 61.4±7.7 years, and 61.9% were male. Male subjects had a higher percentage of smokers (31.5 *vs.* 22.7) and hypertension (80.1 *vs.* 75.4) compared with females. However, females had a higher prevalence of obesity (40.4 *vs.* 33.4), sedentariness (53.7 vs. 37.0), dyslipidemia (73.1 *vs.* 63.6), and diabetes (36.5 *vs.* 31.8) compared with males. The mean value of CAVI was 8.8±1.2 (8.9 in males and 8.6 in females, $p<0.001$) and the mean baPWV was 14.9±2.5 (14.8 in males and 15.0 in females). All of the analyzed adiposity measures except for waist circumference presented higher values in women compared with men.

Pearson's correlation coefficient results between the adiposity measures and the vascular function parameters are shown in Table 2. All adiposity measures were negatively correlated with CAVI, and this correlation increases after adjusting for age, sex, and SBP. The correlation between CAVI and baPWV was $r=0.745$ ($p<0.001$). We found differences in correlation coefficients between CAVI, baPWV and measures of adiposity ($p<0.001$ in all cases).

Figure 1S (Supplementary material) shows the estimated marginal means of CAVI (a) and baPWV (b) by quartiles of the different adiposity measures. After adjustment for the variables used in the multiple linear regression analysis, the mean CAVI values decreased as the quartiles of the four adiposity measurements increased ($p<0.05$). However, the same is not true of baPWV with WHtR and BRI ($p>0.05$).

In the multiple linear regression analysis, CAVI and baPWV showed negative associations with all adiposity measures ($p<0.01$ for all) after adjustment for age, sex, SBP, smoking, atherogenic index, HbA1c, and METs/min/week (Table 3). The proportion of CAVI

For peer review only - http://bmjopen.bmj.com/site/about/guidelines.xhtml

variability which can be attributed to the variation in the adiposity measures was 5.5% for BMI, 5.8% for CUN-BAE, 3.8% for WHtR, and 3.7% for BRI. 5.5% for BMI, 5.8% for CUN-BAE, 3.8% for WHtR, and 3.7% for BRI. For baPWV, the variability by the measures of adiposity were 0.7% for BMI, and CUN-BAE, and 0.1% for WHtR, and 0.2 for BRI. The association between adiposity measurements and CAVI revealed standardized β between -0.450 (CUN-BAE) and -0.221 (WHtR). In the case of baPWV the values oscillate between -0.152 (CUN-BAE) and -0.044 (WHtR).

In the multiple linear regression analysis by subgroup, the proportion of CAVI variability by adiposity measures was higher among diabetics, obese, non-hypertensive, and subjects 62 years of age or younger, and was similar in active and sedentary people (Table 4).

DISCUSSION

The results of this study show that adiposity measures have a negative association with arterial stiffness, especially CAVI. BMI and CUN-BAE are the ones with the highest coefficient of determination. We found a negative association of different adiposity measures with CAVI and baPWV after adjustment for other cardiovascular risk factors.

In this study the mean value of CAVI was higher in males, which is in agreement with published data indicating that CAVI increases linearly with age, and is higher in males than in females (approximately 0.2, which is equivalent to 4–5 years old) [22; 46]. Similarly, there was no difference in the mean baPWV between the sexes, which is consistent with data published by Tomiyama et al. [47] who showed that the effect of age on baPWV is different according to sex. Females have a higher arterial stiffness than prepubertal males, and this increases after menopause. Men, however, experience a linear increase in arterial stiffness from puberty. This suggests that women have large arteries that are intrinsically more rigid compared with men, but these effects are mitigated by sex steroids during the reproductive years [48; 49].

For peer review only - http://bmjopen.bmj.com/site/about/guidelines.xhtml

For peer review only

This negative association with CAVI has already been described in previous studies. BMI has shown a negative association in children [(50)], and in hypertensive and type 2 diabetes patients in Ghana [(51)]. Similarly, waist circumference has shown a negative relationship in subjects with metabolic syndrome [(52; 53)].

However, other authors have described a positive association of different adiposity measures with the β-stiffness parameter, but after adjustment for age and other possible confounding factors, the association remained for only men with type 2 diabetes mellitus [(54)]. Other studies have also found no association between BMI and CAVI [(55)].

Studies analyzing the association of adiposity measures with baPWV have also been performed mainly in Eastern populations and have focused on assessing the association between BMI and waist circumference as measures of adiposity. The results are controversial, with some finding a negative association with BMI [(47)], and with waist circumference in men only or in women only [(56; 57)]. However, other studies have found a positive correlation with BMI and waist circumference [(58)]. One study that analyzed the association of different adiposity parameters with baPWV in middle-aged adults found a positive association with waist circumference and visceral fat but not with body fat percentage [(59)].

The results are also not consistent in studies that used the carotid-femoral pulse wave velocity (cfPWV) as a measure of stiffness. Some studies have described a greater association with measures of central or visceral adiposity in both the general population and in diabetics [(3; 13; 17; 18; 19; 21)]. However, Strasser et al. [(59)] found no association with body fat percentage, and other studies also found no association between BMI and cfPWV [(13; 14)]. Rodrigues et al. [(15)], reported that BMI was negatively associated with cfPWV ($\beta = -0.103$) in a large population sample. The Whitehall II Cohort study [(22)], which was completed based on staff lists from offices located in central London, showed that all measures of adiposity were robust predictors of accelerated cfPWV, after adjusting them for potential confounding factors. The

For peer review only - http://bmjopen.bmj.com/site/about/guidelines.xhtml

use of different measures to measure arterial stiffness such as CAVI and baPWV, as well as being a population with intermediate cardiovascular risk could explain some of the discrepancies with our study.

Arterial stiffness depends on arterial wall elasticity and diameter, and a positive correlation was found between BMI and aortic diameter measured by nuclear magnetic resonance [60]. This could partially explain the negative association between measures of adiposity and arterial stiffness. These discrepancies between studies may be partially explained by different methods of arterial stiffness measurement and the adjustment variables used. This may be because CAVI reflects central and peripheral arterial stiffness and is less influenced by blood pressure values at the time of measurement [35; 61; 62]. Conversely, arterial stiffness assessed using baPWV is a measure of peripheral arterial stiffness [36]. Other potential influences on the observed differences are age, sex, race, prevalent cardiovascular risk, and drugs used for treatment of the different risk factor [12; 47; 56; 58]. These differences between CAVI and baPWV, as measures of rigidity, could explain the results shown in this study, suggesting a greater association of adiposity measurements with CAVI than with baPWV.

The proportion of baPWV variability explained by adiposity measurements in our study was less than 1% (between 0.7% for BMI and 0.1% for CUN-BAE). The results are also lower than those published for the general population by Wohlfahrt et al. (5% for WHtR and 3% for BMI) [3], but similar to the results reported by Rodrigues et al. (BMI 0.7%) [15]. The proportion of CAVI variability that is explained by adiposity measures was higher than 5% with CUN-BAE and BMI and higher than 3.5% with WHtR and BRI. In the subgroup analysis, the proportion of CAVI variability explained by adiposity measures was higher in diabetic, obese, younger, and non-hypertensive subjects. Our results show that the influence of adiposity measurements on CAVI is greater than on baPWV. To our knowledge, no other study has analyzed this aspect using CAVI. The novel results of this study may have

For peer review only - http://bmjopen.bmj.com/site/about/guidelines.xhtml

important clinical relevance because they show the associations of both general and abdominal obesity measures with CAVI and baPWV in subjects with intermediate cardiovascular risk. Additionally, the results provide information that could be used in new prospective studies and could potentially help to improve cardiovascular risk equations.

Numerous studies have been carried out analyzing the effect of weight loss on arterial stiffness, most of which have been collected in two meta-analyzes. The first analyzed the results of 20 studies (1259 participants), and showed that modest weight loss (8% of initial body weight) achieved with diet and lifestyle interventions seems to improve PWV. The standardized mean difference (SMD) for the overall effect of weight loss on baPWV measured at all sites was -0.32 (95% CI, -0.41, -0.24; P=0.0001). cfPWV (SMD, -0.35; 95% CI, -0.44, -0.26; P=0.0001; 16 studies) and baPWV (SMD, -0.48; 95% CI, -0.78, -0.18; P=0.002; 5 studies) improved with weight loss. [63]. In the second meta-analysis, 43 studies (4231 participants) were included and it was found that the average weight loss was approximately 11% of the initial body weight and that weight loss improved CAVI (SMD= -0.48; p = 0.04) [64].

In summary, our results showed the correlation between measures of adiposity and measures of arterial stiffness is greater with CAVI than with baPWV. The different measures of adiposity better explained the variability of arterial stiffness evaluated using CAVI than using baPWV. All this suggests that the relationship with adiposity measures is greater if the arterial stiffness is measured using CAVI than using baPWV. This is likely because CAVI measures rigidity at the central and peripheral levels and it is not influenced by blood pressure at the time of measurement [35; 61; 65; 66].

The main limitation of our study is its cross-sectional design, which does not allow us to establish causal relationships or the direction of influence of adiposity measures in vascular function. Another limitation is that the population was ethnically homogeneous (all subjects

For peer review only - http://bmjopen.bmj.com/site/about/guidelines.xhtml

were Caucasians with intermediate cardiovascular risk). Therefore, the extrapolation of our findings may be limited.

CONCLUSION

In conclusion, the adiposity measures analyzed show a negative association with arterial stiffness measures. The percentage of variation in CAVI explained by its relation to the different measures of adiposity ranges from 5.8% (CUN-BAE) to 3.7% (BRI). In the case of baPWV it oscillates between 0.7% (CUN-BAE and BMI) and 0.1% (WHtR). These results suggest that measures of general adiposity and body fat percentage better explain the variability of CAVI compared with measures of abdominal and visceral adiposity.

Acknowledgments: We are grateful to all professionals participating in the MARK study. Lead author for this group: Rafel Ramos, Research Unit, Primary Health Care, Girona, Jordi Gol Institute for Primary Care Research (IDIAP Jordi Gol), Catalonia, Spain, E-mail: rramos.girona.ics@gencat.net. Coordinating Center: Rafel Ramos, Ruth Martí, Dídac Parramon, Anna Ponjoan, Miquel Quesada, Maria Garcia-Gil, Martina Sidera and Lourdes Camós, Research Unit, Primary Health Care, Jordi Gol Institute for Primary Care Research (IDIAP Jordi Gol), C/Maluquer Salvador, 11, 17002-Girona, Catalonia, Spain. Fernando Montesinos, Ignacio Montoya, Carlos López, Anna Agell, Núria Pagès of the Primary Care Services, Girona, Catalan Institute of Health (ICS), Catalonia, Spain. Irina Gil, Anna Maria-Castro of the Primary Care Services, Girona, Institut d'Assistència Sanitaria (IAS), Catalonia, Spain. Fernando Rigo, Guillermo Frontera, Antònia Rotger, Natalia Feuerbach, Susana Pons, Natividad Garcia, John Guillaumet, Micaela Llull and Mercedes Gutierrez of the Health Center Primary Care San Augustín, Ibsalut Balears, Spain. Cristina Agudo-Conde, Leticia Gómez-Sanchez, Carmen Castaño-Sanchez, Carmela Rodriguez-Martín, Benigna Sanchez-

For peer review only - http://bmjopen.bmj.com/site/about/guidelines.xhtml

For peer review only

Salgado, Angela de Cabo-Laso, Emiliano Rodriguez-Sanchez, Jose Angel Maderuelo-Fernandez, Marta Gómez-Sánchez, Emilio Ramos-Delgado, Carmen Patino-Alonso, Jose I Recio-Rodriguez, Manuel A Gomez-Marcos and Luis Garcia-Ortiz, Primary Care Research Unit of The Alamedilla, Salamanca, Spain, Castilla and León Health Service–SACYL.

Contributors: LG-S designed the study, wrote the protocol, participated in fundraising, interpreted the results, prepared the manuscript draft, performed all analytical testing, interpreted the results and reviewed the manuscript and corrected the final version of the manuscript. JIR-R, RM, FR, and CA-C participated in the study design, data collection and manuscript review. ER-S and JAM-F participated in the study design, interpretation of results and manuscript review. MCP-A participated in the analysis of results and final review of the manuscript. LG-O, RR, and MAG-M participated in the protocol design, fundraising, analysis of results, and final review of the manuscript. All authors reviewed and approved the final version of the manuscript.

Funding: This research was supported by grants from the Spanish Ministry of Science and Innovation (MICINN), the Carlos III Health Institute/European Regional Development Fund (ERDF) (MICINN, ISCIII/FEDER) (Red RedIAPP RD06/0018, Research Groups: RD16/0007/0003, RD16/0007/0004, RD16/0007/0008), the Health Research Fund (PI10/01088, PI10/02077, PI10/02043, PI13/01930), and the Regional Health Management of Castile and León (GRS 635/A/11; GRS 906/B/14).

Competing interests: None declared.

For peer review only

For peer review only - http://bmjopen.bmj.com/site/about/guidelines.xhtml

Ethics approval: The Clinical Research Ethics Committee of the Primary Care Research Institute Jordi Gol, the Health Care Area of Salamanca and Palma of Mallorca.

Provenance and peer review: Not commissioned; externally peer reviewed.

Data sharing statement: No additional data are available.

Informed consent: Obtained.

Open Access: This is an Open Access article distributed in accordance with the Creative Commons Attribution Non Commercial (CC BY-NC 4.0) license, which permits others to distribute, remix, adapt, build upon this work non commercially, and license their derivative works on different terms, provided the original work is properly cited and the use is non-commercial. See: http:// creativecommons.org/licenses/by-nc/4.0/

For peer review only

Figure legends

Figure 1. Flow chart of this MARK substudy

Abbreviations: N, number; CAVI, cardio-ankle vascular index; baPWV, brachial-ankle pulse wave velocity; ABI, ankle-brachial index; WC, waist circumference

For peer review only

For peer review only - http://bmjopen.bmj.com/site/about/guidelines.xhtml

Figure 1S (Supplementary material). Data are given as medias estimadas de CAVI y baPWV por cuartiles de BMI, WHtR, CUN-BAE and BRI.

Adjusted for age (years), sex (0=male and 1=female), systolic blood pressure smoking (0=No and 1=Yes), atherogenic index, HbA1c, and METs/min/week

baPWV and CAVI levels were compared using an ANOVA.

Abbreviations: CAVI, cardio-ankle vascular index; ba-PWV, brachial-ankle pulse wave velocity; BMI, body mass index; WHtR, waist-to-height ratio; CUN-BAE, Clínica Universidad de Navarra - body adiposity estimator; BRI, body roundness index

References

1. Adams KF, Schatzkin A, Harris TB *et al.* (2006) Overweight, obesity, and mortality in a large prospective cohort of persons 50 to 71 years old. *N Engl J Med* **355**, 763-778.
2. Poirier P, Giles TD, Bray GA *et al.* (2006) Obesity and cardiovascular disease: pathophysiology, evaluation, and effect of weight loss: an update of the 1997 American Heart Association Scientific Statement on Obesity and Heart Disease from the Obesity Committee of the Council on Nutrition, Physical Activity, and Metabolism. *Circulation* **113**, 898-918.
3. Wohlfahrt P, Somers VK, Cifkova R *et al.* (2014) Relationship between measures of central and general adiposity with aortic stiffness in the general population. *Atherosclerosis* **235**, 625-631.
4. Turin TC, Kita Y, Rumana N *et al.* (2010) Brachial-ankle pulse wave velocity predicts all-cause mortality in the general population: findings from the Takashima study, Japan. *Hypertens Res* **33**, 922-925.
5. Maeda Y, Inoguchi T, Etoh E *et al.* (2014) Brachial-ankle pulse wave velocity predicts all-cause mortality and cardiovascular events in patients with diabetes: the Kyushu Prevention Study of Atherosclerosis. *Diabetes Care* **37**, 2383-2390.
6. Ikura K, Hanai K, Oka S *et al.* (2016) Brachial-ankle pulse wave velocity, but not ankle-brachial index, predicts all-cause mortality in patients with diabetes after lower extremity amputation. *J Diabetes Investig*.
7. Izuhara M, Shioji K, Kadota S *et al.* (2008) Relationship of cardio-ankle vascular index (CAVI) to carotid and coronary arteriosclerosis. *Circ J* **72**, 1762-1767.
8. Okura T, Watanabe S, Kurata M *et al.* (2007) Relationship between cardio-ankle vascular index (CAVI) and carotid atherosclerosis in patients with essential hypertension. *Hypertens Res* **30**, 335-340.
9. Nakamura K, Tomaru T, Yamamura S *et al.* (2008) Cardio-ankle vascular index is a candidate predictor of coronary atherosclerosis. *Circ J* **72**, 598-604.
10. Satoh-Asahara N, Kotani K, Yamakage H *et al.* (2015) Cardio-ankle vascular index predicts for the incidence of cardiovascular events in obese patients: a multicenter prospective cohort study (Japan Obesity and Metabolic Syndrome Study: JOMS). *Atherosclerosis* **242**, 461-468.
11. Wohlfahrt P, Krajcoviechova A, Seidlerova J *et al.* (2013) Lower-extremity arterial stiffness vs. aortic stiffness in the general population. *Hypertens Res* **36**, 718-724.
12. Moh MC, Sum CF, Lam BC *et al.* (2015) Evaluation of body adiposity index as a predictor of aortic stiffness in multi-ethnic Asian population with type 2 diabetes. *Diab Vasc Dis Res* **12**, 111-118.

For peer review only - http://bmjopen.bmj.com/site/about/guidelines.xhtml

13. Teoh WL, Price JF, Williamson RM *et al.* (2013) Metabolic parameters associated with arterial stiffness in older adults with Type 2 diabetes: the Edinburgh Type 2 diabetes study. *J Hypertens* **31**, 1010-1017.
14. Hansen TW, Jeppesen J, Rasmussen S *et al.* (2004) Relation between insulin and aortic stiffness: a population-based study. *J Hum Hypertens* **18**, 1-7.
15. Rodrigues SL, Baldo MP, Lani L *et al.* (2012) Body mass index is not independently associated with increased aortic stiffness in a Brazilian population. *Am J Hypertens* **25**, 1064-1069.
16. Huisman HW, Schutte R, Venter HL *et al.* (2015) Low BMI is inversely associated with arterial stiffness in Africans. *Br J Nutr* **113**, 1621-1627.
17. Canepa M, AlGhatrif M, Pestelli G *et al.* (2014) Impact of central obesity on the estimation of carotid-femoral pulse wave velocity. *Am J Hypertens* **27**, 1209-1217.
18. Scuteri A, Orru M, Morrell CH *et al.* (2012) Associations of large artery structure and function with adiposity: effects of age, gender, and hypertension. The SardiNIA Study. *Atherosclerosis* **221**, 189-197.
19. Johansen NB, Vistisen D, Brunner EJ *et al.* (2012) Determinants of aortic stiffness: 16-year follow-up of the Whitehall II study. *PLoS One* **7**, e37165.
20. Wildman RP, Mackey RH, Bostom A *et al.* (2003) Measures of obesity are associated with vascular stiffness in young and older adults. *Hypertension* **42**, 468-473.
21. Recio-Rodriguez JI, Gomez-Marcos MA, Patino-Alonso MC *et al.* (2012) Abdominal obesity vs general obesity for identifying arterial stiffness, subclinical atherosclerosis and wave reflection in healthy, diabetics and hypertensive. *BMC Cardiovasc Disord* **12**, 3.
22. Brunner EJ, Shipley MJ, Ahmadi-Abhari S *et al.* (2015) Adiposity, obesity, and arterial aging: longitudinal study of aortic stiffness in the Whitehall II cohort. *Hypertension* **66**, 294-300.
23. Marrugat J, Vila J, Baena-Diez JM *et al.* (2011) [Relative validity of the 10-year cardiovascular risk estimate in a population cohort of the REGICOR study]. *Rev Esp Cardiol* **64**, 385-394.
24. Marti R, Parramon D, Garcia-Ortiz L *et al.* (2011) Improving interMediAte risk management. MARK study. *BMC Cardiovasc Disord* **11**, 61.
25. Marrugat J, D'Agostino R, Sullivan L *et al.* (2003) An adaptation of the Framingham coronary heart disease risk function to European Mediterranean areas. *J Epidemiol Community Health* **57**, 634-638.
26. Conroy RM, Pyorala K, Fitzgerald AP *et al.* (2003) Estimation of ten-year risk of fatal cardiovascular disease in Europe: the SCORE project. *Eur Heart J* **24**, 987-1003.
27. Mancia G, Fagard R, Narkiewicz K *et al.* (2013) 2013 ESH/ESC Guidelines for the management of arterial hypertension: the Task Force for the management of arterial hypertension of the European Society of Hypertension (ESH) and of the European Society of Cardiology (ESC). *J Hypertens* **31**, 1281-1357.
28. Salas-Salvado J, Rubio MA, Barbany M *et al.* (2007) [SEEDO 2007 Consensus for the evaluation of overweight and obesity and the establishment of therapeutic intervention criteria]. *Med Clin (Barc)* **128**, 184-196. .
29. Browning LM, Hsieh SD, Ashwell M (2010) A systematic review of waist-to-height ratio as a screening tool for the prediction of cardiovascular disease and diabetes: 0.5 could be a suitable global boundary value. *Nutr Res Rev* **23**, 247-269.
30. Oliveros E, Somers VK, Sochor O *et al.* (2014) The concept of normal weight obesity. *Prog Cardiovasc Dis* **56**, 426-433.
31. Gomez-Ambrosi J, Silva C, Catalan V *et al.* (2012) Clinical usefulness of a new equation for estimating body fat. *Diabetes Care* **35**, 383-388.
32. Thomas DM, Bredlau C, Bosy-Westphal A *et al.* (2013) Relationships between body roundness with body fat and visceral adipose tissue emerging from a new geometrical model. *Obesity (Silver Spring)* **21**, 2264-2271.
33. Satoh N, Shimatsu A, Kato Y *et al.* (2008) Evaluation of the cardio-ankle vascular index, a new indicator of arterial stiffness independent of blood pressure, in obesity and metabolic syndrome. *Hypertens Res* **31**, 1921-1930.

For peer review only - http://bmjopen.bmj.com/site/about/guidelines.xhtml

34. Shirai K, Utino J, Otsuka K *et al.* (2006) A novel blood pressure-independent arterial wall stiffness parameter; cardio-ankle vascular index (CAVI). *J Atheroscler Thromb* **13**, 101-107.
35. Shirai K, Hiruta N, Song M *et al.* (2011) Cardio-ankle vascular index (CAVI) as a novel indicator of arterial stiffness: theory, evidence and perspectives. *J Atheroscler Thromb* **18**, 924-938.
36. Yamashina A, Tomiyama H, Takeda K *et al.* (2002) Validity, reproducibility, and clinical significance of noninvasive brachial-ankle pulse wave velocity measurement. *Hypertens Res* **25**, 359-364.
37. Catapano AL, Reiner Z, De Backer G *et al.* (2011) ESC/EAS Guidelines for the management of dyslipidaemias The Task Force for the management of dyslipidaemias of the European Society of Cardiology (ESC) and the European Atherosclerosis Society (EAS). *Atherosclerosis* **217**, 3-46.
38. O'Brien E, Asmar R, Beilin L *et al.* (2005) Practice guidelines of the European Society of Hypertension for clinic, ambulatory and self blood pressure measurement. *J Hypertens* **23**, 697-701.
39. Taylor HL, Jacobs DR, Jr., Schucker B *et al.* (1978) A questionnaire for the assessment of leisure time physical activities. *J Chronic Dis* **31**, 741-755.
40. Elosua R, Marrugat J, Molina L *et al.* (1994) Validation of the Minnesota Leisure Time Physical Activity Questionnaire in Spanish men. The MARATHOM Investigators. *Am J Epidemiol* **139**, 1197-1209.
41. Elosua R, Garcia M, Aguilar A *et al.* (2000) Validation of the Minnesota Leisure Time Physical Activity Questionnaire In Spanish Women. Investigators of the MARATDON Group. *Med Sci Sports Exerc* **32**, 1431-1437.
42. Ainsworth BE, Haskell WL, Leon AS *et al.* (1993) Compendium of physical activities: classification of energy costs of human physical activities. *Med Sci Sports Exerc* **25**, 71-80.
43. Haskell WL, Lee IM, Pate RR *et al.* (2007) Physical activity and public health: updated recommendation for adults from the American College of Sports Medicine and the American Heart Association. *Circulation* **116**, 1081-1093.
44. Steiger JH (1980) Tests for comparing elements of a correlation matrix. *Psychological Bulletin* **87**, 245-251.
45. (2013) World Medical Association Declaration of Helsinki: ethical principles for medical research involving human subjects. *JAMA* **310**, 2191-2194.
46. Choi SY, Oh BH, Bae Park J *et al.* (2013) Age-associated increase in arterial stiffness measured according to the cardio-ankle vascular index without blood pressure changes in healthy adults. *J Atheroscler Thromb* **20**, 911-923.
47. Tomiyama H, Yamashina A, Arai T *et al.* (2003) Influences of age and gender on results of noninvasive brachial-ankle pulse wave velocity measurement--a survey of 12517 subjects. *Atherosclerosis* **166**, 303-309.
48. Marlatt KL, Kelly AS, Steinberger J *et al.* (2013) The influence of gender on carotid artery compliance and distensibility in children and adults. *J Clin Ultrasound* **41**, 340-346.
49. Bartok CJ, Marini ME, Birch LL (2011) High body mass index percentile accurately reflects excess adiposity in white girls. *J Am Diet Assoc* **111**, 437-441.
50. Philip R, Alpert BS, Schwingshackl A *et al.* (2015) Inverse Relationship between Cardio-Ankle Vascular Index and Body Mass Index in Healthy Children. *J Pediatr* **167**, 361-365 e361.
51. Yeboah K, Antwi DA, Gyan B *et al.* (2016) Arterial stiffness in hypertensive and type 2 diabetes patients in Ghana: comparison of the cardio-ankle vascular index and central aortic techniques. *BMC Endocr Disord* **16**, 53.
52. Al-Daghri NM, Al-Attas OS, Wani K *et al.* (2015) Sensitivity of various adiposity indices in identifying cardiometabolic diseases in Arab adults. *Cardiovasc Diabetol* **14**, 101.
53. Gomez-Sanchez L, Garcia-Ortiz L, Patino-Alonso MC *et al.* (2016) Association of metabolic syndrome and its components with arterial stiffness in Caucasian subjects of the MARK study: a cross-sectional trial. *Cardiovasc Diabetol* **15**, 148.
54. Morigami H, Morioka T, Yamazaki Y *et al.* (2016) Visceral Adiposity is Preferentially Associated with Vascular Stiffness Rather than Thickness in Men with Type 2 Diabetes. *J Atheroscler Thromb* **23**, 1067-1079.

For peer review only - http://bmjopen.bmj.com/site/about/guidelines.xhtml

55. Kawada T, Andou T, Fukumitsu M (2014) Relationship between cardio-ankle vascular index and components of metabolic syndrome in combination with sex and age. *Diabetes Metab Syndr* **8**, 242-244.
56. Kim HL, Lee JM, Seo JB *et al.* (2015) The effects of metabolic syndrome and its components on arterial stiffness in relation to gender. *J Cardiol* **65**, 243-249.
57. Zhou F, Zhang H, Yao W *et al.* (2014) Relationship between brachial-ankle pulse wave velocity and metabolic syndrome components in a Chinese population. *J Biomed Res* **28**, 262-268.
58. Weng C, Yuan H, Yang K *et al.* (2013) Gender-specific association between the metabolic syndrome and arterial stiffness in 8,300 subjects. *Am J Med Sci* **346**, 289-294.
59. Strasser B, Arvandi M, Pasha EP *et al.* (2015) Abdominal obesity is associated with arterial stiffness in middle-aged adults. *Nutr Metab Cardiovasc Dis* **25**, 495-502.
60. Danias PG, Tritos NA, Stuber M *et al.* (2003) Comparison of aortic elasticity determined by cardiovascular magnetic resonance imaging in obese versus lean adults. *Am J Cardiol* **91**, 195-199.
61. Takaki A, Ogawa H, Wakeyama T *et al.* (2008) Cardio-ankle vascular index is superior to brachial-ankle pulse wave velocity as an index of arterial stiffness. *Hypertens Res* **31**, 1347-1355.
62. Shirai K (2011) Analysis of vascular function using the cardio-ankle vascular index (CAVI). *Hypertens Res* **34**, 684-685.
63. Petersen KS, Blanch N, Keogh JB *et al.* (2015) Effect of weight loss on pulse wave velocity: systematic review and meta-analysis. *Arteriosclerosis, thrombosis, and vascular biology* **35**, 243-252.
64. Petersen KS, Clifton PM, Lister N *et al.* (2016) Effect of weight loss induced by energy restriction on measures of arterial compliance: A systematic review and meta-analysis. *Atherosclerosis* **247**, 7-20.
65. Kubozono T, Miyata M, Ueyama K *et al.* (2007) Clinical significance and reproducibility of new arterial distensibility index. *Circ J* **71**, 89-94.
66. Nagayama D, Endo K, Ohira M *et al.* (2013) Effects of body weight reduction on cardio-ankle vascular index (CAVI). *Obes Res Clin Pract* **7**, e139-e145.

For peer review only

For peer review only - http://bmjopen.bmj.com/site/about/guidelines.xhtml

Table 1 General characteristics of all the sample and by gender.

Variables	Global (n=2354)	Males (n=1456)	Females (n=898)	p Value
Age (years)	61.4±7.7	61.1±8.1	61.8±7.0	0.030
Smoking n (%)	658 (28.0)	456 (31.5)	202 (22.7)	<0.001
Alcohol (gr/week)	72.2±117.5	102.2±133.3	23.6±59.9	<0.001
Physical activity (METs/min/week)	2481±2512	2886±1831	1825±1691	<0.001
Sedentary n (%)	1020 (43.3)	538 (37.0)	482 (53.7)	<0.001
Height (cm)	165±9	170±7	156±6	<0.001
Weight (kg)	79.4±14.6	83.9±13.4	72.2±13.3	<0.001
BMI (kg/m^2)	29.2±4.4	29.1±3.9	29.5±5.1	0.035
BMI ≥ 30 n (%)	847 (36.0)	485 (33.4)	362 (40.4)	0.001
Waist circumference (cm)	100.9±11.6	102.9±10.5	97.6±12.5	<0.001
WHtR	0.61±0.07	0.61±0.06	0.62±0.08	<0.001
CUN-BAE	35.7±1.7	31.1±4.5	43.1±5.1	<0.001
BRI	5.8±1.7	5.7±1.5	6.1±2.1	<0.001
SBP (mmHg)	137.1±17.4	138.9±17.1	134.2±17.5	<0.001
DBP (mmHg)	84.4±10.2	85.5±10.4	82.7±9.7	<0.001
Heart rate (beats minutes)	74.2±10.2	73.3±12.7	75.8±11.6	<0.001
Hypertension n (%)	1712 (72.7)	1122 (80.1)	590 (75.4)	<0.001
Antihypertensive drugs n (%)	1199 (50.9)	729 (50.2)	470 (52.6)	0.289
Total Cholesterol (mg/dl)	225.8±40.9	220.8±39.1	233.9±42.5	<0.001
LDL Cholesterol (mg/dl)	140.4±34.9	138.9±34.2	142.8±35.8	0.011
HDL Cholesterol (mg/dl)	49.8±12.9	47.9±11.9	52.9±13.8	<0.001
Triglycerides (mg/dl)	145.5±96.6	150.3±106.3	137.7±77.9	0.001
Atherogenic index	4.8±1.3	4.8±1.3	4.7±1.3	0.002
Dyslipidemia n (%)	2151 (91.4)	1311 (90.0)	840 (93.5)	<0.001
Lipid lowering drugs n (%)	671 (28.5)	392 (26.8)	279 (31.0)	0.034
FPG (mg/dl)	107.2±34.8	106.9±33.9	107.6±36.1	0.659
HbA1c	4.8±1.3	5.9±1.4	6.1±1.4	0.001
Diabetes n (%)	791 (33.6)	463 (31.8)	328 (36.5)	0.020
Antidiabetic drugs n (%)	474 (20.1)	269 (18.5)	205 (22.9)	0.011
CAVI	8.8±1.2	8.9±1.2	8.6±1.1	<0.001
baPWV (m/s)	14.9±2.5	14.8±2.5	15.0±2.6	0.107

Values are means and (standard deviations) for continuous data and number and (proportions) for categorical data.

Abbreviations: METs-min/week, metabolic equivalent minutes per week. BMI, body mass index. WHtR, waist-to-height ratio. CUN-BAE, Clínica Universidad de Navarra-body adiposity estimator. BRI, body roundness index. SBP, systolic blood pressure. DBP, diastolic blood pressure. LDL, low density lipoprotein. HDL, high density lipoprotein. FPG, fasting plasma glucose. HbA1c, glycosylated hemoglobin. CAVI, cardio ankle vascular index. baPWV, braquial-ankle pulse wave velocity.

p value differences between male and females

For peer review only

Table 2: Bivariate correlations of adiposity measures with CAVI and baPWV.

	CAVI		baPWV	
	Unadjusted	Adjusted [a]	Unadjusted	Adjusted [a]
**BMI**	-0.264**	-0.303**	-0.035	-0.068**
**WHtR**	-0.119**	-0.222**	0.090**	0.001
**CUN-BAE**	-0.187**	-0.297**	0.054*	-0.063**
**BRI**	-0.125**	-0.218**	0.078**	0.005

The correlation coefficients between CAVI, baPWV and adiposity measurements showed significant differences (p <0.001 in all cases).

Abbreviations: CAVI, cardio-ankle vascular index. baPWV, brachial-ankle pulse wave velocity. BMI, body mass index. WHtR, waist-to-height ratio. CUN-BAE, Clínica Universidad de Navarra-body adiposity estimator. BRI, body roundness index.

[a] Adjusted for age, sex and systolic blood pressure.

p-values by Pearson correlation.* p<0.05, ** p< 0.01.

For peer review only - http://bmjopen.bmj.com/site/about/guidelines.xhtml

Table 3: Multiple regression analysis: association between adiposity measures with CAVI and baPWV.

	R^2	Standardized β	No Standardized β(95%CI)	Partial R^2	p value
**CAVI**					
**BMI**	0.412	-0.289	-0.075 (-0.083 to -0.066)	0.055	<0.001
**WHtR**	0.381	-0.221	-3.650 (-4.207 to -3.093)	0.038	<0.001
**CUN-BAE**	0.410	-0.450	-0.069 (-0.077 to -0.061)	0.058	<0.001
**BRI**	0.378	-0.215	-0.142 (-0.165 to -0.120)	0.037	<0.001
**baPWV**					
**BMI**	0.402	-0.100	-0.057 (-0.076 to -0.038)	0.007	<0.001
**WHtR**	0.394	-0.044	-1.557 (-2.748 to -0.366)	0.001	0.021
**CUN-BAE**	0.401	-0.152	-0.050 (-0.068 to -0.033)	0.007	<0.001
**BRI**	0.394	-0.046	-0.066 (-0.115 to -0.018)	0.002	0.014

Four different multiple linear regression models were used to analyze the associations of adiposity measures with CAVI and baPWV.

Adjusted for age (years), gender (0=male and 1=female), systolic blood pressure, smoking (0=No and 1=Yes), atherogenic index, HbA1c and METs/min/week.

Abbreviations: CAVI, cardio-ankle vascular index. baPWV, brachial-ankle pulse wave velocity. BMI, body mass index. WHtR, waist-to-height ratio. CUN-BAE, Clínica Universidad de Navarra-body adiposity estimator. BRI, body roundness index. METs-min/week, metabolic equivalent minutes per week.

For peer review only

For peer review only - http://bmjopen.bmj.com/site/about/guidelines.xhtml

Table 4: Association between adiposity measures with CAVI in different groups.

	R^2	β(95% CI)	Partial R^2	p value
		**CAVI**		
Hypertensive				
**BMI**	0.409	-0.076 (-0.087 to -0.065)	0.023	<0.001
**WHtR**	0.383	-3.838 (-4.527 to -3.148)	0.035	<0.001
**CUN-BAE**	0.368	-0.068 (-0.078 to -0.058)	0.051	<0.001
**BRI**	0.382	-0.165 (-0.193 to -0.137)	0.035	<0.001
Non hypertensive				
**BMI**	0.372	-0.071 (-0.086 to -0.057)	0.052	<0.001
**WHtR**	0.324	-2.939 (-3.850 to -2.028)	0.046	<0.001
**CUN-BAE**	0.405	-0.065 (-0.080 to -0.052)	0.080	<0.001
**BRI**	0.318	-0.108 (-0.144 to -0.072)	0.042	<0.001
Diabetics				
**BMI**	0.439	-0.087 (-0.101 to -0.073)	0.95	<0.001
**WHtR**	0.399	-4.613 (-5.523 to -3.702)	0.069	<0.001
**CUN-BAE**	0.437	-0.083 (-0.096 to -0.070)	0.70	<0.001
**BRI**	0.391	-0.169 (-0.205 to -0.134)	0.062	<0.001
Non diabetics				
**BMI**	0.396	-0.067 (-0.078 to -0.056)	0.049	<0.001
**WHtR**	0.369	-3.088 (-3.781 to -2.396)	0.034	<0.001
**CUN-BAE**	0.395	-0.059 (-0.069 to -0.050)	0.064	<0.001
**BRI**	0.369	-0.130 (-0.159 to -0.099)	0.034	0.016
Obese				
**BMI**	0.414	-0.093 (-0.112 to -0.074)	0.054	<0.001
**WHtR**	0.377	-2.945 (-4.085 to -1.804)	0.003	<0.001
**CUN-BAE**	0.418	-0.100 (-0.122 to -0.079)	0.026	<0.001
**BRI**	0.377	-0.110 (-0.153 to -0.069)	0.003	<0.001
Non obese				
**BMI**	0.373	-0.058 (-0.078 to - 0.038)	0.013	<0.001
**WHtR**	0.361	-1.394 (-2.366 to - 0.421)	0.003	0.005
**CUN-BAE**	0.373	-0.050 (-0.066 to -0.033)	0.020	<0.001
**BRI**	0.358	-0.044 (-0.085 to -0.003)	0.002	0.034
≤ 62 years				
**BMI**	0.368	-0.078 (-0.089 to -0.077)	0.77	0.003
**WHtR**	0.332	-4.142 (-4.854 to -3.431)	0.065	<0.001
**CUN-BAE**	0.361	-0.068 (-0.078 to -0.058)	0.070	<0.001
**BRI**	0.324	-0.163 (-0.192 to -0.134)	0.062	<0.001
> 62 years				
**BMI**	0.234	-0.074 (-0.088 to -0.060)	0.056	<0.001
**WHtR**	0.195	-3.086 (-3.948 to -2.224)	0.029	<0.001
**CUN-BAE**	0.234	-0.071 (-0.084 to -0.059)	0.065	<0.001
**BRI**	0.193	-0.124 (-0.159 to -0.089)	0.028	<0.001
Assets				
**BMI**	0.414	-0.072 (-0.084 to -0.060)	0.053	<0.001
**WHtR**	0.392	-3.570 (-4.335 to -2.805)	0.034	<0.001
**CUN-BAE**	0.413	-0.066 (-0.075 to -0.053)	0.060	<0.001
**BRI**	0.391	-0.148 (-0.180 to -0.116)	0.034	<0.001
Sedentary				
**BMI**	0.408	-0.080 (-0.092 to -0.068)	0.055	<0.001
**WHtR**	0.364	-3.776 (-4.598 to -2.954)	0.045	<0.001
**CUN-BAE**	0.404	-0.074 (-0.086 to -0.062)	0.059	<0.001
**BRI**	0.360	-0.144 (-0.176 to -0.111)	0.043	<0.001

Multiple linear regression models were used to analyze the associations of adiposity measures with CAVI by groups.

Adjusted for age (years), gender (0=male and 1=female), Systolic blood pressure, smoking (0=No and 1=Yes), atherogenic index, HbA1c and METs/min/week.

For peer review only

For peer review only - http://bmjopen.bmj.com/site/about/guidelines.xhtml

Abbreviations: METs-min/week, metabolic equivalent minutes per week. CAVI, cardio-ankle vascular index. BMI, body mass index. WHtR, waist-to-height ratio. CUN-BAE, Clínica Universidad de Navarra-body adiposity estimator. BRI, body roundness index.

For peer review only

For peer review only - http://bmjopen.bmj.com/site/about/guidelines.xhtml

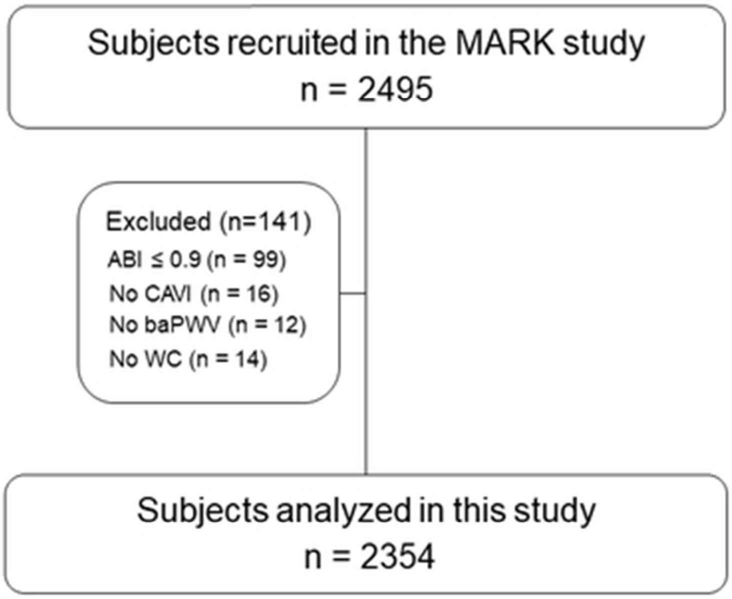

Figure 1. Flow chart of this MARK substudy
Abbreviations: N, number; CAVI, cardio-ankle vascular index; baPWV, brachial-ankle pulse wave velocity; ABI, ankle-brachial index; WC, waist circumference

42x40mm (300 x 300 DPI)

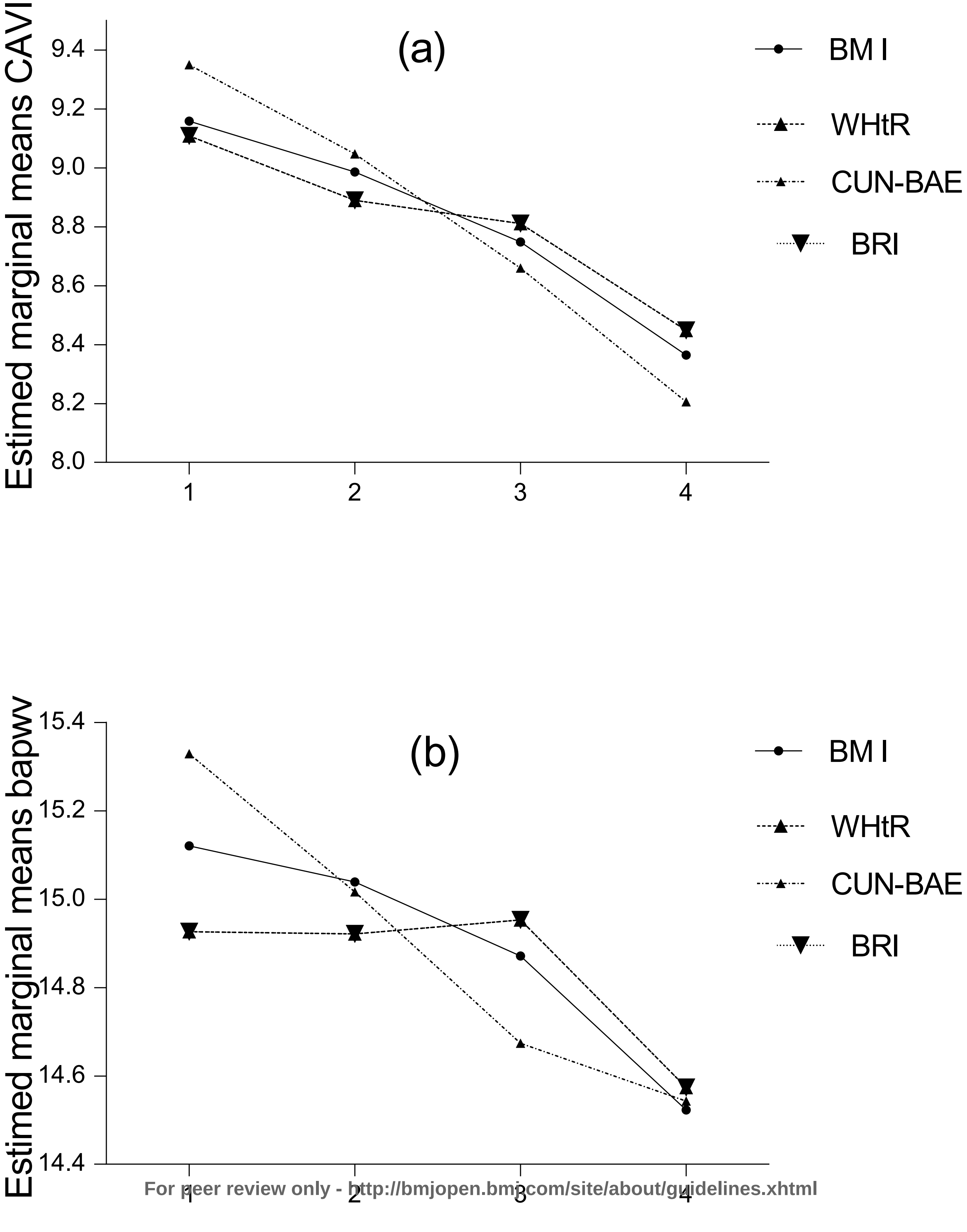
(a)
Estimed marginal means CAVI
9.4
9.2
9.0
8.8
8.6
8.4
8.2
8.0
1
2
3
4
BM I
WHtR
CUN-BAE
BRI
(b)
Estimed marginal means bapwv
15.4
15.2
15.0
14.8
14.6
14.4
1
2
3
4
BM I
WHtR
CUN-BAE
BRI

STROBE Statement—checklist of items that should be included in reports of observational studies

	Item No	Recommendation
**Title and abstract**	1	(*a*) Indicate the study's design with a commonly used term in the title or the abstract<br>**Yes, Page 1**
		(*b*) Provide in the abstract an informative and balanced summary of what was done and what was found<br>**Yes, Page 2**
**Introduction**		
Background/rationale	2	Explain the scientific background and rationale for the investigation being reported<br>**Yes, Page 4**
Objectives	3	State specific objectives, including any prespecified hypotheses<br>**Yes, Page 4**
**Methods**		
Study design	4	Present key elements of study design early in the paper<br>**Yes, Page 5**
Setting	5	Describe the setting, locations, and relevant dates, including periods of recruitment, exposure, follow-up, and data collection<br>**Yes, Page 5**
Participants	6	(*a*) *Cohort study*—Give the eligibility criteria, and the sources and methods of selection of participants. Describe methods of follow-up<br>*Case-control study*—Give the eligibility criteria, and the sources and methods of case ascertainment and control selection. Give the rationale for the choice of cases and controls<br>*Cross-sectional study*—Give the eligibility criteria, and the sources and methods of selection of participants<br>**Yes, Page 5**
		(*b*) *Cohort study*—For matched studies, give matching criteria and number of exposed and unexposed<br>*Case-control study*—For matched studies, give matching criteria and the number of controls per case<br>**Yes, Page 5**
Variables	7	Clearly define all outcomes, exposures, predictors, potential confounders, and effect modifiers. Give diagnostic criteria, if applicable<br>**Yes, Page 6**
Data sources/ measurement	8*	For each variable of interest, give sources of data and details of methods of assessment (measurement). Describe comparability of assessment methods if there is more than one group<br>**Yes, Page 6-9**
Bias	9	Describe any efforts to address potential sources of bias<br>**Yes, Page 8-9**
Study size	10	Explain how the study size was arrived at<br>**Yes, Page 5**
Quantitative variables	11	Explain how quantitative variables were handled in the analyses. If applicable, describe which groupings were chosen and why<br>**Yes, Page 8-9**

For peer review only - http://bmjopen.bmj.com/site/about/guidelines.xhtml

Statistical methods	12	(*a*) Describe all statistical methods, including those used to control for confounding **Yes, Page 8-9**
		(*b*) Describe any methods used to examine subgroups and interactions **Yes, Page 8-9**
		(*c*) Explain how missing data were addressed **Yes, Page 8-9**
		(*d*) *Cohort study*—If applicable, explain how loss to follow-up was addressed<br>*Case-control study*—If applicable, explain how matching of cases and controls was addressed<br>*Cross-sectional study*—If applicable, describe analytical methods taking account of sampling strategy
		(*e*) Describe any sensitivity analyses **Yes, Page 8-9**

Continued on next page

For peer review only

Results		
Participants	13*	(a) Report numbers of individuals at each stage of study—eg numbers potentially eligible, examined for eligibility, confirmed eligible, included in the study, completing follow-up, and analysed **Yes, Page 10-11**
		(b) Give reasons for non-participation at each stage **Yes, Page 10-11**
		(c) Consider use of a flow diagram **Yes, Figure 1**
Descriptive data	14*	(a) Give characteristics of study participants (eg demographic, clinical, social) and information on exposures and potential confounders **Yes, Page 10**
		(b) Indicate number of participants with missing data for each variable of interest **Yes, Page 10**
		(c) *Cohort study*—Summarise follow-up time (eg, average and total amount)
Outcome data	15*	*Cohort study*—Report numbers of outcome events or summary measures over time
		Case-control study—Report numbers in each exposure category, or summary measures of exposure
		Cross-sectional study—Report numbers of outcome events or summary measures
Main results	16	(*a*) Give unadjusted estimates and, if applicable, confounder-adjusted estimates and their precision (eg, 95% confidence interval). Make clear which confounders were adjusted for and why they were included **Yes, Tables**
		(*b*) Report category boundaries when continuous variables were categorized **Yes, tables**
		(*c*) If relevant, consider translating estimates of relative risk into absolute risk for a meaningful time period
Other analyses	17	Report other analyses done—eg analyses of subgroups and interactions, and sensitivity analyses **Yes, Page 10**
**Discussion**		
Key results	18	Summarise key results with reference to study objectives **Yes, Page 11**
Limitations	19	Discuss limitations of the study, taking into account sources of potential bias or imprecision. Discuss both direction and magnitude of any potential bias **Yes, Page 11**
Interpretation	20	Give a cautious overall interpretation of results considering objectives, limitations, multiplicity of analyses, results from similar studies, and other relevant evidence **Yes, Page 11-13**
Generalisability	21	Discuss the generalisability (external validity) of the study results **Yes, Page 11**
**Other information**		
Funding	22	Give the source of funding and the role of the funders for the present study and, if applicable, for the original study on which the present article is based **Yes, Page 15**

*Give information separately for cases and controls in case-control studies and, if applicable, for exposed and unexposed groups in cohort and cross-sectional studies.

Note: An Explanation and Elaboration article discusses each checklist item and gives methodological background and published examples of transparent reporting. The STROBE checklist is best used in conjunction with this article (freely available on the Web sites of PLoS Medicine at http://www.plosmedicine.org/, Annals of Internal Medicine at http://www.annals.org/, and Epidemiology at http://www.epidem.com/). Information on the STROBE Initiative is available at www.strobe-statement.org.

For peer review only

For peer review only - http://bmjopen.bmj.com/site/about/guidelines.xhtml
